# Radiative Cooling Materials for Extreme Environmental Applications

**DOI:** 10.1007/s40820-025-01835-9

**Published:** 2025-07-07

**Authors:** Jianing Xu, Wei Xie, Hexiang Han, Chengyu Xiao, Jing Li, Yifan Zhang, Shaowen Chen, Binyuan Zhao, Di Zhang, Han Zhou

**Affiliations:** 1 State Key Lab of Metal Matrix Composites, School of Materials Science and Engineering, Shanghai Jiao Tong University, Shanghai, 200240 People’s Republic of China; 2 Shanghai Institute of Spacecraft Equipment, Shanghai Academy of Spaceflight Technology, Shanghai, 200240 People’s Republic of China; 3 Future Materials Innovation Center, Zhangjiang Institute for Advanced Study, Shanghai Jiao Tong University, Shanghai, 201203 People’s Republic of China

**Keywords:** Extreme environment, Radiative cooling material, Micro-nano structure, Heat exchange channel, Latent heat

## Abstract

Radiative cooling is a passive thermal management strategy that leverages the natural ability of materials to dissipate heat through infrared radiation. It has significant implications for energy efficiency, climate adaptation, and sustainable technology development, with applications in personal thermal management, building temperature regulation, and aerospace engineering. However, radiative cooling performance is susceptible to environmental aging and special environmental conditions, limiting its applicability in extreme environments. Herein, a critical review of extreme environmental radiative cooling is presented, focusing on enhancing environmental durability and cooling efficiency. This review first introduces the design principles of heat exchange channels, which are tailored based on the thermal flow equilibrium to optimize radiative cooling capacity in various extreme environments. Subsequently, recent advancements in radiative cooling materials and micro-nano structures that align with these principles are systematically discussed, with a focus on their implementation in terrestrial dwelling environments, terrestrial extreme environments, aeronautical environments, and space environments. Moreover, this review evaluates the cooling effects and anti-environmental abilities of extreme radiative cooling devices. Lastly, key challenges hindering the development of radiative cooling devices for extreme environmental applications are outlined, and potential strategies to overcome these limitations are proposed, aiming to prompt their future commercialization.

## Highlights

- Heat exchange mechanisms for enhancing cooling performance and environmental tolerance are elucidated.
- Challenges in extreme environments, along with the corresponding anti-environmental radiative cooling materials and micro-nano structures, are reviewed.
- Valuable insights into enhancing the next generation of radiative cooling for extreme environmental applications are discussed.

## Graphical abstract

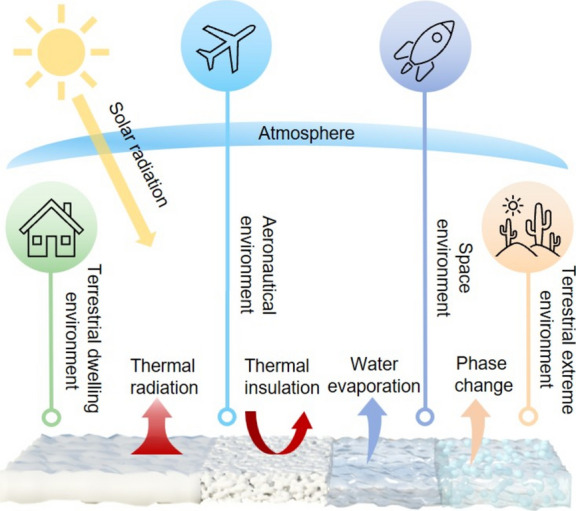

## Introduction

Radiative cooling has the ability to harvest coldness from the cold sink of outer space through thermal radiation in a passive zero-energy process. Fundamentally, it operates by dissipating thermal energy from the surface directly in the form of middle infrared (MIR) light while reflecting solar irradiance (0.25–2.5 μm) to ensure that the cooling power generated by MIR emission exceeds the heating effect of absorbed solar energy [1]. Applications of radiative cooling span diverse fields, including building energy management, where it reduces air conditioning loads and lower energy consumption; high-temperature environments, where it facilitates the maintenance of equipment efficiency by mitigating overheating issues; and space technologies, where it plays a critical role in thermal regulation for satellites, space probes, and other extraterrestrial structures [2, 3]. With continuous advancements, radiative cooling is emerging as a promising pathway to address critical challenges in climate resilience and energy consumption.

**Fig. 1:**
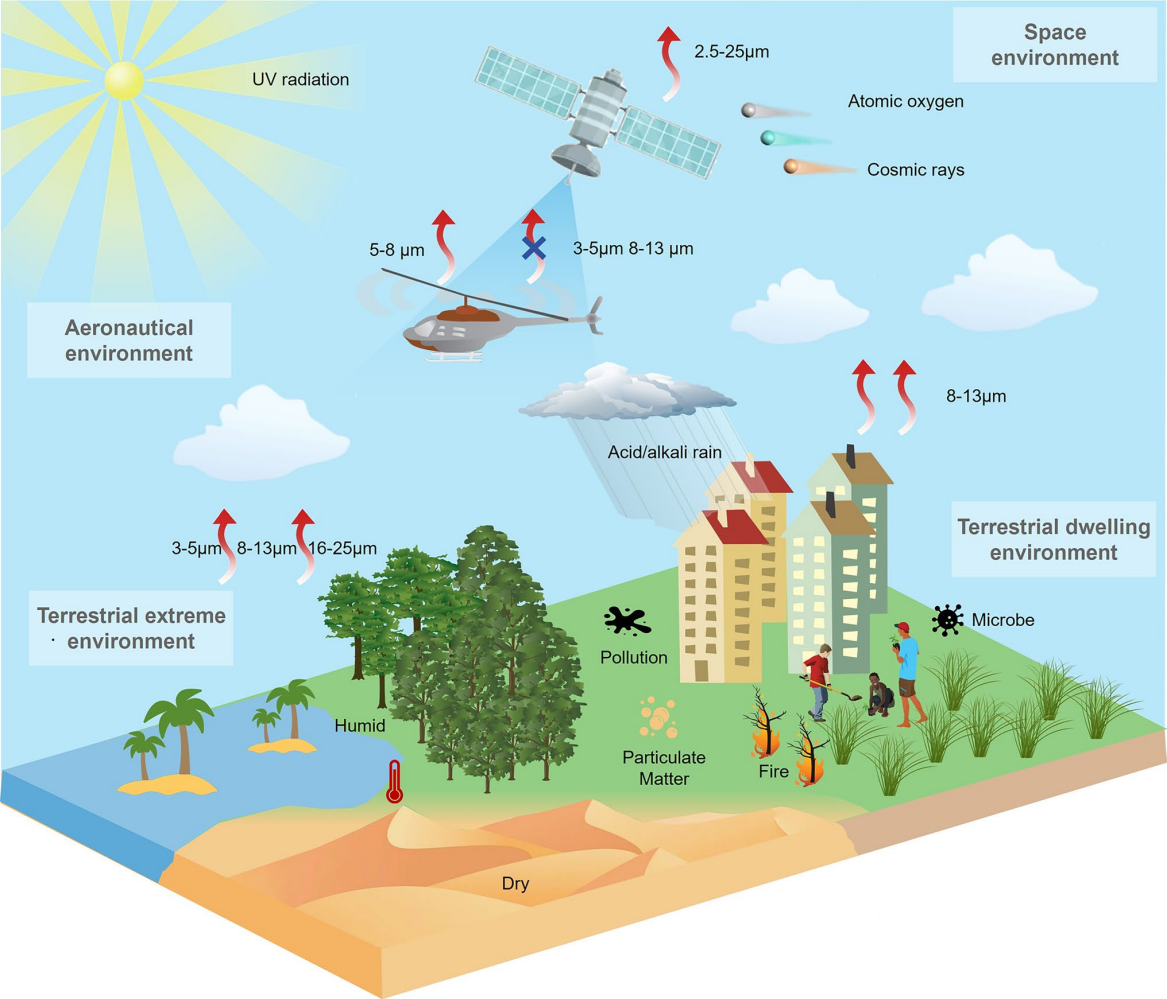
Schematic diagram of radiative cooling in different extreme environmental conditions. In terrestrial dwelling environments, radiative cooling devices emit in the 8–13 μm AW while resisting environmental damage such as microbes, UV radiation, contamination, flames, acid/alkali rain, and PM. In terrestrial extreme environments, coolers utilize more AWs (3–5, 8–13, and 16–25 μm) to maintain high performance under high-temperature, humid, or arid conditions. In aeronautical environments, thermal radiation is confined to the 5–8 μm range to meet infrared camouflage requirements. In space, coolers utilize the entire MIR spectrum and withstand harsh conditions such as strong UV radiation, cosmic rays, and atomic oxygen exposure for long-term operation

Recently, the development of radiative cooling materials and photonic structures has increasingly centered on their practical applications, particularly in extreme environments that impose distinct material and performance requirements. These environments can be broadly categorized into terrestrial dwelling environments, terrestrial extreme environments, aeronautical environments, and space environments, each demanding unique design considerations for radiative cooling materials and micro-nano structures (Fig. 1). Specifically, in terrestrial dwelling environments, which are suitable for human habitation but subject to external factors that can impact material performance, radiative cooling devices are required to emit MIR radiation in the primary atmospheric window (AW) range of 8–13 μm, while resisting various forms of environmental damage encountered in daily life, including bacteria, ultraviolet (UV) radiation, contamination, flames, acid/alkali rain, and particulate matter (PM) [4–7]. In terrestrial extreme environments, characterized by high temperatures and high humidity, which are unsuitable for habitation, it is necessary to introduce additional heat exchange pathways, such as secondary atmospheric windows (3–5 and 16–25 μm), evaporation, and phase change mechanisms [8–10]. In aeronautical environments, which involve high-altitude or high-speed flight where maintaining a low infrared signature is essential to avoid detection, keeping infrared camouflage is a crucial requirement for radiative cooling systems. To minimize the infrared signal intensity, radiative cooling in these applications is restricted to non-atmospheric windows (non-AWs), particularly in the 5–8 μm range [11]. In space environments, such as those encountered by satellites and spacecraft, radiative cooling devices offer unparalleled advantages by directly harvesting coldness from outer space across the entire MIR spectrum. However, for long-term operation, materials deployed in space must withstand harsh conditions, such as strong UV radiation, cosmic rays, and atomic oxygen exposure [12, 13].

In this review, we start by presenting the fundamental thermodynamic concept in the radiative cooling process as well as the principle governing the opening and closing of heat exchange channels for diverse extreme environmental applications. Building on this foundation, we provide an overview of diverse radiative cooling systems, emphasizing material properties and structural formation aimed at enhancing environmental tolerance and cooling performance. Remarkably, well-designed micro-nano structures, engineered by advanced fabrication techniques, enable precise spectral manipulation across the entire solar and MIR regions, optimizing radiative cooling performance. Moreover, the integration of hybrid cooling and additional heat exchange mechanisms extends the operational limits of radiative cooling systems, particularly in constrained conditions. Lastly, we highlight the remaining challenges in the field of extreme environmental radiative cooling (EERC) and provide our perspectives on addressing these obstacles.

## Design Principles of Heat Exchange Channels

Heat exchange in thermodynamics occurs between two objects at different temperatures, facilitated by the transfer of thermal energy. In the cooling management process, this exchange involves the inherent properties of the material and its interactions with the surrounding environment, including the hot solar radiation, the cold outer space, the ambient atmosphere, and the latent heat involved in phase changes such as evaporation or melting [14–16]. The process of heat flow follows the principles of thermodynamic equilibrium, where the net cooling power of a system can be represented by Eq. (1):

1 $$P_{{{\text{net}}}} = P_{{{\text{rad}}}} + P_{{{\text{eva}}}} + P_{{{\text{phase}}\,{\text{change}}}} - P_{{{\text{solar}}}} - P_{{{\text{atm}}}} - P_{{\text{cond + conv}}}$$

**Fig. 2:**
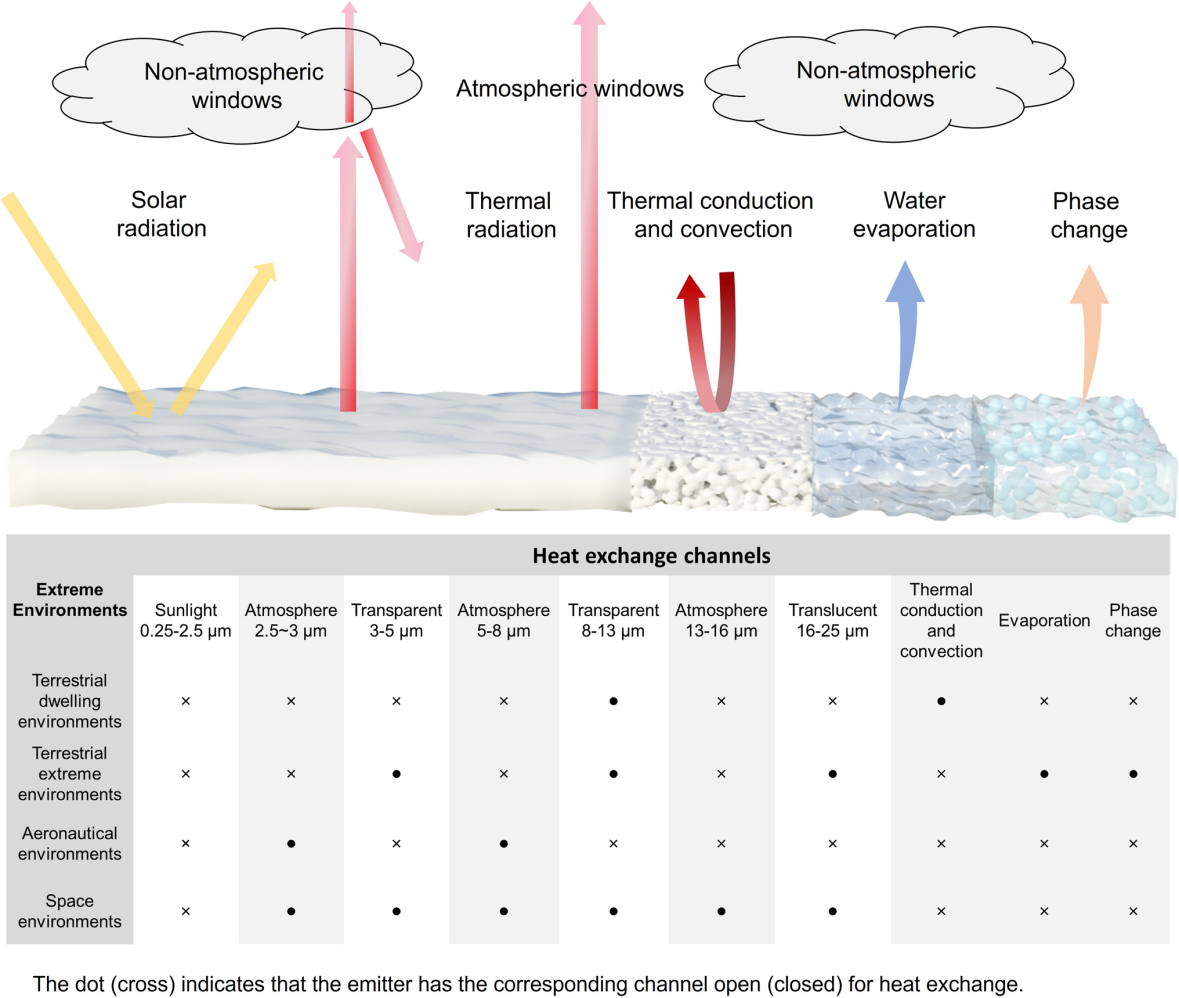
Schematic diagram of heat exchange channels, including solar radiation, thermal radiation through AWs (3–5, 8–13, and 16–25 μm), atmospheric inverse radiation (2.5–3, 5–8, and 13–16 μm), thermal conduction, thermal convection, water evaporation, and phase change cooling (top). The emitter has the corresponding open/close principle of heat exchange channels for different extreme conditions, including terrestrial dwelling environments, terrestrial extreme environments, aeronautical environments, and space environments (bottom)

*P*_net_ represents the net cooling power intensity of the surface, which is determined by the balance between various mechanisms of heat gain and heat dissipation. Specifically, a cooling management system absorbs heat from solar energy (*P*_*s*olar_), atmospheric radiation (*P*_atm_), and environmental parasitic heat from conduction and convection (*P*_cond+conv_), while it dissipates heat through thermal radiation (*P*_rad_), evaporation (*P*_eva_), and phase change processes (*P*_phase change_). An ideal cooler aims to maximize heat emission while minimizing heat absorption to efficiently reduce surface temperature. This requires a strategic “opening” and “closing” of different thermal channels: (1) opening dissipation channels, such as *P*_rad_, *P*_eva_, and *P*_phase change_, enhances the ability to release heat, rapidly lowering the surface temperature; (2) closing absorption channels, such as *P*_solar_, *P*_atm_, and *P*_cond+conv_, prevents external heat from being absorbed, maintaining lower operational temperatures even under intense environmental stress. In this section, we propose an open/close principle for managing heat dissipation pathways. By precisely coordinating the state of each heat exchange pathway, the cooling system achieves enhanced environmental adaptability and cooling efficiency (Fig. 2).

### Solar Irradiance Process

Solar energy serves as a significant source of heat for objects, causing their temperature to rise. *P*_solar_ represents the heat gain of objects under solar radiation, which can be calculated by Eq. (2) [17]:

2 $$P_{{{\text{solar}}}} = \mathop \int \limits_{0}^{\infty } I\left( \lambda \right)a_{r} \left( {\lambda ,\theta_{{{\text{sun}}}} } \right)d\lambda$$

where *I(λ)* denotes the solar radiation spectrum, *a*_*r*_* (λ, θ*_sun_*)* denotes the absorptance of the object at wavelength *λ* and angle *θ*_sun_. The absorptance defines the proportion of incoming solar radiation absorbed by the material, directly influencing the temperature rise.

Given that peak solar irradiance can exceed 1000 W m^–2^, even minimal solar energy absorption by cooling systems can significantly reduce the effectiveness of radiative cooling. Consequently, enhancing solar reflectance is crucial for preventing excessive heat absorption from solar radiation. To achieve this, it is necessary to employ specific electromagnetic reflection mechanisms to enhance the desired optical response. One such mechanism is Mie scattering, where the reflectivity of the structure is improved by optimizing its scattering cross-section. When Mie scattering is stimulated, incident photons are scattered by randomly distributed micro-nano units, such as pores, particles, or fibers, with sizes comparable to the wavelength of the incoming light [18]. In addition to Mie scattering, total internal reflection is another key mechanism that can be leveraged. Total internal reflection occurs when light propagates from an optically denser medium to an optically thinner medium at an angle greater than the critical angle, causing the light to be completely reflected back rather than refracted out [19]. Furthermore, photonic crystal structures can create photonic bandgaps that forbid the propagation of light within specific frequency ranges by introducing periodic variations in the refractive index [20]. This property enables precise spectral control, allowing photonic crystals to effectively modulate the solar spectrum and minimize unwanted solar absorption.

In practical applications, especially in areas such as architectural design, automotive coatings, or military camouflage, achieving broadband solar reflectance spectral profile may not always be the top priority. In these cases, there are loads of structurally colored radiative coolers developed to balance the colorful requirements and cooling performance, including SiO_2_ metasurfaces and cellulose nanocrystals that rely on optical diffraction, as well as multilayer structures and nanoparticles that utilize plasmon resonance [21–23].

### Radiative Process

Any object has the ability to transfer heat to the atmosphere and outer space through a thermal radiation process, and this transfer power can be quantitatively ensured by *P*_rad_ and* P*_atm_ [24]. *P*_rad_ is the total radiative power emitted by the object per unit area across all wavebands and directions, as described by Eq. (3):

3 $$P_{{{\text{rad}}}} = 2\pi \mathop \int \limits_{0}^{{\frac{\pi }{2}}} \sin \theta \cos \theta d\theta \mathop \int \limits_{0}^{\infty } I_{{{\text{BB}}}} \left( {\lambda , T} \right)\varepsilon_{r} \left( {\lambda , \theta } \right)d\lambda$$

where *ε*_*r*_*(λ, θ)* represents the emittance of the object at wavelength *λ* and angle *θ*, *I*_BB_(*λ*, *T*) represents the thermal radiation of a blackbody at temperature *T*, as described by Eq. (4):

4 $$I_{{{\text{BB}}}} \left( {\lambda , T} \right) = \frac{{2{\text{hc}}^{2} }}{{\lambda^{5} }}\frac{1}{{e^{{\frac{{{\text{hc}}}}{\lambda kT}}} - 1}}$$

where *h* represents the Planck constant, *k* represents the Boltzmann constant, and *c* represents the velocity of light. It is deduced that the emittance of the object is an essential factor governing its thermal radiation dissipation performance. To optimize emittance at thermal radiation wavelengths, various electromagnetic wave emission mechanisms have been proposed to tailor the radiative spectra of materials. For example, exploiting the molecular vibrational modes of chemical bonds and functional groups is a viable strategy. In particular, polymers possess a wide range of chemical bonds along their molecular chains, resulting in multiple resonant frequencies situated within the MIR region [25]. These vibrational resonances enable strong coupling with incident MIR photons, contributing to efficient thermal radiation dissipation. Apart from molecular vibrations, certain dielectric materials, such as SiO_2_, Si_3_N_4_, TiO_2_, and Al_2_O_3_, exhibit characteristic optical responses within the Reststrahlen band of the MIR spectrum. By incorporating engineered micro-nano architectures, such as periodic gratings and spherical nanoparticles, phonon polariton resonance can be excited to enhance emission in the desired MIR spectral region [26]. Another effective approach is the design of a graded-index surface at the air-medium interface. Due to the substantial refractive index mismatch between air and solid materials, significant Fresnel reflection occurs, which limits thermal emissions. By gradually varying the refractive index across the interface, the impedance mismatch can be minimized, thereby suppressing reflection and enhancing emission. Furthermore, additional electromagnetic resonance mechanisms, including surface-plasmon resonances, magnetic resonances, and Fabry-Perot (F-P) resonances, can be introduced through the use of metamaterials and multilayered structures [27]. Notably, in a state of thermal equilibrium, the emittance of the object is equal to its absorptance according to Kirchhoff’s law.

**Fig. 3:**
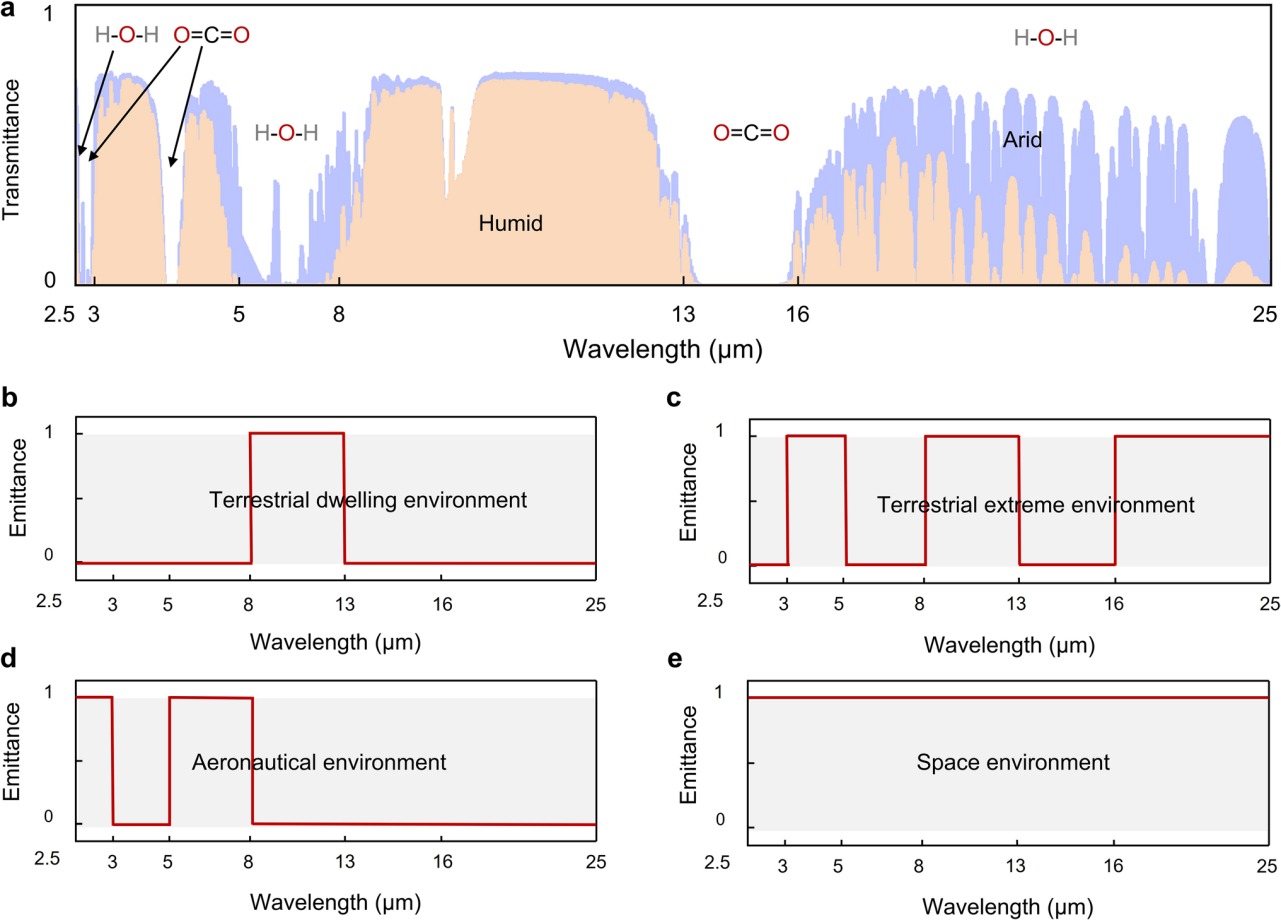
**a** Atmospheric transmittance in arid and humid conditions, and absorption regions of water vapor and carbon dioxide. The spectral requirement of radiative cooling in **b** terrestrial dwelling environments, **c** terrestrial extreme environments, **d** aeronautical environments, and **e** space environments

*P*_atm_ indicates the influence of atmospheric inverse radiation on objects, as described by Eq. (5):

5 $$P_{{{\text{atm}}}} = 2\pi \mathop \int \limits_{0}^{{\frac{\pi }{2}}} \sin \theta \cos \theta d\theta \mathop \int \limits_{0}^{\infty } I_{{{\text{BB}}}} \left( {\lambda , T_{{{\text{amb}}}} } \right)\varepsilon_{r} \left( {\lambda , \theta } \right)\varepsilon_{{{\text{atm}}}} \left( {\lambda , \theta } \right)d\lambda$$

where *T*_amb_ denotes the ambient temperature, and *ε*_atm_ *(λ, θ)* denotes the atmospheric emissivity influenced by moisture and clouds in the air. In several specific wavelength ranges, *ε*_atm_ *(λ, θ)* is near zero. Consequently, the atmosphere in these regions is virtually transparent to thermal radiation, which is called atmospheric windows. These atmospheric windows (3-5, 8-13, and 16-25 μm) allow thermal radtion from the Earth’s surface to pass directly into space [28]. Among that, the wavelength range of approximately 8–13 μm is the most prominent atmospheric window in the MIR region. Within this range, absorption by atmospheric gases such as water vapor and carbon dioxide is minimal, enabling efficient heat transfer from the ground to space. By contrast, the propagation of thermal radiation is hindered in other wavebands, known as non-atmospheric windows, due to the molecular vibrations of gases such as CO_2_ and H_2_O (Fig. 3a) [29].

In terrestrial dwelling environments, most objects have temperatures ranging from − 50 to 80 °C. This corresponds to the peak wavelength of the object’s radiation energy falling within the 8-13 µm range, as explained by Wien’s displacement law (Eq. (6)) [30]:

6 $$\lambda_{m} T = b$$

where *λ*_*m*_ represents the peak wavelength of the radiation, *T* represents the temperature of the blackbody, and *b* represents the Wien’s displacement constant, at about 0.002897 m K. Therefore, it is deduced that high emissivity in the waveband of 8-13 μm plays a significant role in maximizing cooling efficiency in terrestrial dwelling environments (Fig. 3b).

Notably, when the relative humidity is very low, atmospheric transmittance in the 16-25 μm range increases significantly, transitioning this atmospheric window from translucent to transparent [31]. In terrestrial extreme environments, such as the Gobi and desert, the low humidity levels make it possible to exploit not only the primary atmospheric window (8-13) but also secondary windows, including the 3–5 and 16-25 μm ranges. This enables enhanced radiative heat dissipation, which is beneficial for meeting the high cooling demands typically encountered under such extreme conditions (Fig. 3c).

Moreover, in aeronautical environments, where both infrared camouflage and radiative cooling are required, low infrared emissivity should be maintained in AWs to prevent infrared detectors from detecting strong infrared signals. Consequently, heat dissipation channels should shift from AWs to non-AWs to ensure that the infrared signals of objects resemble those of the ambient environment [32, 33]. Based on the principles of radiative process (Eqs. (3), (4) and 5) and Wien’s displacement law (Eq. (6)), the higher surface temperatures of objects in aeronautical environments cause the peak wavelength of radiation to shift to shorter wavelengths compared to terrestrial objects. As a result, achieving high emissivity within non-AWs in the shortwave region, such as 2.5-3 and 5-8 μm, is crucial for effective cooling management in aeronautical environments (Fig. 3d). Lastly, in space environments, due to the absence of the atmosphere, the power input from the atmosphere can drop to zero (*P*_atm_ = 0). Therefore, thermal radiation across the entire MIR region can be directly emitted from coolers to outer space. In this case, space cooling devices should be designed as ultrabroadband (> 2.5 μm) thermal radiators (Fig. 3e) [34].

### Thermal Conduction and Convection Process

Thermal convection is a process that propagates the heat from areas of high heat concentration to areas of lower heat concentration through the movement of fluids, such as gases or liquids. The heat transfer during thermal convection can be expressed by Eq. (7) [35]:

7 $$P_{{{\text{conv}}}} = h_{{{\text{conv}}}} \left( {T_{s} - T_{{{\text{amb}}}} } \right)$$

where *P*_conv_ is the heat flux of thermal convection, and *h*_conv_ represents the convective heat transfer coefficient, which depends on the properties of the fluid, the flow conditions, and the geometric configuration. *T*_*s*_ represents the surface temperature of the material. *T*_amb_ represents the absolute temperature of the fluid far away from the solid surface.

In addition to thermal convection between materials and ambient environments, thermal conduction plays a crucial role in heat exchange processes. The thermal conduction process allows environmental parasitic heat to penetrate the material and influence temperature on the opposite side. The heat flux within materials can be explained by the following equation [36, 37]:

8 $$P_{{{\text{cond}}}} = \frac{{k_{{{\text{cond}}}} }}{d}\left( {T_{s} - T_{{{\text{obj}}}} } \right)$$

where *P*_cond_ is the heat flux of thermal conduction, *k*_cond_ represents the thermal conductivity of the material, and *d* represents the thickness of the material. *T*_obj_ represents the temperature of the object being cooled. Hence, the total heat input through the thermal conduction and convection process (*P*_cond+conv_) can be expressed by Eq. (9):

9 $$P_{{\text{cond + conv}}} = h_{{{\text{conv}}}} \left( {T_{s} - T_{{{\text{amb}}}} } \right) + \frac{{k_{{{\text{cond}}}} }}{d}\left( {T_{s} - T_{{{\text{obj}}}} } \right)$$

Based on Eq. (9), we can deduce that the efficiency of thermal convection and conduction depends on the convective heat transfer coefficient, the thermal conductivity, the material thickness, and the temperature differences between the object, the surrounding fluid, and the ambient environment [38].

**Fig. 4:**
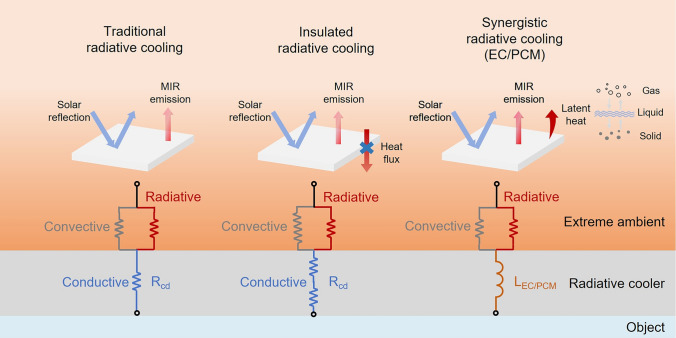
Traditional radiative coolers reflect solar energy and emit thermal radiation. Heat dissipation occurs by conduction (blue paths), convection (gray paths), and thermal radiation (red paths). Insulated radiative coolers have lower thermal conduction and convection coefficients compared to traditional radiative coolers, resulting in higher thermal resistance (blue and gray paths) to block heat flux into the interior. Synergistic radiative coolers can resist rapid variations in heat flow through EC or PCM (brown paths). This process is driven by the latent heat generated during water evaporation or solid-to-liquid phase change

In high-temperature environments, due to the large temperature difference between ambient environments and objects, a high volume of heat flow significantly raises the temperature of the cooled object. To counter the challenge, cooling management devices in extreme environments such as deserts, aeronautical settings, and space must be designed with low thermal convection and conduction coefficients (Fig. 4) [39–41]. These designs enhance thermal resistance to minimize heat transfer between external sources and interior objects, thereby reducing the overall heat load on the cooling system. However, in space environments, the absence of a heat transfer medium eliminates thermal convection. In such cases, designing effective thermal insulation materials is crucial to prevent heat flow from exterior surfaces into the interior of the object.

### Latent Heat Conversion Process

When the temperature changes, a material undergoes a phase transition, during which it either absorbs or releases heat in the form of latent heat. This latent heat property results in phase change processes that display characteristics similar to inductance, resisting rapid variations in heat flow and stabilizing thermal fluctuations (Fig. 4). For cooling management, it is common to employ liquid-to-gas or solid-to-liquid phase transitions in the design of cooling systems [42, 43].

Water, in particular, is an ideal candidate for the liquid-to-gas phase transition thanks to its high enthalpy of evaporation (*ΔH*_eva_ = 2260 kJ kg^−1^) and abundance. Through the evaporative process and mass exchange with an external medium (e.g., ambient air) in an open system, the vapor can carry away a large amount of waste heat to the ambient environment. The evaporative cooling (EC) power generated by water evaporation (*P*_EC_) can be calculated through the following equation [44]:

10 $$P_{{{\text{EC}}}} = \frac{{\Delta H_{{{\text{eva}}}} \cdot \Delta m_{{{\text{eva}}}} }}{{t_{{{\text{eva}}}} \cdot A_{{{\text{eva}}}} }}$$

where *t*_eva_ is the evaporation time, *Δm*_eva_ and *A*_eva_ represent the weight loss and the area of the materials, respectively. The term $\frac{{\Delta m_{{{\text{eva}}}} }}{{t_{{{\text{eva}}}} \cdot A_{{{\text{eva}}}} }}$ indicates the evaporation rate of water, which depends on the ambient temperature, relative humidity, and the structural design of the device.

In contrast, phase change cooling leverages the solid–liquid transition of phase change materials (PCMs) to regulate temperature through latent heat absorption, without involving any mass exchange. To prevent leakage during operation, PCMs are typically encapsulated within a sealed system. The generated cooling power (*P*_phase change_) can be calculated using Eq. (11) [45]:

11 $$P_{{{\text{phase }}\,{\text{change}}}} = \frac{{{\Delta }H_{{{\text{phase}}\,{\text{change}}}} \cdot m_{{{\text{phase}}\,{\text{change}}}} }}{{t_{{{\text{phase}}\,{\text{change}}}} \cdot A_{{{\text{phase}}\,{\text{change}}}} }}$$

where *t*_phase change_ is the phase change time, *m*_phase change_ and *A*_phase change_ represent the weight and the area of the phase change materials, respectively.

Evaporation and solid-to-liquid phase change processes offer additional heat dissipation channels, enhancing the cooling performance limits in extreme environments. Under high-temperature conditions, the latent heat of water and PCM can generate greater cooling power, effectively reducing the surface temperatures of cooled objects [46, 47]. Furthermore, in extreme environments with high humidity or cloud cover, the atmospheric windows for radiative cooling may be reduced or even blocked [48]. This results in a rapid increase in *P*_atm_ due to the rise in the atmospheric emissivity *ε*_atm_ (Eq. (5)), which reduces the overall cooling power. In such scenarios, combining evaporation or phase change processes with radiative cooling can help offset these losses.

## Terrestrial Dwelling Environment

In terrestrial dwelling environments, radiative coolers face a variety of extreme environments that affect human daily life and material performance, such as UV radiation, microorganisms, air pollution, flames, contamination, and urban heat islands. In this section, we present an exhaustive summary of four material systems, including organic materials, organic-based hybrid materials, inorganic-based hybrid materials, and inorganic materials to achieve strong environmental endurance under various extreme conditions. On the one hand, materials with high-energy covalent and metallic bonds possess inherent environmental resistance, making them ideal for enhancing the durability of radiative coolers in extreme environments [49]. On the other hand, well-designed micro-nano structures have the ability to endow materials with particular environmental durability [50]. Importantly, these cooling materials possess near blackbody emissivity in the primary AW (8–13 μm), which contributes to maximum cooling efficiency [51, 52].

### Organic Material

Organic materials used in extreme environments typically exhibit inherent resistance to various harsh conditions due to their fundamental physical and chemical properties. The stability of their molecular structures enables them to endure challenges such as acid and alkali exposure, UV radiation, and high temperatures [53, 54]. Moreover, the design of customized micro-nano structures can enhance their ability to repel dust and pollutants [55]. Additionally, the distinct vibrational modes of molecular chains provide opportunities for selective absorption in the MIR range, making these materials highly suitable for terrestrial environments [25].

**Fig. 5:**
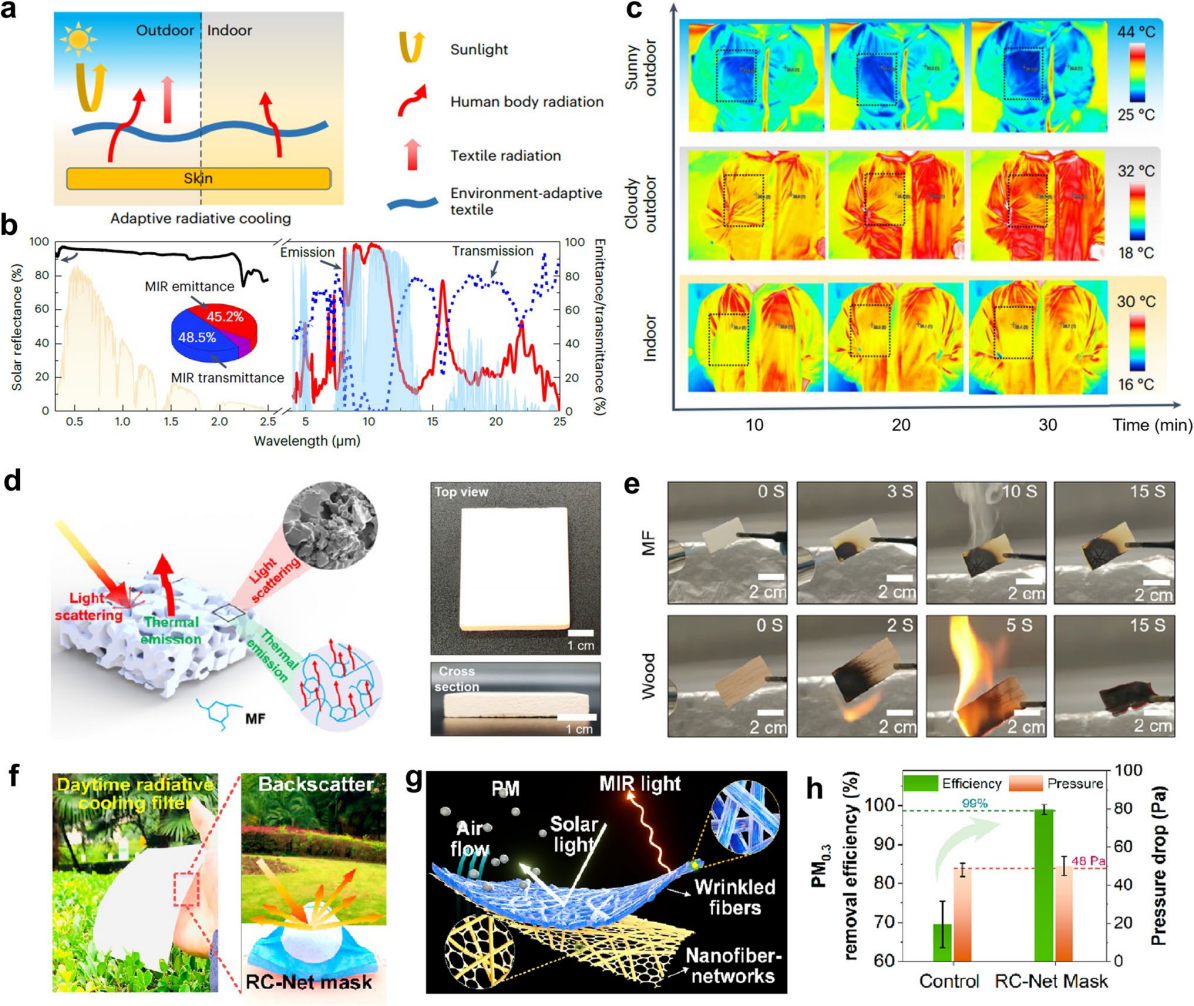
**a** Schematic of the textile for personal cooling. **b** Spectral properties of the POM textile across the solar spectrum MIR spectra. Inset shows the proportions of emittance, transmittance, and reflectance in the MIR spectrum. **c** Infrared images of the human body wearing protective clothing in three different settings. **d** Schematic of the nanostructure and cooling principle of the MF bulk (left). Photographs of the MF bulk with a white appearance (right). **e** Schematic diagram of the fire resistance test for the MF bulk and wood. **f** Optical image of a radiative cooling mask, exhibiting the intense scattering effect during the daytime. **g** Schematic of the radiative cooling mask with a gradient pore structure. **h** Evaluation of PM filtration performance and comfort of two masks. **a-c** Reproduced with permission [54]. Copyright 2023, Springer Nature. **d-e** Reproduced with permission [53]. Copyright 2021, Elsevier. **f–h** Reproduced with permission [56]. Copyright 2022, American Chemical Society

In outdoor and indoor environments, personal cooling is required to exhibit different spectral characteristics. In indoor settings, heat dissipation requires transparency to MIR radiation from the human body, while outdoor cooling efficiency mainly depends on high emissivity within the primary AW and high solar reflectivity (Fig. 5a). Wu et al. prepared a polyoxymethylene (POM) nanotextile through the electrospinning method, which involves a cooling mechanism balancing emission and transmission modes [54]. The vibrational absorption wavelengths of POM predominantly lie within the primary AW. Consequently, it demonstrates a high selective emittance (75.7%) in the range of 8-13 μm, with a selectivity of 1.67 (ratio of emittance in 8-13 to 4-25 μm). Moreover, the suitable nanofiber sizes, random arrangement, and coarse surface of the POM nanofibers enhance the sunlight reflectance of the POM textile. It reflects nearly 95% of solar energy in the 0.3-2.5 μm range and transmits 48.5% of thermal radiation in the 4-25 μm range, supporting personal cooling regardless of the environment (Fig. 5b). Beyond its outstanding cooling effect, the POM textile also demonstrates excellent environmental durability, including high UV and abrasion resistance. In UV tests, the spectral characteristics of the POM textile remained unchanged after approximately 417 days of exposure. Similarly, after friction testing, its solar reflectance and mass ratio show no significant change. Crucially, it can be used to prepare protective clothing with cooling functions. As shown in Fig. 5c, during continuous outdoor testing, the POM textile shows a superior cooling effect compared to commercial protective clothing. The temperature difference between the two areas reached approximately 3.0 °C in sunny conditions and 1.5 °C in cloudy conditions. Wu et al. designed a durable multilayer silk textile (MST) to improve the outdoor environmental endurance of the resulting silk nanotextile [57]. The integration of multilayer specific textiles (commercial silk textile, silk nanotextile, and polytetrafluoroethylene (PTFE) textile) endows the MST with tolerance in various scenarios. After 80 h of UV exposure, the solar reflectance of the MST remained constant, demonstrating its excellent UV resistance. Remarkably, the anti-symmetric and symmetric stretching vibration absorptions of -CF_2_ in PTFE coincide with the primary AW.

High temperatures pose a significant challenge to organic materials, with combustion being especially severe. Tian et al. prepared a high-performance mine-formaldehyde (MF) cooling bulk via hydraulic press and thermal annealing [53]. The MF bulk demonstrates effective sunlight scattering due to the microparticles on its diffused surface, thereby achieving a high solar reflectance of 0.94. Meanwhile, the molecular vibrations of melamine rings and hydroxyl groups lead to a thermal emission of 0.95 in the primary AW (Fig. 5d). As a thermosetting plastic material, MF exhibits excellent fire-retardant properties and self-extinguishing capabilities. When ignited at 1430 °C for 3 s, the MF bulk has the capacity to extinguish within 15 s, while the wood had already turned to ash (Fig. 5e). Moreover, the MF maintains virtually unchanged solar reflectance and MIR emittance when exposed to acidic or alkaline conditions. Additionally, Jung et al. fabricated stable polydimethylsiloxane (PDMS) nanofibers through coaxial electrospinning, using them as radiative coolers for wearable electronics [58]. Due to the intrinsic thermosetting properties of PDMS, strong cross-link bonds among polymer chains can be formed after curing, allowing PDMS nanofibers to maintain the porous structure at high temperatures. Moreover, the PDMS nanofibers demonstrate high sunlight reflectance (94%) and high infrared emittance (96%), attributed to the hierarchically porous nanofiber structure and the Si-O-Si and Si-CH_3_ stretching vibrations, respectively.

By designing intricate structures, materials can acquire enhanced resistance to environmental factors. Liu et al. designed a radiative cooling mask by fabricating heterogeneous nanofibrous networks, which effectively combine cooling performance with the ability to mitigate PM pollution (Fig. 5f) [56]. The cooling layer consists of wrinkled poly(vinylidene fluoride-co-hexafluoropropene) (P(VDF-HFP)) nanofibers with a porous structure, effectively achieving sunlight reflection and infrared emission (Fig. 5g). The exceptional PM capture capacity is attributed to their large surface area and tiny pores, which effectively bind to and trap ultrafine PM. Compared to the control nanofiber filters that achieve 69.51% removal, the radiative cooling mask demonstrates a 30-fold reduction in PM_0.3_ leakage (99.02% removal), which is equivalent to the N99 mask standard (Fig. 5h). Besides, cold environments demand radiative cooling materials with strong solar reflectivity and high infrared emissivity to minimize thermal absorption and effectively prevent ice melting. Li et al. developed a hierarchically structured film using eco-friendly cellulose acetate (CA) to achieve high cooling performance [59]. The film combines strong mid-infrared emittance from molecular bond vibrations with tailored pores that scatter solar radiation, thereby reducing heat load on ice and enabling passive protection across different latitudes. The CA film shows a solar reflectance of 0.974 and a mid-infrared emittance of 0.92, with a peak in the atmospheric window. Under continuous natural sunlight for 5 days, the ice covered with the hierarchically designed CA film showed negligible melting, while the bare ice completely disappeared, demonstrating the film’s strong capability to prevent ice loss. Fan et al. introduced core–shell composite nanofibers with high radiative cooling performance [60]. The unique core-shell structure of poly(vinylidene fluoride) (PVDF)@polyethyleneimine (PEI) significantly improves properties such as thermal stability and hydrophobicity. It also boasts exceptional UV resistance and air permeability to excel across various scenarios. Meng et al. reported a durable superhydrophobic porous coating with nano-globules (SHPo-ME) designed for effective radiative cooling [61]. The interconnected nano-globules boost surface roughness, resulting in remarkable superhydrophobicity with a contact angle rising to 165° and a sliding angle reduced to 2.4°. Xu et al. prepared a porous PTFE coating with high solar reflectance (94%) and infrared emittance (93%) [62]. Notably, the porous PTFE coating offers an outstanding UV protection factor of 179.15 and enables energy harvesting even on rainy days, thanks to the PTFE-rich dielectric layer within the harvester.

### Organic-based Hybrid Material

Although several organic materials possess excellent environmental resistance, the majority of organic materials are susceptible to environmental influences that can lead to performance degradation. In contrast, inorganic materials inherently offer superior resistance to harsh environmental conditions [63]. Therefore, organic-based hybrid materials, comprising an organic matrix integrated with inorganic additives, have become an effective strategy for enhancing radiative cooling performance in extreme environments. In these hybrid systems, the organic component serves as the primary structural framework, enabling the formation of micro-nano structures for tailoring optical properties. Meanwhile, the inorganic additives act as functional enhancers, not only improving environmental durability but also contributing to strong absorptivity in the 8-13 μm range through phonon polariton resonance. Furthermore, benefiting from the micro-nano structures (fibers, particles, and pores) constructed from organic frameworks and inorganic components, the hybrid systems effectively scatter sunlight and achieve high solar reflectance [64]. Consequently, the integration of diverse materials and structures enables precise spectral regulation and imparts multiple characteristics to radiative cooling systems, making them more robust in extreme environments.

**Fig. 6:**
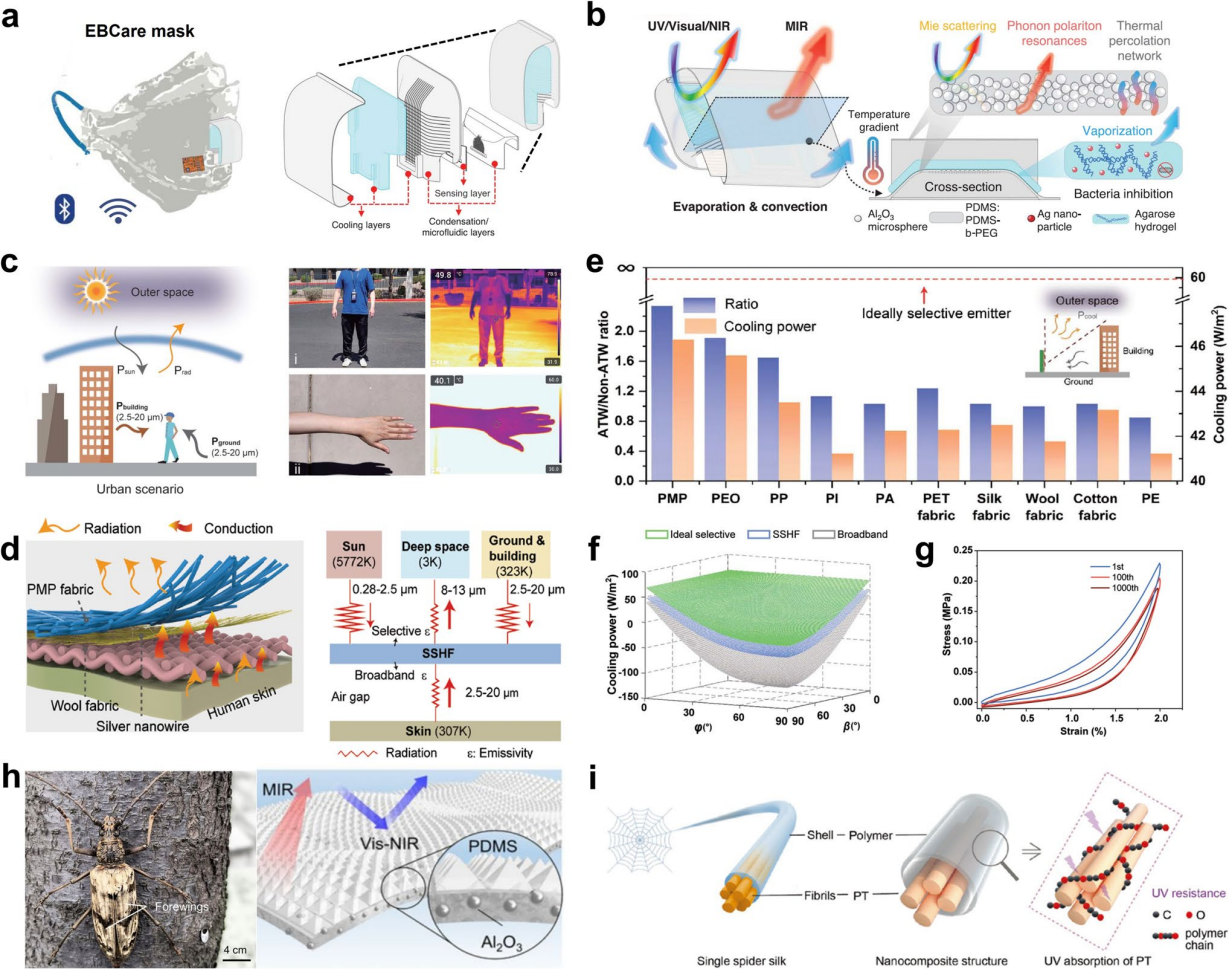
**a** Schematic of the smart EBCare, which possesses cooling and sensing functions. **b** Schematic of the EBCare cooling mechanism. **c** Schematic of wearable fabrics, with the vertical region occupying the majority, in an urban scenario (left). Optical and infrared images of typical scenarios where terrestrial objects function as heat sources (right). **d** Schematic of the structure and heat transfer mechanism of SSHF (left). Thermal radiation processes between SSHF, the skin, and the external environment (right). **e** Emittance selective ratio and calculated cooling power of PMP and other materials. **f** Calculated cooling power for SSHF, broadband, and selective emitters. **g** SSHF exhibits outstanding stability over 1000 tensile test cycles. **h** Photograph of a male longicorn beetle (left). Schematic of bioinspired films, where ceramic particles are integrated into a PDMS matrix with dense micropyramidal arrays (right). **i** Spider-silk-inspired nanocomposites with improved mechanical properties and UV resistance. **a, b** Reproduced with permission [65]. Copyright 2024, AAAS. **c-g** Reproduced with permission [51]. Copyright 2024, AAAS. **h** Reproduced with permission [19]. Copyright 2020, PNAS. **i** Reproduced with permission [66]. Copyright 2022, Wiley–VCH

Attributing to the properties of the added inorganic particles, radiative cooling systems can exhibit admirable antimicrobial properties to protect human health and maintain unpolluted surfaces. Heng et al. designed a mechanically soft microfluidic smart mask system (EBCare), employing a tandem cooling strategy that combines hydrogel evaporation with radiative cooling (Fig. 6a) [65]. The core structure of EBCare consists of a ceramic alumina-polymer hybrid metamaterial, which boasts excellent thermal conductivity and optimal radiative cooling characteristics. Additionally, the evaporation of water from the agarose hydrogel naturally draws heat from the surroundings. Significantly, incorporating Ag nanoparticles into the hydrogel not only imparts potent antimicrobial properties but also enhances the biocompatibility of EBCare for prolonged on-body use (Fig. 6b). During both indoor and outdoor nighttime tests, the EBCare device achieved a temperature drop of about 7 °C compared with radiative cooling materials and 10 °C compared to non-radiative cooling masks, outperforming regular fabric masks and those with a single cooling method. In addition to Ag particles, photocatalytic particles are widely employed for antimicrobial purposes. Cai et al. developed a layered composite nanofiber film made of thermoplastic polyurethane (TPU) and ZIF-8 nanoparticles [67]. The film exhibits impressive antibacterial properties under solar illumination due to active radicals generated by the photocatalytic properties of ZIF-8. The electrospun film combines a hierarchical structure of randomly stacked polymer fibers with scattering nanoparticles of ZIF-8, synergistically enhancing the sunlight reflectance of the film to 97%. Meanwhile, the intrinsic imidazole ring of ZIF-8 enhances infrared emittance to 93%. Aluminum-doped zinc oxide (AZO) nanomaterials possess strong oxidizing and photocatalytic properties, enabling them to achieve a sterilization effect. Li et al. incorporated AZO nanoparticles into PVDF nanofibers to form a large-scale and flexible passive daytime radiative cooling textile with high-efficiency antibacterial and UV-shielding properties [68]. These fabricated textiles exhibit a solar reflectance of 0.92 and a MIR emittance of 0.9. Moreover, for outdoor radiative coolers, fungi or mold growth can damage the materials and reduce cooling performance, making antimold protection essential for the long-term reliability of radiative coolers. Xin et al. developed a radiative cooling film by hot-pressing SiO_2_ particles and poplar catkins, forming a compact three-dimensional random network [69]. The multiscale scattering units ensure high solar reflectance, while cellulose contributes to mid-infrared emission via molecular vibrations. The antimicrobial property of the poplar catkin-derived film comes from the dense SiO_2_ layer, which prevents mold contact and inhibits its growth by blocking oxygen. After 35 days at 16 °C, the mold growth on the poplar catkin-derived film was much slower than on wood. Chen et al. prepared a cooling lignocellulosic material by growing SiO_2_ microspheres on the surface of delignified nanocellulose [70]. SiO_2_ microspheres not only enhance IR emittance due to their phonon polariton resonance but also inhibit combustion because of their excellent insulating properties. Importantly, this material exhibits outstanding resistance to mold growth. In a simulated outdoor environment (28 °C, soil, stump, water, and mold), lignocellulosic bulk and pure wood fiber bulk were tested for mildew resistance over 20 days. The lignocellulosic bulk showed almost no mold growth, while the pure wood fiber bulk was covered with mold.

Some classic inorganic nanoparticles, such as SiO_2_, TiO_2_, ZrO_2_, and Si_3_N_4_, generally have both MIR emission-enhancing and anti-environmental abilities. Li et al. reported a micro-sandwich-structured ultra-high-molecular-weight polyethylene (UHMWPE) film composed of layered polymer, pores, and SiO_2_ microspheres embedded within the pores [71]. The micro-sandwich pores, which exhibit the intense Mie scattering effect, are created by extracting liquid paraffin microdroplets and stabilized by the embedded SiO_2_ microspheres, enabling the film to reflect 99.1% of solar energy. The incorporation of SiO_2_ microspheres also provides desirable MIR emittance of 92.6% and enhances thermal stability. Additionally, the film demonstrates excellent resistance to UV exposure and acidic environments, attributed to its highly oriented molecular chains, high crystallinity, and high UV reflectance. Furthermore, the porous surface endows the film with effective anti-contamination performance against both dry dust and liquid pollutants. Zhang et al. embedded SiO_2_ microparticles into PTFE nanofibers to form a radiative cooling nanofiber membrane [72]. The membrane exhibits a MIR emittance of 95.8% and a solar reflectance of 95.4%. The surface protrusions on the nanofibers enhance the hydrophobic properties of the membrane. Additionally, the strong thermal and chemical stabilities of monoclinic ZrO_2_ microparticles impart corrosion and dissolution resistance to the inorganic dielectric particle-polymer composite [73]. To address the limitations of single-particle doping, dual-particle doping approaches are proposed. Cai et al. designed a cellulose composite film that incorporates TiO_2_@K_2_Ti_6_O_13_ (TiO_2_@PT) hybrid nanofillers as functional modifiers to enhance cooling performance and provide UV resistance [74]. TiO_2_@PT not only preserves the strong ultraviolet absorption capability of TiO_2_, but also further enhances the radiative cooling performance of the composite film through the high solar reflectivity and strong infrared emissivity of PT. The film reflects 97.6% of solar irradiance and exhibits a high infrared emittance of 95%. Through introducing SiO_2_ and Si_3_N_4_ nanoparticles, Yang et al. developed a bidirectional asymmetric film composed of a PE/SiO_2_/Si_3_N_4_ top layer for selective emission, a melt-blown polypropylene (MB-PP) middle layer for solar reflection and a PDMS bottom layer to broadband absorption of thermal radiation from the human body [75]. Thanks to this well-designed structure, the film can simultaneously reflect 94% of solar energy, emit 82% of thermal radiation toward outer space via the primary AW, and absorb 80% of thermal radiation from human bodies. Additionally, the film maintains its near-pristine spectral properties after accelerated aging and UV exposure tests.

Apart from particles, inorganic nanotubes and nanowires are commonly used to improve the properties of organic matrices. In an urban scenario, there is a large amount of thermal radiation from territory objects, such as the ground, buildings, and other infrastructure, which results in reduced cooling efficiency (Fig. 6c). To address this, Wu et al. designed an infrared spectrally selective hierarchical textile (SSHF) consisting of a polymethylpentene (PMP) fibrous layer, silver nanowires (AgNWs), and wool fabric (Fig. 6d) [51]. The surface PMP fibers, with a broad size distribution, enable efficient wideband scattering across the entire solar spectrum. Due to the presence of only C–C, –CH_2_, –CH, and –CH_3_ bonds, PMP shows high selective absorption in the primary AW range. Additionally, the bottom wool fabric absorbs thermal radiation from the skin, while the middle AgNW layer blocks MIR photons transmitted from the PMP layer and conducts heat. Consequently, the SSHF has a primary AW spectral selectivity ratio (the ratio of average emissivity in the primary AW to the average emissivity in the 2.5-8 and 13-20 μm ranges) of 2.23 and an average primary AW emittance of 0.85 (Fig. 6e). According to the theoretical calculations, the SSHF demonstrates higher cooling power than the broadband textile at any angle (Fig. 6f). Therefore, the SSHF can deliver significant cooling performance in urban areas, effectively countering the urban heat island effect. Beyond the excellent cooling performance, the SSHF demonstrates UV aging resistance, self-cleaning capabilities, and excellent mechanical performance (Fig. 6g). In addition, Li et al. prepared a film through coaxial electrospinning, with strontium barium titanate nanorods (BST NRs) as the core layer and TPU as the shell layer [76]. By utilizing the UV-blocking and free radical scavenging capabilities of BST NRs, the film exhibits outstanding resistance to UV exposure. Moreover, the incorporation of high refractive index BST NRs compensates for the reduction in reflectivity due to their UV absorption.

Numerous organisms thrive in extreme environments, evolving unique biological structures. These adaptations provide valuable insights and inspiration for the design of extreme environmental radiative coolers. Inspired by the brilliantly golden *longicorn beetles*, Zhang et al. developed a flexible hybrid photonic film for high-performing radiative cooling [19]. The bioinspired film consists of a micropyramid periodic array of PDMS, which encapsulates randomly distributed spherical Al_2_O_3_ particles, achieving high solar reflectance (95%) and excellent MIR emittance (96%) (Fig. 6h). Furthermore, the cooling film exhibits superhydrophobicity, resisting contamination attributed to the high surface roughness formed by the micropyramid structure. Inspired by spider silk, Yao et al. prepared a nanocomposite by wrapping the K_2_Ti_6_O_13_ nanofibers into polyethylene oxide (PEO) fibers (Fig. 6i) [66]. The nanocomposite exhibits a high thermal emission of 91% in the primary AW and a high solar reflectance of 94%. Since K_2_Ti_6_O_13_ can absorb high-energy UV photons and convert them into relatively harmless heat, it imparts excellent UV resistance to the film. As the duration of outdoor irradiation increased, the film preserved its high scattering structure of fiber connections and numerous pores, ensuring sustained high R_solar_. Inspired by the distinctive biostructure of Dictyophora, Zhou et al. developed an innovative radiative cooling film by combining hollow microparticles with a porous polymer [77]. By incorporating TiO_2_ microparticles, the film is effectively shielded from UV light. The rough surface and fluorine-containing branched chains impart excellent hydrophobic properties and dirt resistance to the film.

### Inorganic-Based Hybrid Material

Inorganic-based hybrid coatings, characterized by an inorganic phase as the dominant component, exhibit strong environmental resistance due to the inherent stability of inorganic materials. The high volume of inorganic fillers forms the backbone of the micro-nano structure, providing desirable solar reflectance and MIR emittance while enhancing protection for the organic binder and internal components in harsh conditions. The organic component mainly functions as a binder or cross-linking agent, maintaining structural integrity and supporting the overall optical performance. By varying the composition of inorganic fillers and organic binders, diverse structures can be designed to optimize both durability and thermal energy dissipation [78, 79].

**Fig. 7:**
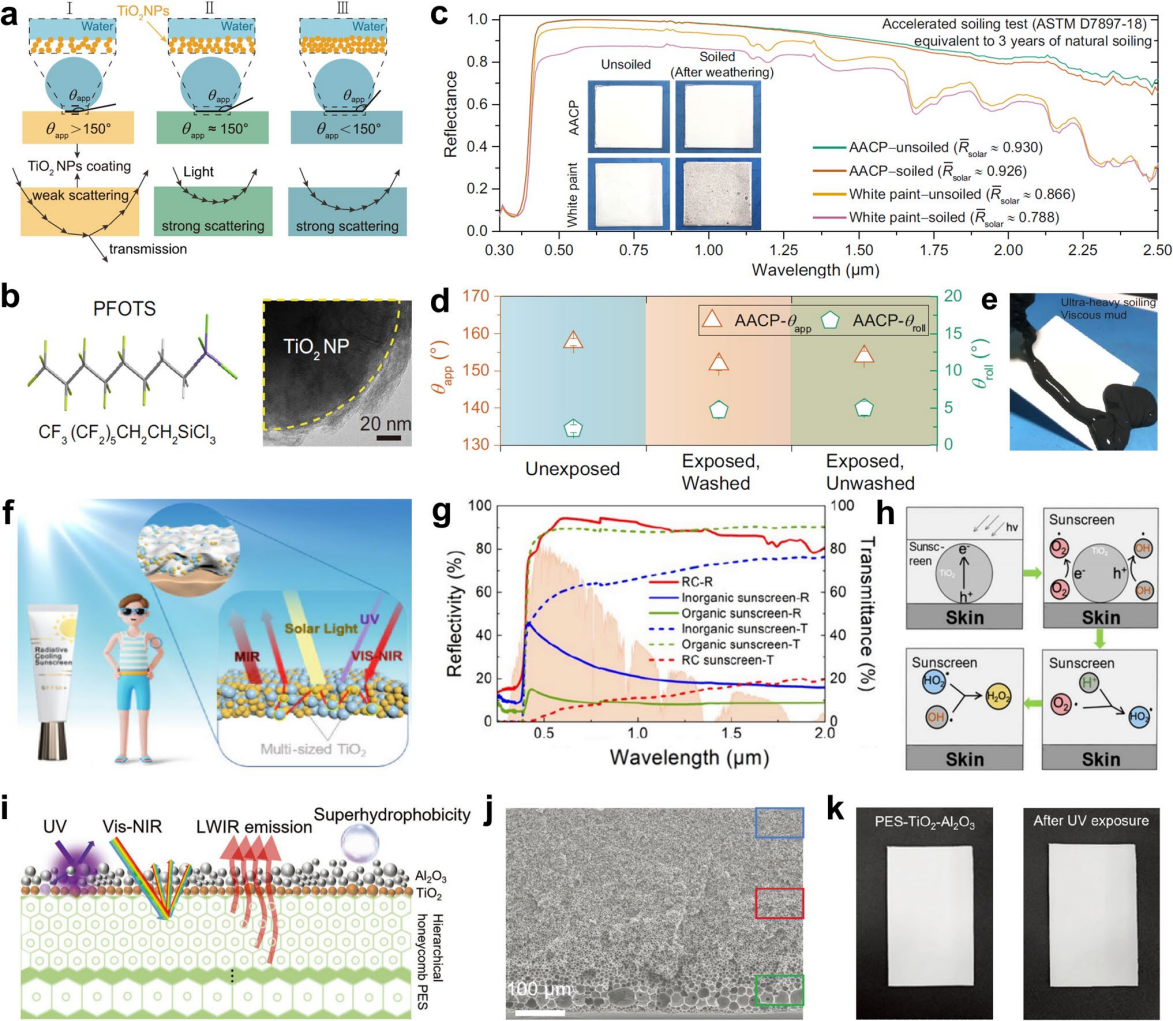
**a** Schematic of hydrophobic and scattering effects with varying *ϕ* and *f*. **b** Schematic of PFOTS structure (left), TEM image demonstrating the fluoride component grafted onto the TiO_2_ NP surface (right). **c** After the soiling test, the soiled ACCP coating maintained an almost unchanged solar reflectance. **d** Wetting behavior of the AACP after being exposed outdoors for 6 months. **e** Pollutants fall onto the ACCP coating and slide off, demonstrating outstanding self-cleaning function. **f** Conceptual design of radiative cooling sunscreen. **g** Solar reflectance of the radiative cooling sunscreen. **h** Mechanism of UV absorption by TiO_2_ nanoparticles. **i** Schematic of the three-layer PES-TiO_2_-Al_2_O_3_ cooler. **j** SEM image of the PES-TiO_2_-Al_2_O_3_ cooler. **k** Photograph of the PES-TiO_2_-Al_2_O_3_ cooler before and after UV exposure. **a-e** Reproduced with permission [80]. Copyright 2022, Springer Nature. **f–h** Reproduced with permission [81]. Copyright 2025, American Chemical Society. **i-k** Reproduced with permission [82]. Copyright 2024, Wiley–VCH

Song et al. demonstrated an anti-aging cooling paint (AACP)-based coating composed of hydrophobic rutile TiO_2_ nanoparticles [80]. As shown in Fig. 7a, the nanostructure with suitable packing density (*ϕ*) and liquid–solid contact fraction (*f*) can simultaneously achieve excellent radiative cooling and anti-soiling performance. To prepare the desired nanostructure, the nanoparticles (NPs) centered at 0.3 μm with a broad size distribution are selected to induce strong Mie scattering and enhance micro-/nano-scale roughness. Moreover, perfluorooctyltrichlorosilane (PFOTS) is grafted onto the surface of TiO_2_ NPs via silanization to introduce fluoride components, thus reducing the surface energy of the AACP coating (Fig. 7b). Thanks to the high refractive index of TiO_2_ and the strong scattering effect, the AACP possesses high reflectance (~ 0.93) in the solar radiation spectrum. Notably, the well-designed structure and grafting PFOTS allow the AACP to resist soiling for a prolonged period, making the coating maintain nearly unchanged radiative cooling efficiency (Fig. 7c). In the primary AW, the emittance of the AACP is enhanced by the collective stretching vibrations of C-F, C-C, Si-O, and Si-O-Si bonds in PFTOS. Moreover, the AACP can resist UV damage due to the absence of polymer binders. After outdoor aging, the AACP remained superhydrophobic with an apparent water contact angle of above 150° and a roll-off angle of above 5° (Fig. 7d). When contaminated with excessive pollutants, the contamination can quickly slide off the surface of the AACP, thereby achieving the self-cleaning effect (Fig. 7e). TiO_2_ can also be effectively used for the cooling and UV protection of skin, Xu et al. incorporated rutile TiO_2_ nanoparticles of various sizes into a commercial cream to achieve both high UV absorptance and high solar reflectance (Fig. 7f) [81]. TiO_2_ nanoparticles with a broad size distribution induce a strong Mie scattering effect, imparting the sunscreen with a high solar reflectance of 90.19% (Fig. 7g). Combined with a high MIR emittance of 92.9%, the sunscreen demonstrates exceptional temperature reduction of 2.3-6.1 °C for the human skin. Additionally, TiO_2_ particles with an appropriate bandgap can absorb UV energy through the generation and recombination of electrons and holes, thereby reducing UV-induced damage to the skin (Fig. 7h). When exposed to high-energy UV radiation, the radiative cooling sunscreen demonstrates an excellent UV-shielding effect, with a low transmissivity of 4.86% in the UV waveband. Apart from TiO_2_ particles, classic inorganic materials such as SiO_2_, Al_2_O_3_, and BaSO_4_, are widely utilized in extreme environmental applications [84. 85]. For instance, Li et al. proposed a radiative cooling coating formulated by combining alumina and sodium methylsilicate in a specific ratio [85]. The coating is fully inorganic, solvent-free, and features a simple fabrication process that only requires curing at room temperature after being applied to the substrate. At a thickness of 0.4 mm, the coating achieves a solar reflectance of 96.2% and an infrared emittance of 92.1%, corresponding to a theoretical cooling power of 109 W m^−2^. It also exhibits excellent resistance to UV aging and environmental degradation.

The integration of various inorganic materials leverages their diverse environmental resistance and radiative cooling capabilities, enabling superior long-term cooling applications in various extreme environments. Li et al. designed a composite structure with a polyethersulfone (PES) cooling layer at the bottom, a TiO_2_ nanoparticle layer in the middle, and an Al_2_O_3_ nanoparticle layer on top (Fig. 7i) [82]. The PES film features a highly porous structure with uniform microscale honeycomb pores distributed throughout, enabling strong scattering in the solar spectral range (Fig. 7j). The dual-particle cooler, composed of UV-reflective Al_2_O_3_ and UV-absorbing TiO_2_ layers, ensures strong UV protection and solar reflection. Moreover, Al_2_O_3_ nanoparticles can be pretreated to achieve superhydrophobicity, imparting the cooler with self-cleaning capability. Thus, the dual-particle cooler exhibits exceptional resistance to UV radiation and soiling (Fig. 7k). Additionally, structurally modified particles, such as TiO_2_@SiO_2_ core–shell nanospheres and ZnO@ZIF-8 core-shell materials, are widely integrated into organic binders to simultaneously enhance emittance in the primary AW, solar reflectance, and environmental durability [86, 87]. Liu et al. reported modified rutile TiO_2_@SiO_2_ nanospheres in commercial fluorocarbon resin matrix to achieve cooling [86]. The TiO_2_@SiO_2_-based coating achieves 93% solar reflectance and 94% emittance in the primary AW. The TiO_2_@SiO_2_ spheres are further modified with perfluorooctyltrimethoxysilane (PFS) to reduce the surface energy, thereby imparting anti-contamination and UV-tolerance properties. Kang et al. designed a polymer coating with polyhedral ZnO@ZIF-8 and methyl silicone resin [87]. The ZnO@ZIF-8/methyl silicone resin coating features polyhedral morphology and unoriented pores for enhancing light scattering in the solar spectrum and MIR emittance in the 8–13 μm range. Furthermore, the coating maintains optical performance after prolonged solar irradiation and thermal exposure, demonstrating excellent thermal stability.

### Inorganic Material

All-inorganic materials are considered ideal for radiative cooling in harsh environments due to their stable chemical structures. However, this stability also results in the high rigidity of inorganic molecular bonds, making it challenging to fabricate interconnected micro-nano structures that combine environmental tolerance with spectral manipulation. To address these challenges, various methods have been proposed to construct diverse all-inorganic nanostructures, including sintering, self-assembly, and electrospinning, enabling the development of advanced optical properties and enhanced environmental resistance [88–90].

**Fig. 8:**
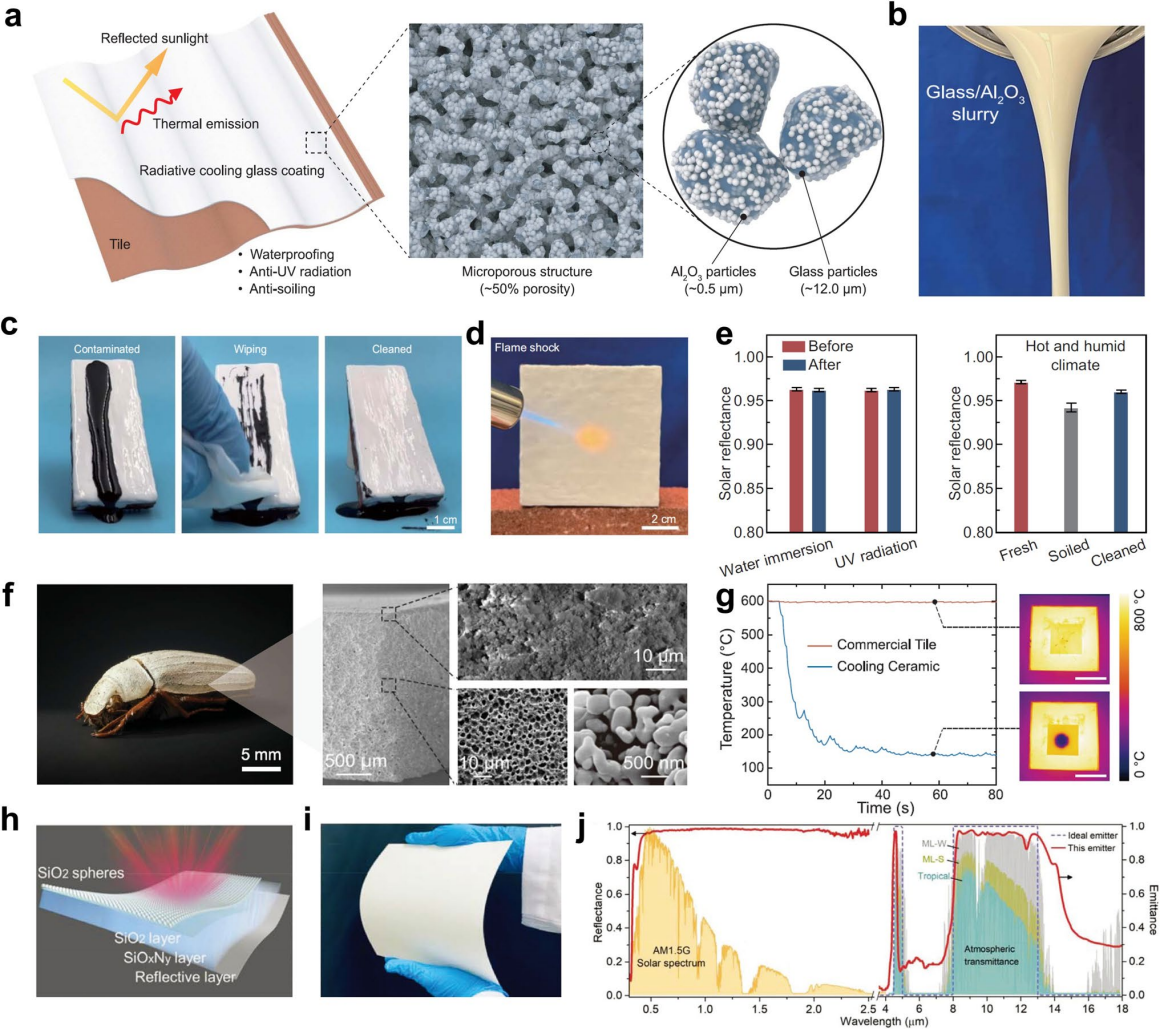
**a** Schematic of the radiative cooling glass coating. **b** Glass-Al_2_O_3_ particle slurry in ethanol exhibits excellent fluidity.** c** Radiative cooling glass with an enhanced protective layer exhibits excellent anti-pollution performance. **d** Cooling glass coating is subjected to flame shock at 1000 °C for about 10 s. **e** Solar reflectance of the radiative cooling glass coating before and after water immersion, UV exposure, and soiling tests simulating a hot and humid environment. **f** Photograph of the Cyphochilus specimen showing its white appearance (left). SEM images of the cooling ceramic sample with a hierarchical porous structure (right). **g** Changes in surface temperature as the samples are contacted by water droplets at 5-s intervals. **h** Schematic of the inorganic emitter. **i** Optical image of the inorganic emitter, which has a white appearance. **j** Spectral characteristics of the inorganic emitter in the solar and infrared bands. **a-e** Reproduced with permission [91]. Copyright 2023, AAAS. **f-g** Reproduced with permission [92]. Copyright 2023, AAAS. **h-j** Reproduced with permission [52]. Copyright 2022, Wiley–VCH

Zhao et al. designed a radiative cooling glass by incorporating glass and Al_2_O_3_ particles into a paintable slurry. Upon thermal annealing, a porous glass framework at the micron scale with Al_2_O_3_ nanoparticles is formed (Fig. 8a, b) [91]. Glass particles serve as a binder to form a robust porous framework, while high melting point α-Al_2_O_3_ particles act as anti-sintering agents, preventing full densification and promoting porosity. The cooling glass achieves over 96% solar reflectance and ~ 95% MIR emittance due to dual-scale high-bandgap scatterers and infrared-active glass particles. When coupled with a transparent protective layer, the glass also demonstrates strong environmental stability and anti-fouling performance (Fig. 8c). Moreover, the glass shows outstanding flame resistance, enduring 1000 °C flame shocks for ~ 10 s without changes in microstructure or optical reflectance. (Fig. 8d). The cooling glass also has the ability to maintain optical performance under various ambient conditions, encompassing UV exposure, water immersion, and soiling (Fig. 8e). Lin et al. drew inspiration from beetles and engineered alumina particles via phase inversion and sintering to fabricate a cooling ceramic (Fig. 8f) [92]. The phase inversion process yields a polymer-rich membrane that forms an anisotropic porous network carrying alumina particles. The subsequent sintering process facilitates the bonding of alumina particles, leading to the formation of a cooling ceramic with a well-preserved porous architecture. The cooling ceramic realizes 99.6% solar reflectance due to the strong scattering of the multi-dispersed pore system and 96.5% primary atmospheric emittance attributed to the vibrations of Al-O bonds. Moreover, the ceramic can withstand temperatures exceeding 800 °C and enables rapid cooling through the synergistic effects of radiative and evaporative cooling (Fig. 8g). Additionally, the ceramic can be designed to resist surface contamination by impregnation with organosilicon compounds, while its dense all-inorganic structure inherently provides excellent UV resistance.

Different from particle bonding under high-temperature conditions, Lin et al. reported an inorganic binder derived from perhydropolysilazane (PHPS) and developed a narrowband emitter consisting of a SiO_x_N_y_ layer positioned between a reflective substrate and a monolayer of SiO_2_ microspheres (Fig. 8h) [52]. The SiO_2_ microspheres were deposited onto the surface using an adapted Langmuir-Schaefer self-assembly method and adhered through a PHPS-derived binder, forming an inorganic emitter resembling white paper (Fig. 8i). The SiO_x_N_y_ layer exhibits inherent wavelength-selective emission in the 8-13 µm range, as the vibrational modes of Si-O and Si-N bonds fall within this range. Additionally, the monolayer of SiO_2_ microspheres further enhances infrared emittance through selective emission mechanisms. Consequently, the film achieves a high infrared-selective emittance of 94.6% while exhibiting a spectral selectivity ratio of 1.46 between the 8-13 µm and the entire waveband (Fig. 8j). Due to its all-inorganic hydrophobic structure, the emitter demonstrates exceptional resistance to UV exposure and water, ensuring prolonged durability. Banik et al. developed a dip-coated planar polymer emitter from polysilazane, with a 5 μm silicon oxycarbonitride coating that exhibits 0.86 emittance in the 8-13 μm range [93]. The structure, which incorporates a transparent polymer and a silver mirror, reflects 97% of solar irradiation and reduces heat absorption outside this range. Another classic inorganic material, MgO, is also a great candidate for extreme environmental radiative cooling. Wang et al. developed a gradient nanoporous MgO ceramic using dry-pressing molding and pressureless sintering [94]. The MgO-based ceramic achieves 96% solar reflectance and 95% selective emittance in the primary AW, aided by phonon polariton resonance and nanopore scattering. Owing to the Reststrahlen band of MgO in the 13-25 µm range, the ceramic achieves high spectral selectivity of 1.69. Furthermore, with the outstanding mechanical strength, environmental durability, and self-cleaning performance, it is well-suited for building space cooling applications.

Beyond particle-embedded and porous architectures, inorganic fiber structures provide an effective strategy for achieving flexibility and freestanding properties, rendering them highly adaptable to diverse surfaces. Tsai et al. fabricated scalable superhydrophobic silica metafibers (sh-SMF) through electrospinning and fluorosilane surface modification [95]. The ceramic sh-SMF shows ~ 90% emittance in the primary AW due to strong Si-O phonon resonances, along with thermal stability (> 1200 °C) and resistance to acid rain and UV. Fluorosilane modification further enhances its superhydrophobicity, imparting self-cleaning and anti-mildew performance. Apart from SiO_2_ fibers, Xin et al. fabricated a flexible alumina fiber membrane via electrospinning technology for radiative cooling applications [89]. The hierarchical fiber membrane structure synergistically combines the exceptional anti-aging performance, UV resistance, and high-temperature stability of inorganic materials with the intrinsic flexibility characteristic of fibrous structures. Tian et al. synthesized ultralong hydroxyapatite (HAP) fibers with high aspect ratios using a solvothermal method [90]. HAP fibers can self-assemble into bundles and interweave to form a porous network, which efficiently enhances solar reflectance through strong backscattering while absorbing MIR radiation via the molecular vibrations of phosphate radicals (PO_4_^3−^). Notably, HAP fibers can maintain their optical properties even at 800 °C, demonstrating exceptional thermal stability.

In summary, different materials in terrestrial dwelling environments correspond to various extreme conditions. Table 1 provides a clear comparison of the materials, their properties, and associated extreme environments.

**Table 1:** Summary of materials, their properties, and corresponding terrestrial dwelling extreme environments

Types	Materials	Solar reflectance (%)	AW emittance (%)	Properties	Environments	Refs
Organic material	POM nanotextile	~ 95	75.7	UV and abrasion resistance	UV-exposed and abrasion-prone environments	[54]
	Multilayer textiles (commercial silk /silk nanotextile/ PTFE)	96.5	97.1	UV resistance	UV-exposed environments	[57]
	MF cooling bulk	94	95	Fire resistance and self-extinguishing	Flame-exposed environments	[53]
	PDMS nanofibers	94	96	Thermosetting properties	High-temperature environments	[58]
	P(VDF-HFP) nanofibers	86.1	51.4	PM removal capability	PM-polluted environments	[56]
	CA film	97.4	92	Ice protection	Cold environments	[59]
	core–shell PVDF@PEI nanofibers	91.36	89.71	UV resistance, thermal stability, and hydrophobic properties	UV-exposed, high-temperature, and contaminated environments	[60]
	MMA/EDMA coating	97.6	98.3	Abrasion, corrosion, UV resistance, and superhydrophobic properties	Contaminated, abrasive, corrosive, and UV-exposed environments	[61]
	PTFE coating	94	93	UV resistance	UV-exposed environments	[62]
Organic-based hybrid material	PDMS: PDMS-b- polyethylene glycol (PEG)/Al_2_O_3_ with Agarose/Ag mask	95	95	Antimicrobial properties, io	Microbial environments	[65]
	TPU/ZIF-8 nanofiber film	97	93	Antibacterial properties	Bacterial environments	[67]
	PVDF/AZO textile	92.3	89.5	Antibacterial properties and UV resistance	Bacterial and UV-exposed environments	[68]
	SiO_2_/poplar catkins film	94.5	84.4	Mold resistance	Moldy environments	[69]
	SiO_2_/delignified nanocellulose	94	> 90	Waterproof, fireproof, and mold proof	Contaminated, flame-exposed, and moldy environments	[70]
	SiO_2_/polyethylene film	99.1	92.6	Acid resistance, UV resistance	Acidic and UV-exposed environments	[71]
	SiO_2_/PTFE nanofiber membrane	95.4	95.8	Hydrophobic properties	Contaminated environments	[72]
	P(VDF–HFP)/ZrO_2_ metamaterial	98	98.2	Thermal and chemical stabilities	High-temperature and corrosive environments	[73]
	CA/TiO_2_@PT film	97.6	95	UV resistance	UV-exposed environments	[74]
	PE/SiO_2_/Si_3_N_4_, MB-PP, PDMS film	94	82	UV resistance	UV-exposed environments	[75]
	PMP/AgNWs/wool textile	97	85	UV resistance and hydrophobic properties	UV-exposed and contaminated environments	[51]
	BST@TPU membrane	97.2	93.2	UV resistance	UV-exposed environments	[76]
	PDMS-Al_2_O_3_ film	~ 95	> 96	Superhydrophobic properties	Contaminated environments	[19]
	K_2_Ti_6_O_13_ nanofibers into PEO fibers	94	91	UV resistance	UV-exposed environments	[66]
	Fluorinated polyurethane (FPU) /TPU/TiO_2_	93.7	89.1	UV resistance, hydrophobic properties	UV-exposed and contaminated environments	[77]
Inorganic-based hybrid material	PFOTS/Rutile TiO_2_ coating	93	97	UV resistance, superhydrophobic properties	UV-exposed and contaminated environments	[80]
	Rutile TiO_2_/commercial cream	90.19	92.09	UV resistance	UV-exposed environments	[81]
	SiO_2_-coated glass bubble powder coating	96	98	Acid/alkali-resistant, UV-resistant, and hydrophobic properties	Acidic/alkaline, UV-exposed and contaminated environments	[83]
	BaSO_4_-acrylic paint	98.1	95	Abrasion resistance	Abrasive environment	[84]
	Alumina/sodium methylsilicate coating	96.2	92.1	UV resistance	UV-exposed environments	[85]
	PES/TiO_2_/Al_2_O_3_ film	98.1	92	UV resistance, superhydrophobic properties	UV-exposed and contaminated environments	[82]
	Rutile TiO_2_@SiO_2_ /fluorocarbon resin coating	93	~ 94	Superhydrophobic properties	Contaminated environments	[86]
	ZnO@ZIF-8/methyl silicone resin coating	~ 90	~ 95	Thermal stability	High-temperature environment	[87]
Inorganic material	Al_2_O_3_ particles/glass	> 96	95	Flame, UV, water, and soiling resistance	Flame-exposed, UV-exposed, and contaminated environments	[91]
	Cooling ceramic made of alumina particles	99.6	96.5	High temperature and UV resistance	High temperature and UV-exposed environments	[92]
	SiO_2_/SiO_x_N_y_/ Ag film	96.4	94.6	Hydrophobic properties, UV resistance	UV-exposed and contaminated environments	[52]
	Silver/polysilazane coating	97	86	Mechanical sturdiness, hydrophobicity, and chemical stability	Mechanical stress, contaminated and chemically corrosive environments	[93]
	Nanoporous MgO ceramic	96	95	Mechanical robustness, environmental resistance, and self-cleaning capability	Mechanical stress, environmental fluctuations, and contaminated environments	[94]
	Silica metafibers	~ 97	~ 90	High-temperature, acid rain and UV resistance, superhydrophobic properties	High-temperature, acid rain, UV-exposed, and contaminated environments	[95]
	alumina fiber membrane	> 95	> 87	UV and high-temperature resistance	UV-exposed and high-temperature environments	[89]
	Hydroxyapatite fibers	99	90	Thermal stability	High-temperature environments	[90]

## Terrestrial Extreme Environment

In addition to the extreme conditions encountered in terrestrial dwelling environments, there are diverse terrestrial extreme environments unsuitable for human habitation, particularly high-temperature or high-humidity regions such as deserts and tropical rainforests [96, 97]. In these environments, it is challenging to achieve efficient cooling management solely through radiative cooling via the primary atmospheric window. Therefore, it is necessary to incorporate additional heat exchange channels to enhance the overall cooling management performance [98–101]. In this section, we explore the enhancement of these additional heat exchange channels, encompassing thermal radiation through secondary atmospheric windows, thermal conduction and convection, evaporation, and phase change mechanisms, to improve cooling efficiency. Moreover, various micro-nano structures and materials are employed to precisely control the opening and closing of these additional heat exchange channels.

### Dual-Selective Emitter

Beyond the widely recognized primary AW in the 8-13 µm waveband, there are secondary AWs (3-5 and 16-25 µm ranges) allowing thermal radiation from terrestrial objects to escape into outer space. By utilizing these secondary AWs, the dual-selective emitter gains additional radiative dissipation channels and achieves a higher upper limit of cooling performance, making it well-suited to meet the growing cooling demands in extremely hot and dry environments [102]. In this section, we comprehensively discuss the design of dual-selective emitters in terms of material properties and structural configuration.

**Fig. 9:**
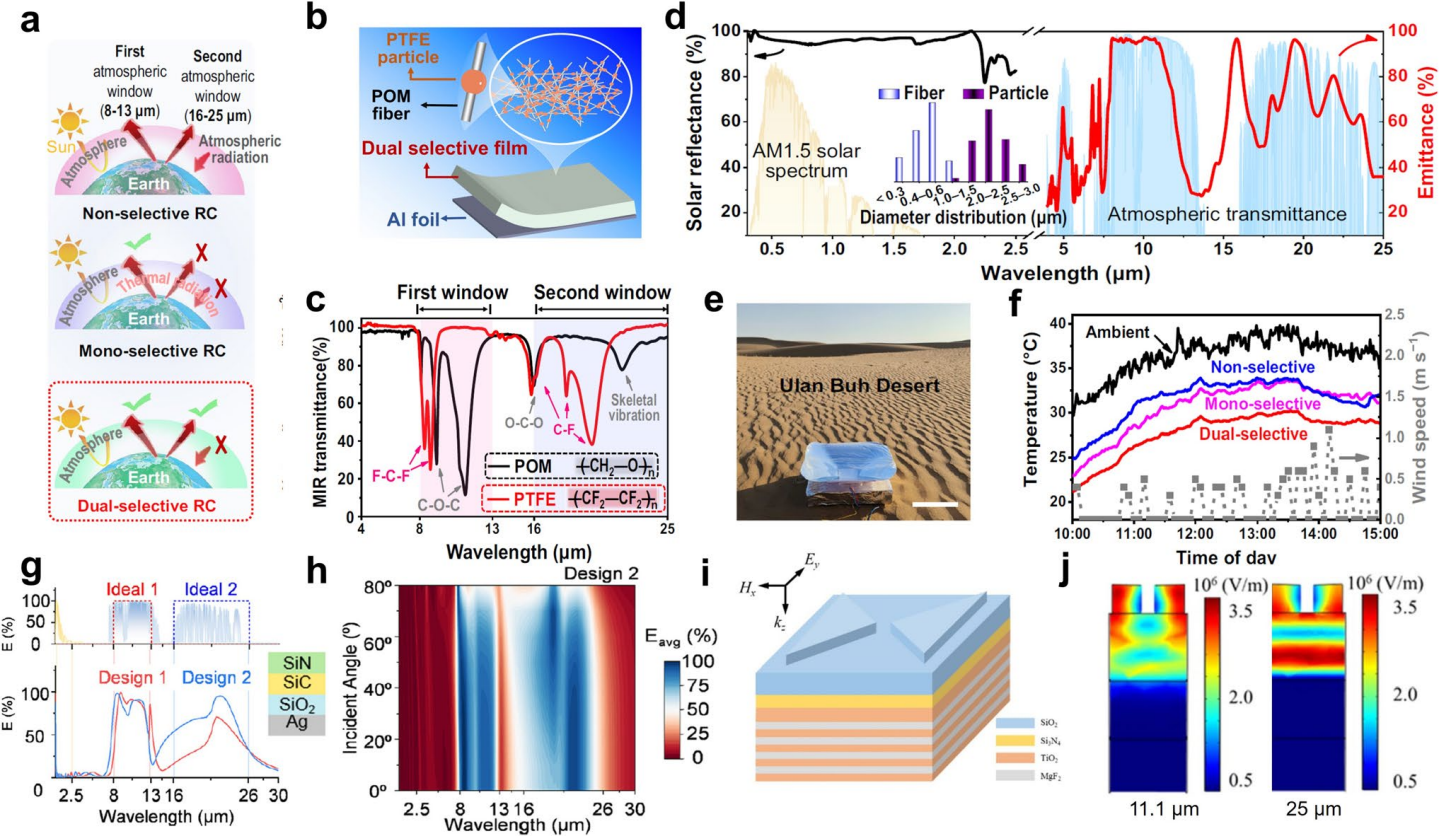
**a** Schematic of radiative heat exchange in three types of radiative coolers. **b** Schematic of the POM-PTFE film.** c** Molecular vibration of POM and PTFE in the MIR range.** d** Spectral characteristics of a POM-PTFE film on Al foil in the 0.3-25 μm range. **e** Photograph of the cooling test in the desert. **f** Temperature variations of the three emitters from 10 AM to 3 PM. **g** Emissivity spectrum of the designed coolers. **h** Contour plot showing the average emissivity of the designed cooler as a function of angle variation. **i** Three-dimensional schematic of an emitter featuring patterned SiO_2_ metamaterials on a multilayer. **j** Electric field distribution at the resonant positions (11.1 and 25 μm). **a-f** Reproduced with permission [28]. Copyright 2024, Springer Nature. **g-h** Reproduced with permission [103] Copyright 2021, Elsevier. **i-j** Reproduced with permission [104] Copyright 2021, IOP Publishing

By combining polymers characterized by molecular vibrations confined to multiple AWs, Wu et al. demonstrated a hierarchical POM-PTFE bead-like fibrous film deposited on an Al foil substrate, serving as an efficient dual-selective thermal emitter (Fig. 9b) [28]. The film emits thermal radiation exclusively via AWs, thereby maximizing cooling efficiency while minimizing the absorption of atmospheric inverse radiation (Fig. 9a). Specifically, POM exhibits strong molecular vibrations in the 8–13 μm range due to the absorption of C-O-C bonds, while PTFE demonstrates pronounced vibrational absorption in the 16-25 μm range attributed to C-F bonds (Fig. 9c). Notably, neither polymer exhibits significant absorption in the non-AW infrared range. Hence, the emitter exhibits an emittance of 83.2% in primary AW and 67.5% in 16-25 μm AW, while maintaining relatively high emittance in the 3-5 µm range. Moreover, it exhibits high reflectance in other MIR regions with low atmospheric transmittance, significantly reducing the impact of atmospheric inverse radiation (Fig. 9d). Besides, the random fiber arrangement and high reflectivity of Al foil result in a high solar reflectance (95.4%) in the solar radiation region, enabling the dual-selective emitter to possess outstanding cooling performance in deserts (Fig. 9e). During the peak heat and dryness of the day, the dual-selective emitter achieved the greatest subambient temperature drop of 7.9-9.5 °C, significantly outperforming the non-selective PVDF (4.2-5.8 °C) and mono-selective POM (4.8-6.8 °C) (Fig. 9f). Moreover, the dual-selective emitter shows a tensile strength of 8.2 MPa and exceptional UV resistance, remaining stable after 300 h of intense UV irradiation testing.

Apart from the inherent selective molecular vibrations of materials, ordered nanophotonic structures such as photonic crystals and metamaterials can enable highly selective radiative cooling through precise spectral manipulation. Mira et al. presented radiative coolers consisting of three dielectric layers (SiN, SiC, and SiO_2_) and an Ag back reflector (Fig. 9g) [103]. They employed a genetic algorithm to investigate dielectric materials and select the optimal thickness. The resulting design exhibits high emittance in the ranges of 8-13 and 16-25 μm, leading to exceptional cooling performance across various environments, particularly in arid conditions. Notably, the radiative cooler exhibits a highly angle-dependent emittance in the 16-25 μm AW, which is beneficial to reducing the absorption of ground emissions and improving the radiative cooling efficiency (Fig. 9h). Additionally, Yin et al. developed an emitter composed of triangular prism metamaterials deposited on a multilayer film for radiative cooling (Fig. 9i) [104]. The surface patterned structure consists of two triangular prisms on Si_3_N_4_ and TiO_2_ films, acting as the absorption layer. The SiO_2_ metamaterial layer controls the extended absorption band, with its geometry affecting the peak position. The bottom slit of the triangular prisms improves thermal emission in the 16-25 μm AW, while the Si_3_N_4_ layer enhances absorption in the 8-13 μm AW (Fig. 9j). Moreover, a nine-layer interlaced structure of TiO_2_ and MgF_2_ with optimized thicknesses serves as a reflector to prevent solar heating. As a result, the emitter exhibits high absorption in the 8–13 μm AW (98.56%) and 16–25 μm AW (96.31%), while solar band reflection exceeds 93%.

### Thermally Insulated Cooler

Under extremely high-temperature conditions, the significant temperature difference between the ambient environment and objects induces environmental parasitic heat flow, thereby compromising cooling efficiency. To mitigate this effect, thermally insulated radiative coolers are employed to obstruct the conduction and convection pathways [105]. Furthermore, the radiative cooling functionality is incorporated in thermal insulators to dissipate the accumulated heat, thereby reducing overall heat gain and enhancing the effectiveness of cooling management [106, 107]. Notably, due to the significant influence of environmental factors such as medium and flow rate on heat convection, controlling it remains a challenge. Therefore, thermally insulated coolers are typically designed with low thermal conductivity to minimize heat transfer from the surface to the interior.

**Fig. 10:**
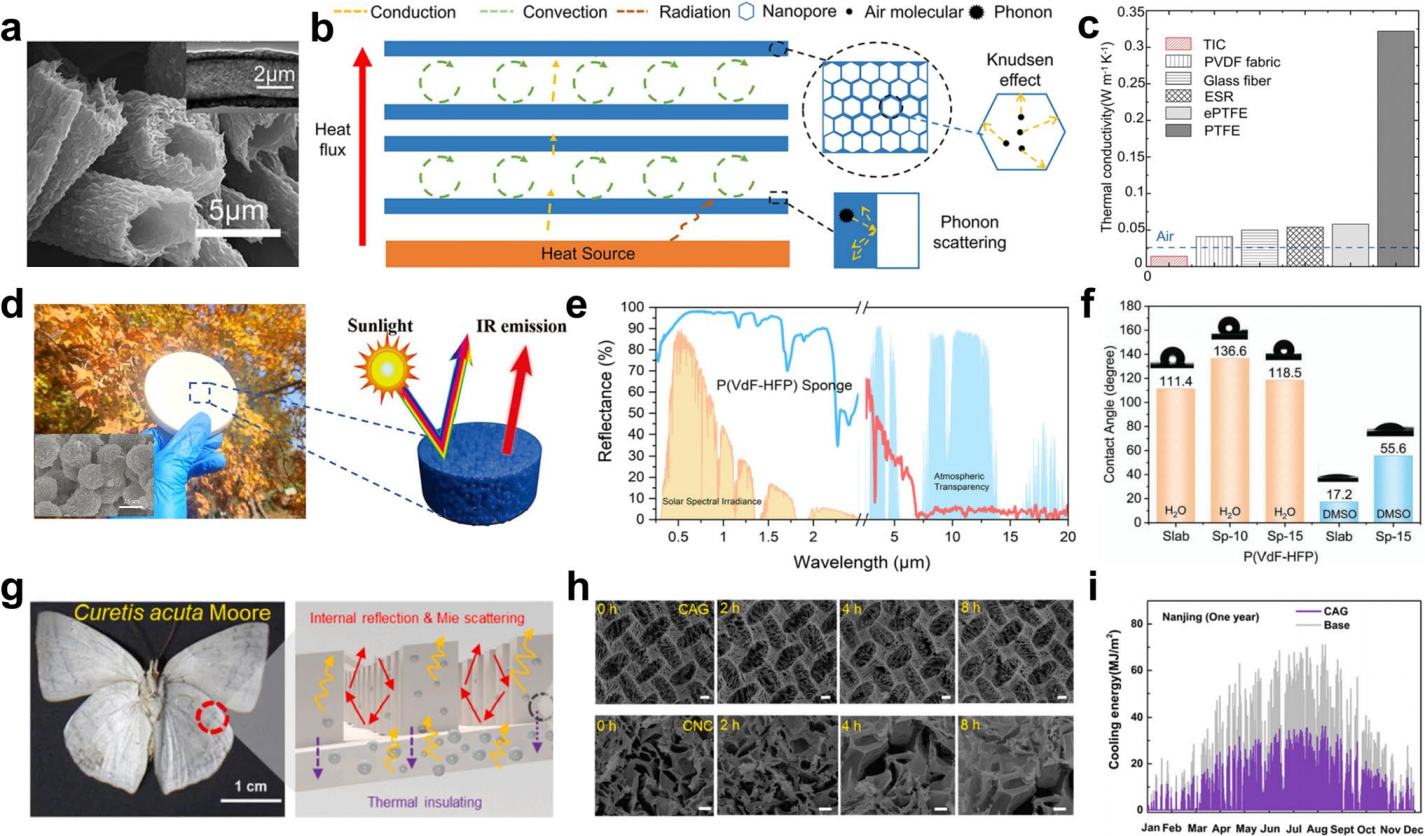
**a** SEM image of TIC with a hierarchical microfibrous structure. **b** Schematic of the heat exchange process of TIC. **c** Thermal conductivity of TIC (even lower than air) and the control samples. **d** Optical image of a sponge with the ability to reflect sunlight and emit infrared radiation. **e** Reflectance spectrum of the sponge. **f** Contact angle measurements of the sponges. **g** Bioinspired strategy and cooling mechanism schematic of the CAG. **h** Microstructure of CAG before and after UV exposure. **i** Simulated annual cooling energy in Nanjing using the CAG. **a-c** Reproduced with permission [108]. Copyright 2021, American Chemical Society. **d-f** Reproduced with permission [109]. Copyright 2024, Wiley–VCH. **g-i** Reproduced with permission [110]. Copyright 2023, Elsevier

Zhong et al. developed a thermal insulating cooler (TIC) composed of hierarchically hollow PVDF microfibers, which simultaneously enable radiative cooling and thermal insulation to mitigate external heat gain (Fig. 10a) [108]. As shown in Fig. 10b, hollow structures are crucial for thermal insulation because of the low thermal conductivity of air and the additional phonon scattering at solid–air interfaces, which further breaks heat transfer pathways through solids. The TIC demonstrates a thermal conductivity of 14 mW m^−1^ K^−1^ at room temperature, even lower than that of air (Fig. 10c). Notably, the TIC also exhibits superior radiative cooling performance, thanks to the combination of micro-nano scattering structures and the intrinsic MIR absorptivity of PVDF. Specifically, it achieves 94% solar reflectance and 94% MIR emittance, leading to a temperature drop of around 9 °C under 900 W m^−2^ sunlight. In addition to hollow fibers, the sponge structure is an ideal choice for thermal insulation benefiting from its abundant nanopores. Qin et al. introduced a vapor exchange method to fabricate particle-based P(VDF-HFP) sponges with an ultralow thermal conductivity of 48 mW m^−1^ K^−1^ (Fig. 10d) [109]. The P(VDF-HFP) sponge possesses a high MIR emittance of 95.6% because of the various thermal vibrational modes while reflecting 94.5% of solar radiation due to a strong Mie scattering effect stimulated by nanoparticles (Fig. 10e). Thus, the sponge can maintain a surface temperature of around 30 °C when exposed to a 40 °C surrounding. Beyond the impressive cooling properties, the sponge demonstrates outstanding mechanical strength and hydrophobicity, making it suitable for hot and humid environments (Fig. 10f). Zhou et al. reported a porous PDMS sponge through a facile template-casting method [111]. With the low thermal conductivity of 60 mW m^−1^ K^−1^, excellent solar reflectance of 93%, and high MIR emittance of 96%, the PDMS sponge achieves a cooling power of 43.17 W m^−2^ and a subambient temperature reduction of 4.6 °C under 1 sun illumination, meeting the stringent cooling management requirements in tropical areas.

Aerogel is characterized by high porosity and low density, making it one of the best thermal insulation materials. Drawing inspiration from the structural whiteness in butterflies, Cai et al. designed a cellulose nanocrystal aerogel grating (CAG) with tailored metasurfaces for effective cooling (Fig. 10g) [110]. The CAG exhibits low thermal conductivity (29 mW m^−1^ K^−1^) due to its high porosity. Moreover, the metasurfaces effectively scatter sunlight, granting the CAG an exceptional solar reflectance of 0.974. In the MIR region, the phonon polariton resonances of SiO_2_ nanoparticles and the bond vibrations of the cross-linked network synergistically improve the infrared emittance of CAG to 0.94. Furthermore, the UV reflection property and stable chemical bonds (Si-O-Si and Si-O-C) of CAG confer exceptional UV resistance. After 8 h of UV exposure, the CAG maintained its stable topological metastructures (Fig. 10h). As the CAG shows excellent and durable cooling performance, it significantly contributes to energy conservation in buildings, particularly during hot seasons (Fig. 10i). Han et al. prepared a radiative cooling coating by utilizing synthesized porous calcium silicate (CaSiO_3_) powder as the carrier for SiO_2_ aerogel [36]. The porous structure and wide bandgap of CaSiO_3_ enable strong solar reflection, while Si-O vibrations in SiO_2_ aerogel and CaSiO_3_ boost MIR emittance (8–13 μm). Additionally, SiO_2_ aerogel with intrinsically high porosity can lower thermal conductivity to 85.4 mW m^−1^ K^−1^. Combined with the radiative cooling functionality, the coating saves 70 kWh of electricity compared to commercial white coatings in hot climates. In addition to blocking the external heat input, internal heat dissipation can further improve cooling performance. Yang et al. developed a bilayer cooling film by placing a polyethylene (PE) aerogel layer on top of a commercial PDMS film [112]. The PE aerogel with 97.9% porosity efficiently reflects sunlight and provides insulation against external heat, with an impressively ultralow thermal conductivity of 32 mW m^−1^ K^−1^. The PDMS film can extract the heat from the substrate and dissipate it via thermal radiation. Moreover, the PE aerogel is transparent to thermal radiation, thereby preserving the unobstructed radiation channels. Benefiting from the synergy between asymmetric thermal conduction and radiative cooling, the film can realize the subambient temperature drop of 5–6 °C under solar irradiance intensity exceeding 1000 W m^−2^ in urban environments.

### Evaporative Synergistic Cooler

Evaporative cooling plays a critical role in boosting cooling performance in terrestrial extreme environments by utilizing the evaporative heat exchange channel. Through continuous water-vapor interactions with the surrounding air, evaporative synergistic cooling delivers substantial thermal regulation in scenarios where radiative cooling alone is insufficient, such as under high-temperature or high-humidity conditions [44, 113]. In this section, we discuss the design strategies of evaporative synergistic coolers, including systems based on hydrogels and hygroscopic particles.

**Fig. 11:**
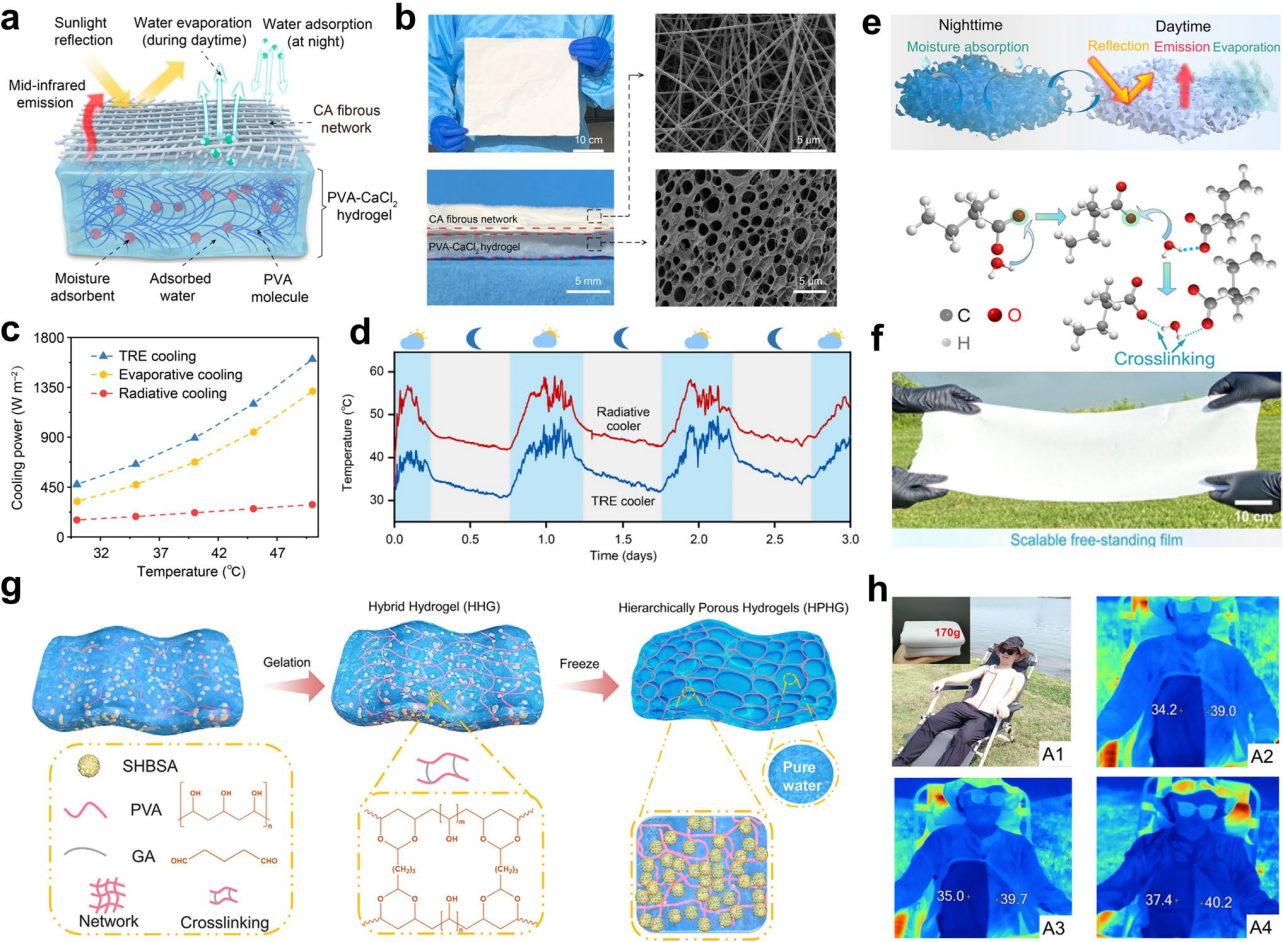
**a** Schematic of the tandem cooling design. **b** Photographs of the frontal and cross-sectional views for the tandem cooler (left). SEM images of the top CA fibrous network and the bottom PVA-CaCl_2_ hydrogel (right). **c** Calculated cooling power for the tandem, evaporative, and radiative cooling. **d** On cloudy days with a 200 W m^−2^ heat load, the tandem cooler consistently outperforms the radiative cooler in cooling performance. **e** Cooling mechanism of PAAS photonic hydrogel (top). Schematic of PAAS photonic hydrogel formation via hydrogel bond cross-linking (bottom). **f** Optical photograph of PAAS photonic hydrogel. **g** Schematic of HPHG synthesis and its chemical structures. **h** Cooling effect of HPHG in hot environments, including optical and infrared images. **a-d** Reproduced with permission [48]. Copyright 2022, AAAS. **e–f** Reproduced with permission [9]. Copyright 2023, Springer Nature. **g-h** Reproduced with permission [114]. Copyright 2024, Elsevier

Hydrogels are commonly used in evaporative cooling systems, featuring a layered structure that includes a top radiative cooling layer and a bottom hydrogel layer. This design effectively integrates radiative cooling with evaporative cooling, enhancing overall cooling performance. Li et al. proposed a tandem structure with a cellulose acetate (CA) nanofibrous network placed on top of a poly(vinyl alcohol) (PVA)-CaCl_2_ hydrogel layer (Fig. 11a) [48]. The top layer of the CA nanofibrous network effectively reflects sunlight, while molecular vibrations facilitate high MIR emission. Additionally, the PVA-CaCl_2_ hydrogel underlayer features numerous micropores, providing extensive pathways for moisture collection at night and water evaporation in the daytime (Fig. 11b). Based on the calculations, the tandem cooler outperforms both radiative cooler and evaporative cooler over a wide range of operating temperatures, particularly in high-temperature conditions (Fig. 11c). Moreover, under cloudy conditions, the tandem radiative cooler exhibits a lower temperature than the radiative coolers due to the beneficial effects of evaporative cooling (Fig. 11d). In addition, Hu et al. combined a PDMS top layer with a hydrogel bottom layer to prepare a radiative–evaporative bilayer cooler [115]. Benefiting from the combined advantages of two cooling mechanisms, the bilayer cooler outperforms single radiative or evaporative coolers. On hot sunny days, the bilayer cooler can generate a maximum cooling power of 424.4 W m^−2^. This value can increase to 650.6 W m^−2^ in cloudy weather.

Single-layer hydrogels can integrate evaporative cooling and radiative cooling within the same layer by utilizing intrinsic properties or incorporating dopants. Galib et al. prepared a photonic hydrogel to achieve hybrid radiative and evaporative cooling [9]. The hydrogel can be prepared by simply using atmospheric moisture to convert sodium polyacrylate (PAAS) powder into a film. In the presence of moisture, hydrogel bonds form between PAAS molecules, creating a robust cross-linked network (Fig. 11e). Therefore, the PAAS powder can transform into a large-area film with a white appearance and excellent mechanical performance (Fig. 11f). The PAAS hydrogel harvests atmospheric water at night and evaporates it during the day to boost cooling efficiency. Its porous structure reflects sunlight, while polymer chain vibrations enhance MIR emission. The hybrid cooling mechanism allows the PAAS hydrogel to achieve a temperature 7 °C lower than that of dry PAAS under 920 W m^−2^ solar radiation and partly cloudy weather. Hu et al. incorporated superhydrophobic silica aerogel (SHBSA) particles with micron and nanopores into a PVA matrix to fabricate a hierarchical porous hydrogel (HPHG) (Fig. 11g) [114]. The introduction of SHBSA and porous structures effectively increases multiple scattering, achieving approximately 90% reflectance in the solar radiation region. Moreover, the SHBSA possesses the capacity to enhance MIR absorption through the vibration of the Si–O–Si bond. The hierarchically porous structure enhances evaporation enthalpy, slows the evaporation rate, and further improves the durability of the HPHG. By combining efficient radiative and evaporative cooling, the HPHG generates an overall cooling power of 201 W m^−2^. As the HPHG with the water content of 80 wt% is lightweight and hydrophobic, it can be made into a cooling vest for personal thermal regulation at extremely high temperatures (Fig. 11h). Apart from silica aerogel, Fei et al. dispersed BaSO_4_ particles into a PVA hydrogel matrix to facilitate cooling performance [113]. In environments with high temperature above 33 °C, strong solar intensity around 1140 W m^−2^, and high humidity exceeding 60%, the cooler can achieve a temperature reduction of approximately 6 °C and generate an impressive cooling power of about 350 W m^−2^, which is 6–10 times greater than that of a radiative cooler in tropical climates. Furthermore, the cooler still maintained subambient temperatures even during rainy conditions.

In addition to hydrogels, other material systems can be employed to achieve evaporative cooling. Liu et al. introduced a hybrid passive cooling composite utilizing a metal–organic framework (MOF) [116]. The optically optimized MOF-801(Zr) block 450 nm light, enhancing visible (VIS) light scattering through small MOFs and abundant MOF–polymer interfaces. Moreover, the hierarchically porous structure further enhances solar scattering, resulting in a sunlight reflectance of 0.98. The functional groups of MOF-801 (Zr) raise infrared emissivity to 0.96, while its microporosity improves evaporative cooling efficiency. Consequently, the composite reduces temperatures by over 25 °C compared to natural cooling when applied to outdoor heavy-load power equipment. Fan et al. developed a metafabric with a hydrophobic styrene-b-(ethylene-co-butylene)-b-styrene (SEBS) nonwoven inner layer and a hydrophilic SEBS/PPO-PEO-PPO(F127)/Al_2_O_3_ outer layer, integrating Janus wettability with radiative and evaporative cooling. Its efficient moisture transport (0.31 g h⁻^1^ evaporation rate and 1220% transport index) enables up to 1 mL h⁻^1^ of sweat to evaporate for skin cooling [117]. Gu et al. developed a bilayer cooling textile with asymmetric wettability, made of banana cellulose aerogel and TPU nanofibers doped with ZnO nanoparticles. It has high solar reflectance (91.3% in 0.37–2.5 μm) and infrared emittance (90.2% in 8–13 μm) [118]. The textile also promotes directional sweat transport, boosting evaporative cooling and preventing discomfort.

Despite the excellent performance of evaporative synergistic coolers, their cooling efficiency remains insufficient in several terrestrial extreme environments. To address this issue, a viable approach is to integrate radiative cooling, evaporative cooling, and thermal insulation for enhanced cooling management. Inspired by camel fur, Wang et al. reported a physically foamed TPU porous elastic fiber and assembled the fibers into a fabric [119]. The novel fabric enables efficient unidirectional water transfer, producing an evaporative cooling effect on the body. With a thermal conductivity of 48 mW m^−1^ K^−1^, 98.7% solar reflectance, and 97.2% mid-infrared emittance, it generates a net cooling power of 300 W m^−2^ during daytime exposure to direct sunlight, with performance further improving at higher ambient temperatures. In addition, drawing inspiration from the cactus’s adaptation to dry and high-temperature conditions, Xu et al. designed a bilayer cooler consisting of a ZrO_2_/PTFE-polylactic acid (PLA) aerogel and a LiBr@PAAm hydrogel [120]. The aerogel blocks environmental parasitic heat while achieving radiative cooling due to the intrinsic molecular vibrations and porous structure. Besides, after adding ZrO_2_ and PTFE particle coatings, the cooling performance of the aerogel is further enhanced, exhibiting a solar reflectance of 0.95, an infrared emittance of 0.94, and an ultralow thermal conductivity of about 42 mW m^−1^ K^−1^. Moreover, the appropriate amount of LiBr enhances the hygroscopicity of the hydrogel, thereby improving its evaporative cooling capability. Compared to the hydrogel, the bilayer cooler exhibits a lower water evaporation rate, enabling sustained and efficient cooling management even in more extreme environments.

### Phase Change Synergistic Cooler

Unlike evaporative synergistic cooling, which relies on continuous water loss to drive cooling, PCMs function as thermal buffers, offering transient rather than sustained cooling. PCMs absorb heat during the solid-to-liquid phase transition process, effectively stabilizing the temperature of the protected object and delaying thermal rise. However, once the PCM has completely melted, its latent heat capacity is exhausted. Beyond this point, any additional heat input causes a temperature increase in the material [121]. To overcome this limitation, PCMs can be integrated into radiative cooling systems. During melting, the PCM absorbs part of the incoming heat, reducing the thermal burden on the radiative component. This synergistic interaction results in lower steady-state and peak surface temperatures than what could be achieved with radiative cooling alone [122]. In particular, the use of PCMs helps moderate the thermal profile of the system, reducing temperature fluctuations and improving overall thermal stability.

**Fig. 12:**
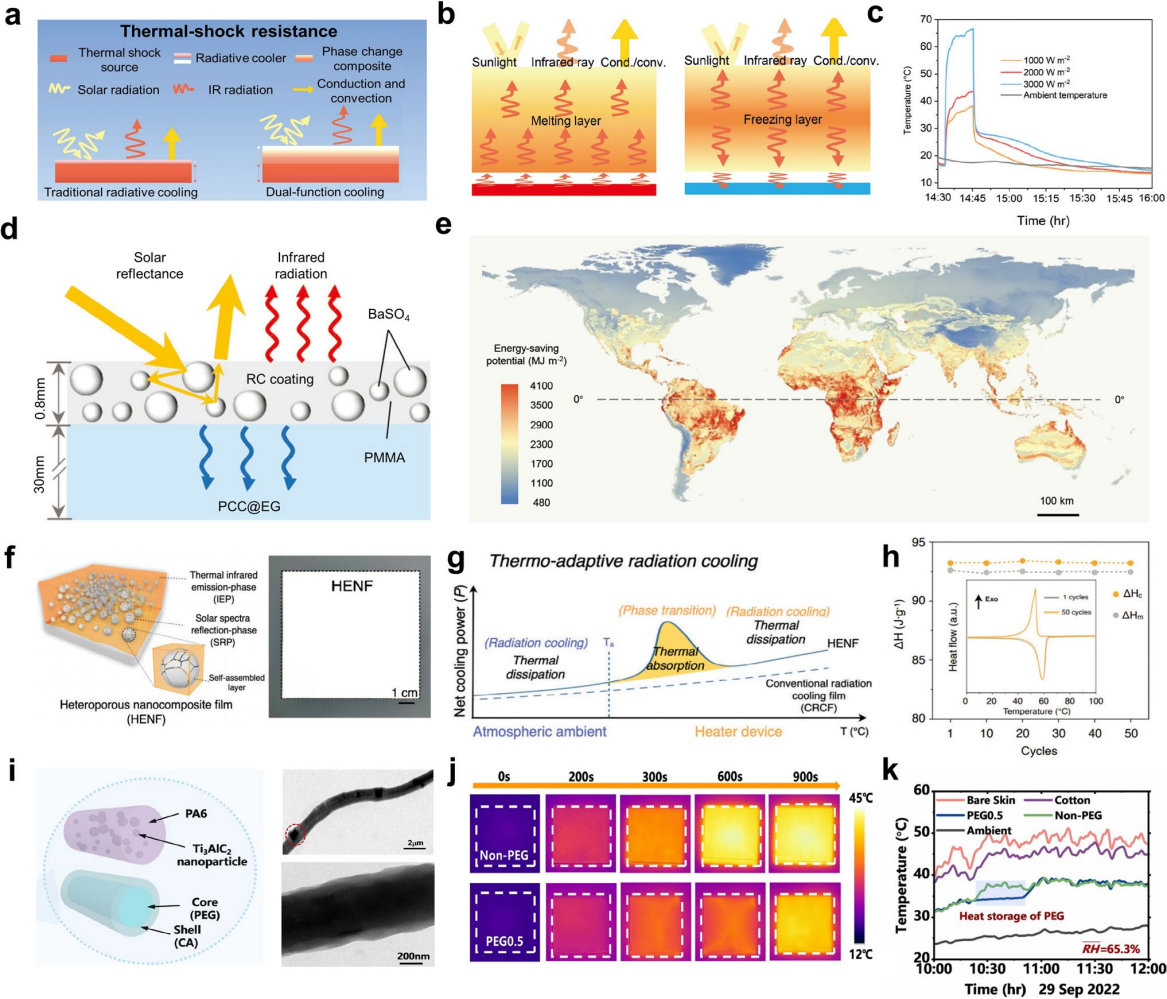
**a** Schematic of bifunctional cooling and conventional radiative cooling. **b** Heat transfer model for the melting and freezing processes of PCMs to enhance thermal shock resistance. **c** Temperature curves of coolers exposed to varying thermal shock power densities. **d** Schematic of the dual-functional film. **e** Yearly energy-saving potential of buildings across various terrestrial climate zones globally. **f** Schematic illustration of the structural design of the HENF. **g** Thermal adaptive radiative cooling characteristics of the HENF. **h** DSC curves and phase change enthalpy of the HENF sample after 50 heating and cooling cycles. **i** Schematic diagram and TEM image of the fibers. **j** Infrared thermal images of different textiles during the heating process. **k** Temperature data of skin, cotton, textile cooling layer, and ambient environment at 65.3% relative humidity. **a-c** Reproduced with permission [121]. Copyright 2024, Wiley–VCH. **d-e** Reproduced with permission [123]. Copyright 2024, Wiley–VCH. **f-h** Reproduced with permission [100]. Copyright 2024, Wiley–VCH. **i-k** Reproduced with permission [124]. Copyright 2024, Elsevier

Integrating a phase change layer into radiative cooling devices allows simultaneous radiative and phase change cooling (Fig. 12a). To prevent PCM leakage, it is common to use an organic or inorganic matrix to encapsulate the PCMs within the phase change layer. Qin et al. demonstrated a dual-function cooler assembled from P(VDF-HFP) film and a composite of expanded graphite (EG) encapsulating fatty alcohol (FA) [121]. Owing to the phase change mechanism of EG@FA, it can absorb heat through the melting process when thermal shock occurs, protecting objects from thermal accumulation and high-temperature damage. Once the thermal shock is removed, the stored heat is gradually released during the freezing process of EG@FA (Fig. 12b). Moreover, the cooler possesses great radiative cooling performance with solar reflectance of 95.9% and MIR emittance of 93.4%. Therefore, the dual-function cooler exhibits efficient thermal shock resistance, particularly in extreme environments subjected to high-power-density thermal shocks. Under a heat flux of 2000 W m^−2^, the dual-function cooler is capable of generating approximately 860 W m^−2^ of net cooling power and realizing a maximum temperature drop of 39 °C compared to a traditional radiative cooler. Even when the heat flux increases to 3000 W m^−2^, it can still rapidly reduce the temperature of objects (Fig. 12c). Wang et al. reported a composite film with both phase change heat absorption and radiative cooling functions [123]. They selected octadecane (C_18_H_38_) as an effective PCM and designed cross-linked networks using olefin block copolymer and SEBS additives to prevent leakage. The radiative cooling layer, positioned on top, consists of BaSO_4_ nanoparticles embedded in a polymethyl methacrylate (PMMA) matrix (Fig. 12d). The composite film exhibits a high phase change enthalpy of 138 kJ kg^−1^, a solar reflectance of 0.94, and an emittance of 0.96 in the primary AW. Thus, the energy device based on the bifunctional film exhibits significant yearly energy-saving potential for buildings in various terrestrial climate zones worldwide, particularly in near-equatorial regions characterized by high temperatures and humidity (Fig. 12e). Apart from the matrix packaging method, particle packaging is another feasible approach to protect PCMs. In this method, PCMs are enclosed within a thin solid shell to form core–shell microcapsules. Gu et al. fabricated a cooling membrane with a polyvinyl butyral (PVB) fiber layer on top and a PVB-PCMC layer on the bottom [125]. The PCMC is composed of a C_20_H_42_ core and a PMMA shell, providing a total latent heat of 124.3 J g^−1^ when the doping level reaches 70 wt%. Crucially, the latent heat of PCMC is only slightly decreased even after undergoing 100 heating-cooling cycles. At a high temperature of around 43 °C, the temperature of the PVB composite fabric remained consistently below the ambient temperature, closely approximating the human body temperature (around 35 °C).

Due to the additional interfacial contact resistance between the phase change layer and the radiative layer, the heat transfer path is inevitably extended, which, in turn, affects the phase change response speed and cooling efficiency. Additionally, the dual-layer structure also increases the volume and weight, making it difficult to meet the requirements of lightweight application scenarios. To overcome these limitations, researchers have developed doped single-layer structures that integrate both radiative cooling and phase change functionalities. In these designs, PCMs are encapsulated within microcapsules or matrices, similar to those in bilayer structures, to prevent leakage and maintain functionality. Tang et al. reported a cascaded heteroporous nanocomposite film (HENF), in which solar spectra reflection-phase microcavities are densely dispersed within the infrared emission-phase matrix. In this matrix, hexagonal boron nitride (h-BN) is assembled and embedded into the microcavities, forming a rough inner layer (Fig. 12f) [100]. Notably, a polydocosyl acrylate network with crystallization–melting properties is incorporated into the emission-phase matrix, thereby imparting the additional thermal absorption function based on radiative cooling (Fig. 12g). The HENF with phase transition properties enables the thermal absorption function to range from 45.7 to 63.2 °C, with a latent heat of 93.2 J g^−1^, which is suitable for extremely high-temperature environments. Importantly, after 50 heating-cooling cycles, it continues to exhibit stable phase transition behavior (Fig. 12h). Jiang et al. integrated thermal insulation and phase change properties into a radiative cooler to enhance its cooling performance [126]. They construct a porous PDMS@BN structure to reduce heat conduction while achieving the desired spectral properties. Octadecane, used as a phase change material, is loaded into the porous skeleton to compensate for performance loss under hot or humid conditions. The porous foam achieved a temperature drop of over 10 °C under an ambient temperature of 70 °C. Zhang et al. prepared a SiO_2_-PCMC/gelatin-hydroxyethyl cellulose composite aerogel [127]. The phase change microcapsule is composed of n-octadecane and a PMMA shell, providing a latent heat of 129.9 J g^−1^ for the enhancement of passive cooling. Moreover, the aerogel possesses great spectral properties with solar reflectance exceeding 92% and infrared emittance of over 95% as well as an ultralow thermal conductivity of 49.81 mW m^−1^ K^−1^. Thanks to the phase change, radiative cooling, and thermal insulation mechanisms, the aerogel maintains a low surface temperature of around 48 °C when heated by an 80 °C hot plate. Notably, the content of PCMs in single-layer structures needs to be carefully optimized to achieve a balance between spectral performance and latent heat storage capacity.

The core-shell fibrous structure is another effective way to encapsulate PCMs for achieving synergistic cooling. Yan et al. employed core-shell fibers with a CA shell and a polyethylene glycol (PEG) core to form the personal cooling textile (Fig. 12i) [124]. The micro-nano fiber networks and the formed pores enhance solar light scattering, while the intrinsic vibrations of CA and PEG contribute to high MIR emissivity. Furthermore, the solid-liquid phase transition of PEG facilitates efficient heat storage. This stored heat acts as an additional cooling source, effectively compensating for the reduced radiative cooling efficiency in hot and humid conditions. When placed on the heat stage, the textile without PEG heated linearly from 15.2 to 44.9 °C, while PEG-enhanced textiles maintained a temperature of about 34 °C for 430 s owing to the excellent heat storage capacity of PEG (Fig. 12j). Additionally, with the increase in relative humidity, the cooling effect induced by the heat storage of PEG is further improved. At a relative humidity of 65.3%, the PEG-enhanced textiles can provide an additional 3.9 °C temperature reduction compared with textiles without PEG (Fig. 12k). Zhu et al. employed coaxial electrospinning to develop a heat dissipation fibrous membrane by radiative and phase change cooling, in which n-octadecane acts as the core phase change material, while poly(3-hydroxybutyrate-co-3-hydroxyvalerate) (PHBV)/tetraethyl orthosilicate (TEOS) prepolymer forms the shell [128]. The n-octadecane in the core–shell structure demonstrates excellent cyclic thermal stability, enduring 50 consecutive phase change cycles while maintaining a consistent phase change temperature and latent heat. By combining the two cooling mechanisms, the fibrous membrane exhibits excellent cooling performance. Under an average solar radiation intensity of approximately 1000 W m^−2^ and an extremely high ambient temperature above 50 °C, the fiber membrane can realize a cooling power greater than 90 W m^−2^. However, core-shell fibrous structures have their own limitations. Due to geometric constraints, the encapsulation efficiency of PCMs in such micro-nano structures is inherently restricted, leading to a lower phase change enthalpy.

## Aeronautical Environment

Due to the infrared stealth requirements in aeronautical environments, radiative coolers must emit thermal radiation within the non-AWs rather than the AWs. Therefore, an ideal aeronautical radiative cooler should exhibit near-perfect emittance in the 2.5-3 and 5-8 μm ranges while maintaining high reflectance in the 8-13 μm range. To achieve this, nanophotonic structures, such as multilayer films and metamaterials capable of inducing photonic crystal effects or electromagnetic resonances, are designed to precisely manipulate the MIR spectrum [129, 130]. Additionally, thermal insulation materials are integrated to further lower the surface temperature of aeronautical devices, thereby reducing infrared signal intensity and enhancing camouflage performance [131].

### Multilayer Structure

Multilayer structures are commonly utilized to manipulate the ultrabroad spectrum, spanning from the visible to the infrared range, to realize multispectral camouflage and cooling effects. This unique architecture satisfies the different camouflage requirements of both the infrared and visible ranges, while also promoting effective radiative heat dissipation in the non-AWs [132]. Importantly, the integration of stacked layers has the capacity to further broaden the ranges of spectral manipulation, enabling compatibility with radar and laser stealth [133].

**Fig. 13:**
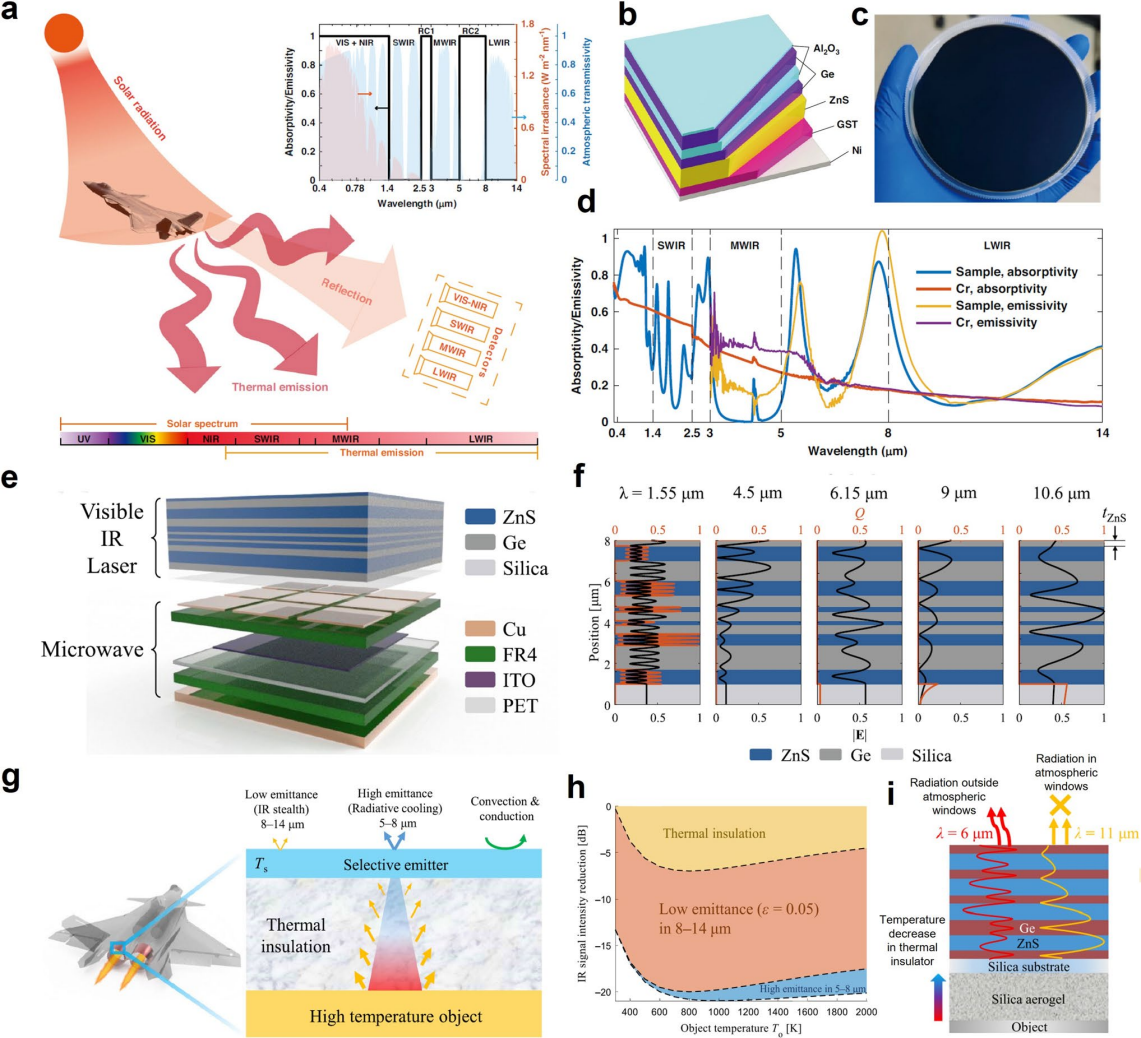
**a** Schematic of the conventional visible-to-infrared detection bands, signal sources, and spectra of an ideal cooler for aeronautical environments. **b** Schematic of multilayer structure for camouflage. **c** Designed multilayer structure deposited on the Si substrate. **d** Spectrum of the sample and Cr reference. **e** Schematic of the multispectral camouflage film. **f** Electric field and resistive loss in the ZnS/Ge emitter at different wavebands.** g** Schematic of the selective emitter combined with a thermally insulating aerogel for high-temperature infrared stealth. **h** Contribution to infrared signal reduction from thermal insulation (orange), low emittance in 8–14 μm AW (red), and high emittance in the 5–8 μm non-AW (blue) at various object temperatures. **i** Structure design of the insulated selective emitter and normalized electric field intensity at 6 and 11 μm. **a-d** Reproduced with permission [130]. Copyright 2023, Springer Nature. **e-f** Reproduced with permission [133]. Copyright 2021, Springer Nature. **g-i** Reproduced with permission [40]. Copyright 2020, Springer Nature

Qin et al. developed a multilayer structure with excellent visible and infrared camouflage, as well as radiative cooling performance. This structure exhibits high emittance in 2.5-3 and 5-8 μm regions, low emittance in other MIR wavelengths, and low reflectance in the VIS and near-infrared (NIR) spectra (Fig. 13a) [130]. To prepare it, they stacked two Al_2_O_3_/Ge layers of different thicknesses, a ZnS layer, a GST layer, and a Ni layer (Fig. 13b). In the VIS waveband, the intrinsic absorption of the Ge layer and the Al_2_O_3_ anti-reflection layer leads to a high absorption (Fig. 13c). In the NIR range, the GST layer transitions into a lossy medium, leading to the absorption of incident photons in this region. In the two radiative cooling wavebands (2.5-3 and 5-8 μm ranges), light is partially absorbed by the GST layer and completely absorbed by the next Ni layer. As a result, the sample exhibits a high average emittance of 74.2% in the 2.5-3 μm waveband and 47.3% in the 5-8 μm waveband. In other infrared ranges, both the electric field and resistive loss are suppressed, leading to low emittance in these ranges (Fig. 13d). Peng et al. developed a four-layer film with alternating Ag and Ge to achieve efficient radiative cooling and infrared stealth [134]. Through the intrinsic thermal radiation of the ultrathin Ag film and the impedance matching of two Ge films, the multilayer film displays selective emittance of 82% in the 5-8 µm range. Additionally, other infrared photons are reflected by the bottom Ag substrate, causing the low emittance of 18% and 31% in the 3-5 and 8-14 µm AW, respectively. Zhang et al. designed a Ge/ZnS 1D heterostructure photonic crystal using a genetic algorithm [135]. The optimal design achieves a low emittance of 4.6% and 19% in the ranges of 3-5 and 8-14 µm, respectively. Meanwhile, the crystal can dissipate heat in the 5-8 µm region due to the high emittance of over 57%.

Apart from modulation in the infrared and visible bands, many aeronautical coolers are able to manipulate spectra across different wavelengths to achieve multi-wavelength camouflage in various scenarios. Zhu et al. fabricated a multilayer structure to meet the multiple spectral requirements [133]. The structure is composed of a Ge/ZnS multilayer emitter for VIS/laser/MIR camouflage and radiative cooling, along with a Cu-ITO-Cu metasurface for microwave absorption (Fig. 13e). The alternating Ge and ZnS layers form a 1D photonic crystal with bandgaps in the 3-5 and 8-14 µm AWs, resulting in reflectance values of 0.89 and 0.88, respectively (Fig. 13f). Notably, the narrow band low reflectance at the laser detection wavelength of 10.6 µm is achieved with the 5th–7th Ge/ZnS layers, as the photonic bandgaps are disrupted by these three layers. At another laser detection wavelength of 1.55 µm, the photons are absorbed by the Ge layers due to intrinsic loss. In the heat dissipation region (5-8 µm), the light can transmit the photonic crystal and be absorbed by the lossy silica substrate, resulting in a high absorptance of 0.61. For the microwave X-band, the combined structure can achieve a return loss below − 8.25 dB. In addition, this group designed a device with thermal insulation, radiative cooling, and IR camouflage properties by combining silica aerogel and a Ge/ZnS multilayer (Fig. 13g) [40]. According to the theoretical calculations, the infrared signal intensity can be significantly reduced through the manipulation of the thermal conduction and convection channel, the atmospheric channel, and the non-atmospheric channel (Fig. 13h). By optimizing the thicknesses of the multilayer structure, the selective emitter can reflect about 95% of infrared radiation in the 8-14 µm band and emit approximately 58% of thermal radiation in the 5–8 µm band (Fig. 13i). When heated by a high-temperature object at 600 °C, the selective emitter can achieve a radiation temperature of 11.7 °C lower than that of highly reflective stainless steel. Jiang et al. designed a multilayer structure and optimized the structural parameters depending on a genetic algorithm [136]. The structure comprises seven layers made from five materials (SiO_2_, Ge, ZnS, Pt, and Au), with a total thickness of 3.735 µm. Owing to the asymmetric Fabry–Perot (F-P) resonance and the absorption of Pt, the multilayer structure possesses high emittance at laser-detected wavelengths and in the non-AW, while reflecting MIR light in the 3-5 μm and 8-14 μm ranges via the metal layer.

### Metamaterial

Metamaterials and their 2D counterpart, metasurfaces, possess the ability to manipulate multiple wavelengths [137, 138]. Through employing well-designed metal patterns and dielectrics, various electromagnetic resonances, such as surface plasmonic resonance, magnetic resonance, and F-P resonance, can be induced to achieve high emittance in the 5-8 µm non-AW and low emittance in the 3-5 and 8-13 µm AWs. Remarkably, by combining nanostructures of different scales, metamaterials and metasurfaces can be endowed with radar and laser stealth, making them well-suited for aeronautical environments [139, 140].

**Fig. 14:**
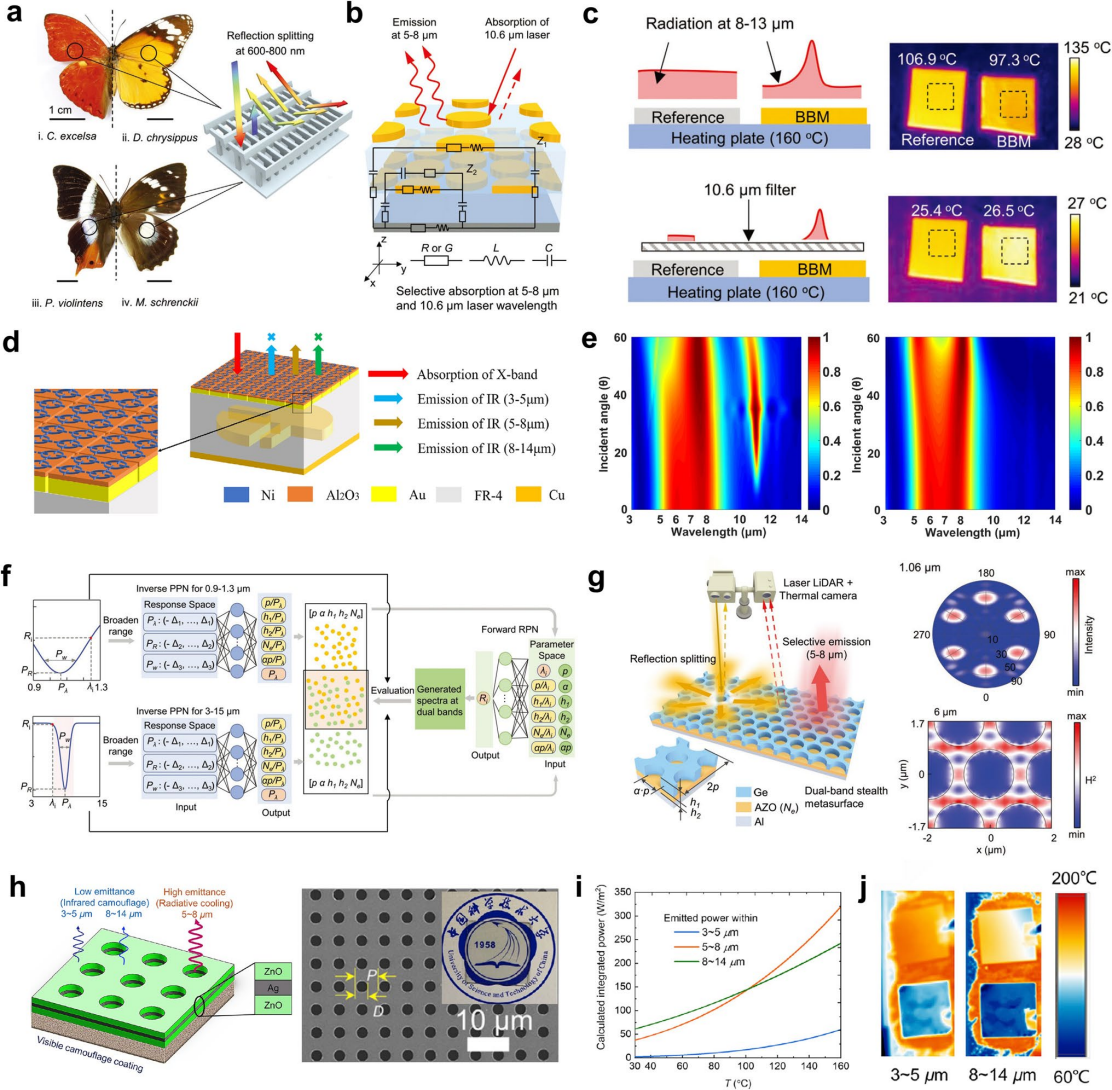
**a** Four butterflies show a reflection splitting effect in the 0.6–0.8 μm range, which can inspire low specular reflectance for NIR detection lasers. **b** Schematic of the work principle of BBM, demonstrating the emission peaks at 5–8 μm and 10.6 μm.** c** Measurement diagram and infrared images of the reference and BBM. **d** Schematic of the structure of HMM. **e** Emittance of FSE at varied incident angles (TM and TE modes, left and right, respectively). **f** Schematic of the ML-enabled inverse design for optimizing structural parameters. **g** Schematic of metasurface for dual-wavelength camouflage (left). Simulated backscattering intensity at 1.06 μm and magnetic field intensity distribution at 6 μm (right). **h** Schematic of the structure of photonic crystal (left). SEM and photograph of photonic crystal (right). **i** Emitted powers by the sample in the 3–5, 5-8, and 8-14 μm ranges at varied temperatures. **j** Radiation temperature of the photonic crystal and bare silica captured by infrared cameras in the ranges of 3–5 and 8–14 μm. **a-c** Reproduced with permission [141]. **d-e** Reproduced with permission [142]. Copyright 2022, Wiley-VCH. Copyright 2023, Wiley-VCH. **f-g** Reproduced with permission [143]. Copyright 2023, Wiley-VCH. **h-j** Reproduced with permission [144]. Copyright 2021, Elsevier

Inspired by four butterfly species (*Cymothoe excelsa, Danaus chrysippus, Palla violintens,* and *Mimathyma schrenckii*), which exhibit broadband reflection splitting, Liu et al. developed a bilevel bioinspired metamaterial (BBM) that combines visible manipulation, laser stealth, and MIR selective radiation (Fig. 14a) [141]. The BBM is composed of dual-deck Pt disks, a SiO_2_ intermediate layer, and a bottom Al reflector (Fig. 14b). The BBM exhibits large scattering angles and excellent ultralow specular reflectance (8.2%) across the 0.8-1.6 μm range due to the enhanced reflection splitting effect of the biological microstructure, which effectively reduces multiple laser echo signals. By modulating the thickness of the SiO_2_ middle layers and the period of Pt disks in the lower deck, diffraction and film interference interactions generate intriguing structural colors. Moreover, dual magnetic polaritons are induced by the interaction between the Pt disks and the bottom Al reflective substrate, leading to high absorption in the non-AW (5–8 µm) and at the laser detection wavelength (10.6 µm). Compared to the reference, which has an average absorptance of around 40% at 8-13 µm, the BBM exhibits a 9.6 °C lower apparent temperature, as measured by the thermal imager, indicating reduced infrared signal intensity in the 8–13 µm waveband. Additionally, the BBM displays a higher apparent temperature upon the addition of a 10.6 µm filter, further demonstrating an intense absorption at this wavelength (Fig. 14c). Similar to embedded metal patterns, Qin et al. prepared a hierarchical metamaterial (HMM) by integrating a frequency-selective emitter (FSE) with a microwave absorber (MA) (Fig. 14d) [142]. The MA layer consists of Cu patterns embedded in an FR-4 spacer, along with a Cu ground plane. The FSE layer comprises periodically arranged metal-dielectric-metal metasurfaces. The supercell of the metasurface is a rudder-shaped nickel pattern composed of a ring and four rods symmetrically nested on it. The FSE layer exhibits outstanding high-absorptivity and wide-angle performance for both TM (Transverse Magnetic) and TE (Transverse Electric) polarization modes by coupling propagating surface plasmonic resonance and localized surface plasmonic resonance (Fig. 14e). Overall, the HMM shows low average surface emittance in the range of 3-5 μm (0.25) and 8-14 μm (0.26), while exhibiting higher average surface emittance in the non-AW of 5-8 μm (0.86).

Ordered pore array structures can also be utilized to precisely manipulate the MIR spectrum. Liu et al. developed a metasurface compatible with laser and infrared stealth, as well as efficient radiative cooling, through a machine learning-enabled (ML-enabled) inverse design method (Fig. 14f) [143]. The finally generated metasurface features a Ge hole array on top and an Al film at the bottom, with an AZO layer separating them (Fig. 14g). At a laser detection wavelength of 1.06 µm, the metasurface achieves a specular reflectance of less than 10% due to the reflection splitting effect which deflects the reflection into high angles. In the MIR range, the surface-plasmon polariton is stimulated at the interface of the Ge and AZO layers, leading to a broad absorption peak in the range of 5–8 µm, with a maximum above 94% and a full width at half maximum exceeding 1.8 µm. Dang et al. introduced a multi-functional device by combining three functional modules (Fig. 14h) [144]. The top module, a 2D photonic crystal on a silicon substrate (ZnO/Ag/ZnO/SiO_2_), manipulates visible and MIR light. The module shows low emittance in the detected range (3-5 and 8–14 μm) due to the intrinsic reflection of silver. A 2D periodic aperture array induces extraordinary optical transmission (EOT), allowing MIR light within 5–8 μm to penetrate the SiO_2_ layer and be absorbed through phonon polariton resonance. Additionally, visible transmittance is enhanced by large apertures and F-P resonance in the remaining continuous ZnO/Ag/ZnO structure. The visible camouflage module provides optical concealment, and the bottom thermal insulator reduces heat conduction from high-temperature targets. The device generates considerable radiative cooling power in the region of 5–8 μm, with the emitted power contribution through this region increasing significantly as the temperature rises, indicating the ability of infrared camouflage under high-temperature conditions (Fig. 14i). When tested with an infrared camera, the device exhibits a lower radiation temperature and reduces infrared signal intensity in the detected range (Fig. 14j).

Zhou et al. employed multilayer structures to design a metasurface for wavelength-selective absorption [145]. The metasurface comprises two metal–dielectric–metal resonators on a flexible polyimide substrate. The top Ag film and the bottom Ag reflective layer are employed to suppress the thermal radiation in the AWs (3–5 and 8–14 μm). Additionally, the Ag/MgF_2_/Ag/MgF_2_ disk metasurface has the ability to excite magnetic resonance in the 5–8 μm range, enabling absorption of thermal radiation in the non-AW. Deng et al. proposed a wavelength-selective scattering metamaterial (WSSM) with a chess-like configuration containing two-unit cells [146]. Unit cell I and unit cell II both consist of two Bragg reflectors (BR) with Ge/ZnS multilayer structures and a ZnS top layer with different thicknesses. The well-designed BR_1_ and BR_2_ can form forbidden bands to achieve high reflectance in the regions of 3-5 and 8-14 μm. For the 5-8 µm band, the low reflectance originates from the destructive interference between the two structures, allowing most of the energy to be absorbed by the substrate. In addition, the WSSM can achieve reflection splitting at 8-12 μm and thin-film destructive interference effect at 1.06 and 1.55 µm, causing exceptional laser stealth performance.

## Space Environment

Compared to conventional terrestrial and aeronautical environments, space environments are characterized by their vacuum conditions. In space environments, the cooling surface must endure harsh cosmic conditions, including stronger solar intensity, UV radiation, cosmic rays, and atomic oxygen exposure [147–149]. Intensive solar irradiation can cause significant temperature rises, leading to material fatigue and thermal stress. UV radiation, meanwhile, degrades micro-nano-structured surfaces, resulting in embrittlement, discoloration, and optical performance loss. Notably, UV radiation can decompose molecular oxygen into atomic oxygen, a highly reactive species that aggressively oxidizes and erodes exposed materials. Cosmic rays, comprising high-energy electrons, heavy ions, and protons, possess enough energy to break chemical bonds, accelerating material aging and compromising structural integrity [150, 151]. On the other hand, the absence of atmosphere allows the cooling surface to fully utilize the whole thermal radiation waveband. As a result, middle infrared heat dissipation channels with wavelengths greater than 2.5 μm are fully opened for space cooling devices [152, 153]. Lastly, the spacecraft surface should be endowed with thermal insulation properties to maintain a stable and suitable temperature inside the spacecraft [154].

**Fig. 15:**
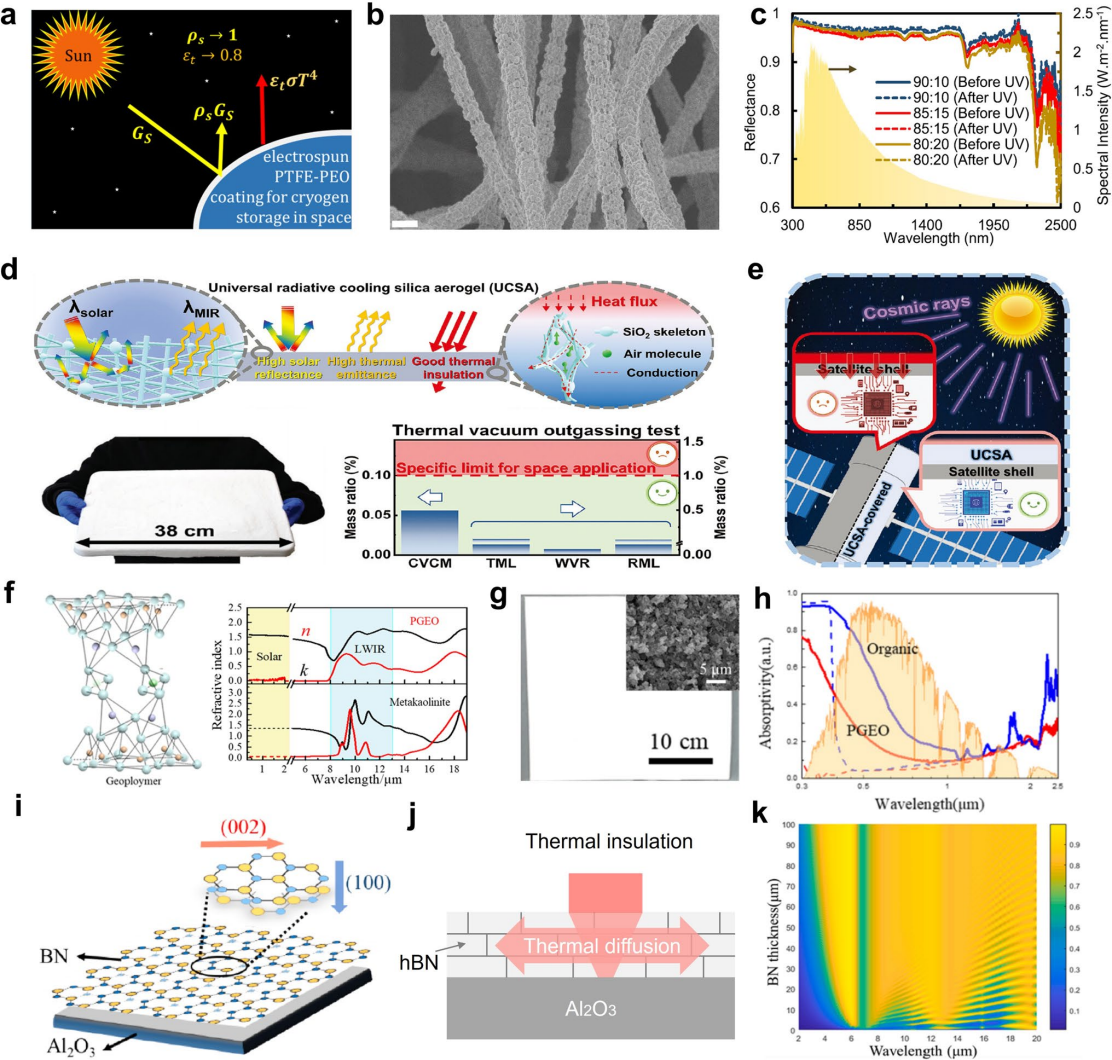
**a** Schematic of electrospun PTFE-PEO coating for cryogen storage in space. **b** SEM images of nanofibers. **c** Spectral hemispherical reflectance of the samples before and after UV exposure. **d** Schematic of the design method of UCSA (top). Photograph of large-area UCSA and its mass changes after the thermal vacuum outgassing test (bottom). **e** UCSA provides a promising cooling approach for thermal management in spacecraft applications. **f** The 3D network structure of the geopolymer (left). The refractive index of metakaolinite and the PGEO (right). **g** Photograph and SEM image of the PGEO coating surface. **h** Solar reflectance of PGEO and organic coatings before (dashed) and after (solid) space proton irradiation. **i** Schematic of the h-BN/Al_2_O_3_ dual-layer coating. **j** Schematic illustration of the heat transfer process. **k** Simulated emittance cross-sections of the bilayer h-BN/Al_2_O_3_ coating in the 0.2–20 μm range with different h-BN layer thicknesses. **a-c** Reproduced with permission [155]. Copyright 2024, American Chemical Society. **d-e** Reproduced with permission [41]. Copyright 2024, Wiley–VCH. **f-h** Reproduced with permission [156]. Copyright 2020, American Chemical Society. **i-k** Reproduced with permission [154]. Copyright 2021, Elsevier

Ibekwe et al. reported a polymeric nanofibrous film composed of PTFE and PEO for space radiative cooling (Fig. 15a) [155]. Thanks to nanoscale features (nanofibers and nanobeads) fabricated by the electrospinning method (Fig. 15b), this hybrid fibrous film achieves a maximum reflectance of over 99% at air mass 0 (AM0) and a MIR emittance of 81.42% when the thickness exceeds 3300 μm. The thicker film also exhibits great heat resistance to block objects from excessive heating. More importantly, due to the stable carbon–fluorine bonds in PTFE and its high UV reflectance, the optical performance is retained after a UV radiation dosage of 3.11 MJ m^−2^ (Fig. 15c). Under atomic oxygen exposure, the film can maintain a white appearance and distinctive nanostructures, indicating the excellence of atomic oxygen resistance. Ordered periodic structures hold significant potential for precise spectral manipulation, enabling broadband thermal radiation. Xiao et al. developed a distributed Bragg reflection consisting of 18 alternating layers of three materials (SiN, SiO_2_, and Ta_2_O_5_) [157]. Ta_2_O_5_ is to enhance absorption in the 13–25 μm range and improve reflection in the UV–visible region due to its high extinction coefficient above 13 μm and high refractive index in the UV–visible range. Combined with an Al mirror reflector, the optical solar reflector (OSR) possesses around 0.1 solar absorptance and around 0.75 broadband emittance in the range of 2.5–30 μm, with an ultrathin thickness of 2.088 μm. The multilayer structure can be fabricated on the flexible PI substrate or silicon substrate, with all materials capable of withstanding temperatures up to 350 °C, making them suitable for harsh space environments. Ding et al. designed SiO_2_ micropillar arrays that suppress infrared reflection (8–13 μm) and enhance emittance from 0.79 to 0.94 via surface phonon coupling [158]. Outdoor tests showed a daytime cooling of 5.5 °C under 843.1 W m^−2^ solar irradiance. The structures were successfully replicated on optical solar reflectors. As a promising alternative or supplement to orbital satellites, stratospheric airships face a major challenge of overheating. To tackle this, Fu et al. designed a multilayer nanoparticle–polymer metamaterial envelope for airships, featuring a hierarchical structure that includes a UV-blocking top layer, a nanoparticle composite layer (TiO_2_, far-infrared ceramic powder, and fluorescent brightener in a TPU matrix) for solar reflection and gas barrier functions, and a Kevlar fiber layer for mechanical reinforcement [159]. The flight experiments verified the cooling performance of the MNPM envelope, which exhibited a maximum temperature 15.4 °C lower than that of the silica/Ag group in the stratosphere.

Apart from MIR spectrum design, the addition of thermal conduction channels can further improve the thermal management performance of space radiative cooling devices. Lan et al. developed a large-area universal radiative cooling silica aerogel (UCSA) to constrain thermal conduction, reflect solar energy, and emit thermal radiation in the 2.5–25 μm band [41]. UCSA has the capacity to keep its low volatile constituents after ultra-high vacuum and tough thermal cycling (Fig. 15d). Compared to electrospun polyethylene oxide membrane (PEOM) and quartz fiber membrane (QFM), the UCSA exhibits the most excellent broadband solar reflection (99.1%) and MIR emission performance (90%). This is due to the synergistic effect of nanobeads and nanofibers. Moreover, UCSA is designed as a satellite protective layer to isolate heat and block the cosmic rays (Fig. 15e). According to the flame exposure experiment, UCSA can withstand direct exposure to a frontside temperature of 831.4 °C while maintaining a significantly lower temperature of around 100 °C on the backside, demonstrating extraordinarily high-temperature thermal insulation performance. Additionally, UCSA maintains its optical properties and cooling performance after damp heat (DH) and UV preconditioning tests with no changes in appearance. Chen et al. designed an all-inorganic phosphoric acid-based geopolymer (PGEO) paint with an amorphous geopolymer network (− Si − O − Al − O − P − O −) [156]. This unique structure achieves a moderately high refractive index and extinction coefficient within the broad MIR region, endowing the paint with approximately 95% MIR emittance (Fig. 15f). Different from the organic binder, the paint can achieve self-adhesion through low-polymeric P-O tetrahedral units to form a rough and porous network structure, showing a bright white surface and realizing over 90% solar light reflectance (Fig. 15g). When exposed to space proton radiation, the PGEO coating displays great spectral stability compared to organic-ZnO radiative cooling coating (Fig. 15h). In addition, the all-inorganic composition allows the coating to experience only a slight decrease in MIR emittance even at 1200 °C. Chen et al. introduced a dual-layer coating that possesses both anisotropic thermal conductivity and radiative cooling performance [154]. The in-plane alignment of the top-layer hexagonal boron nitride (h-BN) flakes enables the coating to conduct heat within the plane and insulate heat outside the plane (Fig. 15i). The hybrid phonon polaritons contributed by the top h-BN layer and bottom alumina (Al_2_O_3_) layer enable the coating to absorb most MIR radiation with a low thickness over the whole thermal wavelength range (Fig. 15j). It is noteworthy that the significant difference in thermal conductivity between the vertical and horizontal directions effectively transfers heat from the front surface facing the sun to the backlit side facing the cold universe while insulating the interior (Fig. 15k). Furthermore, proton irradiation from the universe has no significant impact on the chemical structures of h-BN and Al_2_O_3_, allowing the coating to retain reflectance above 0.75 at a wavelength of 500 nm.

## Challenges and Outlooks

In this review, we comprehensively overview the design principles behind extreme environmental cooling management and summarize the specific opening and closing requirements for heat exchange channels under a range of extreme conditions. This includes input heat channels capturing solar energy, atmospheric inverse radiation, and environmental parasitic heat, and output heat channels, encompassing mechanisms such as thermal radiation, water evaporation, and phase change latent heat [107, 114, 160]. While the previous sections have detailed recent advancements and specific strategies tailored for terrestrial dwelling, terrestrial extreme, aeronautical, and space environments, a summary of the persistent challenges and future perspectives is essential to guide the development of next-generation EERC.

### Current Challenges in Extreme Environmental Radiative Cooling

Despite recent milestones in the area of extreme environmental cooling management, several key challenges hinder the widespread applications of these technologies.

1. Limited multi-environment adaptability: Most radiative cooling systems are designed to adapt to only one or two specific scenarios, which significantly limits their commercial applications. In practice, such systems often operate in environments exposed simultaneously to UV radiation, flames, biological contamination (e.g., mold or bacteria), particulate matter, and corrosive rain (acidic or alkaline) [49, 78, 91, 161]. Existing materials and structures lack the robustness to maintain consistent performance under these combined stresses, hindering their long-term durability and reliability in real-world settings. Moreover, many high-performance EERC designs struggle with substrate compatibility while maintaining desired optical properties. These issues restrict mass production and drive up manufacturing costs.
2. Lack of dynamic environmental responsiveness: Extreme environments generally exhibit dynamic transformation characteristics, such as temperature fluctuations between hot days and cold nights in desert regions, alternating rainy and dry seasons in tropical areas, or variations in solar radiation between sunlight and shaded sides [162–164]. Most EERC systems are passive and static, lacking the ability to adapt in real time.
3. Insufficient performance: In scenarios involving excessive heat input or the forced closure of heat dissipation pathways, existing EERC systems may not provide sufficient thermal dissipation beyond the absorbed heating power. In such cases, reliance on radiative, conductive, evaporative, or latent heat transfer channels becomes insufficient, limiting the effectiveness of EERC in extreme environments.

### Constructive Countermeasures and Future Outlooks

**Fig. 16:**
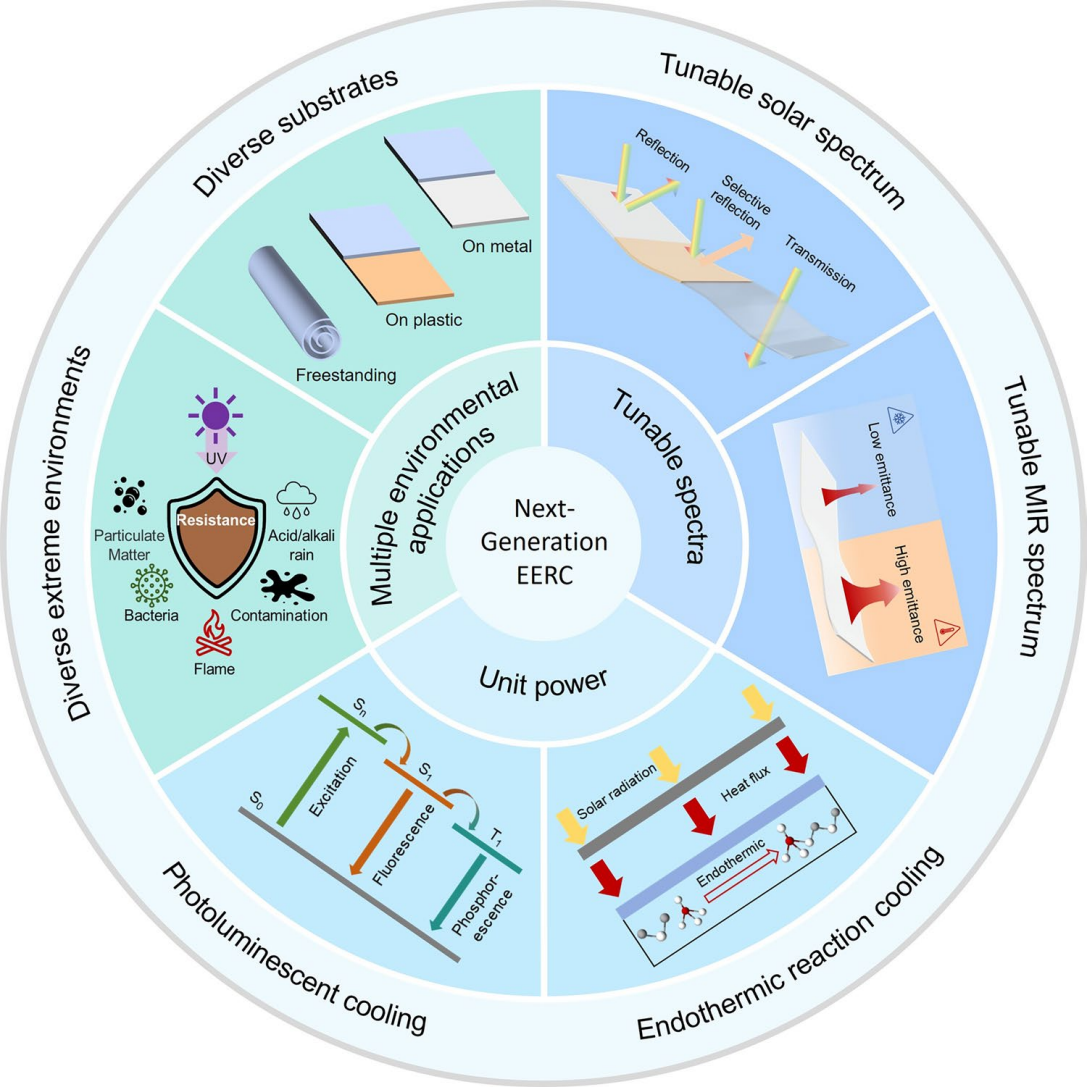
Schematic illustration of prospects for next-generation EERC

Addressing these challenges requires multidisciplinary efforts focusing on material innovation, structural design, and system integration. Based on the research reviewed, several constructive countermeasures and outlooks for the next-generation EERC can be proposed (Fig. 16).

1. Development of multi-environmental durability and substrate compatibility: EERC systems are required to integrate multi-functional protection, including UV resistance, flame retardancy, antimicrobial properties, particulate matter removal, and self-cleaning capabilities. Multilayered and hierarchically structured designs combining organic and inorganic materials can provide tailored protection while maintaining cooling performance. For example, micro-nano structures can endow materials with hydrophobic properties, significantly enhancing their resistance to humidity, icing, or contamination [165]. Yong et al. constructed hierarchical microstructures on a PTFE substrate through femtosecond laser processing, resulting in a superhydrophobic surface capable of maintaining underwater gas transport and stability under challenging conditions [166]. Meanwhile, EERC should be compatible with both rigid and flexible substrates while retaining freestanding functionality. To achieve this, scalable fabrication techniques, such as roll-to-roll fabrication, electrospinning, and coating methods, offer promising pathways to enable large-area, cost-effective production and facilitate seamless integration into everyday infrastructure [25, 167].
2. Tunable spectral manipulation: The next-generation EERC should be capable of dynamically modulating solar and MIR spectra in response to ambient conditions. Under cooling demand, the system exhibits high solar reflectance and MIR emittance to minimize heat absorption while maximizing cooling efficiency. Conversely, under heating demand, low solar reflectance and MIR emittance are preferred to retain thermal energy [168]. Beyond temperature regulation, materials with tunable spectral properties enable visible and infrared camouflage. Modulating visible reflectance allows surfaces to blend into diverse environments, such as forests, deserts, or skies, by shifting surface color [169]. Likewise, dynamic infrared emittance plays an important role in infrared concealment, particularly in complex and variable environments such as the day–night cycle or terrain transitions between jungles and deserts [170]. Additionally, in space applications such as solar panels, dynamic spectral regulation improves operational stability and efficiency. When exposed to direct solar radiation, solar panels must efficiently transmit useful wavelengths for energy harvesting while reflecting excess solar energy and emitting broadband thermal radiation to prevent overheating [171, 172]. In contrast, when the spacecraft transitions to the shaded side, where solar energy is absent, it becomes necessary to maximize the absorption of any available solar radiation and minimize thermal emission to capture solar energy and conserve heat [173]. To achieve these, various regulation strategies can be employed, including mechanical, liquid-based, thermal, and electrical controls, enabling the adjustment of solar reflectance, transmittance, and MIR emittance [174–178]. For instance, Sun et al. demonstrated a transparent smart radiative panel with an AZO layer, SiO_2_ spacer, and patterned VO_2_ [179]. An array of VO_2_ squares forms a metasurface that enhances visible light transmittance to 0.62 and achieves thermal emittance contrast (Δε) of 0.26, making it well-suited for space applications.
3. Hybrid cooling strategies: To improve the unit power of EERC systems, it is essential to introduce alternative heat exchange mechanisms. Photoluminescence can convert absorbed UV energy into visible-range fluorescence or phosphorescence, thereby mitigating UV-induced heating [180]. Its emission peaks can be tuned to produce various colors, enabling esthetically appealing surfaces without compromising radiative cooling [181]. In contexts where stealth or camouflage is desired, these materials can be engineered to match specific spectral backgrounds while still effectively rejecting solar heat. Beyond these benefits, photoluminescent materials enhance overall UV resistance by converting harmful UV radiation, thereby reducing UV-induced degradation and extending service life in extreme environments [182]. Additionally, endothermic reactions can store excess heat as chemical energy, providing supplementary cooling to improve adaptability in harsh environments. Remarkably, endothermic reactions can be reversed into exothermic reactions, releasing stored heat to the radiative cooling surface to increase its temperature. The reversible chemical process presents a promising approach for achieving dynamic thermal management [183, 184].

We hope that this review will be beneficial to understanding the relationship between customized heat dissipation channels and extreme environmental cooling management performance, and provide guidance and insights for their future development, helping to drive innovations that will address the ongoing challenges in the field of extreme environmental cooling management.

## References

[1] X. Yin, R. Yang, G. Tan, S. Fan, Terrestrial radiative cooling: Using the cold universe as a renewable and sustainable energy source. Science **370**(6518), 786–791 (2020). 10.1126/science.abb0971 33184205 10.1126/science.abb0971

[2] D. Zhao, A. Aili, Y. Zhai, J. Lu, D. Kidd et al., Subambient cooling of water: toward real-world applications of daytime radiative cooling. Joule **3**(1), 111–123 (2019). 10.1016/j.joule.2018.10.006

[3] D. Pan, Z. Han, J. Lei, Y. Niu, H. Liu et al., Core-shell structured BN/SiO_2_ nanofiber membrane featuring with dual-effect thermal management and flame retardancy for extreme space thermal protection. Sci. Bull. **70**(5), 722–732 (2025). 10.1016/j.scib.2025.01.005 10.1016/j.scib.2025.01.005 39827028

[4] C. Lin, K. Li, M. Li, B. Dopphoopha, J. Zheng et al., Pushing radiative cooling technology to real applications. Adv. Mater. (2024). 10.1002/adma.202409738 10.1002/adma.202409738 PMC12160687 39415410

[5] R. Xu, T. Ye, X. Yue, Z. Yang, W. Yu et al., Global population exposure to landscape fire air pollution from 2000 to 2019. Nature **621**(7979), 521–529 (2023). 10.1038/s41586-023-06398-6 37730866 10.1038/s41586-023-06398-6 PMC10511322

[6] L. Lin, X. Yi, H. Liu, R. Meng, S. Li et al., The airway microbiome mediates the interaction between environmental exposure and respiratory health in humans. Nat. Med. **29**(7), 1750–1759 (2023). 10.1038/s41591-023-02424-2 37349537 10.1038/s41591-023-02424-2

[7] J. Wei, J. Wang, Z. Li, S. Kondragunta, S. Anenberg et al., Long-term mortality burden trends attributed to black carbon and PM_2·5_ from wildfire emissions across the continental USA from 2000 to 2020: a deep learning modelling study. Lancet Planet. Health **7**(12), e963–e975 (2023). 10.1016/S2542-5196(23)00235-8 38056967 10.1016/S2542-5196(23)00235-8

[8] Y. Ying, J. Yu, B. Qin, M. Zhao, T. Zhou et al., Directional thermal emission covering two atmospheric windows. Laser Photonics Rev. **17**(11), 2300407 (2023). 10.1002/lpor.202300407

[9] R.H. Galib, Y. Tian, Y. Lei, S. Dang, X. Li et al., Atmospheric-moisture-induced polyacrylate hydrogels for hybrid passive cooling. Nat. Commun. **14**(1), 6707 (2023). 10.1038/s41467-023-42548-0 37872249 10.1038/s41467-023-42548-0 PMC10593860

[10] H. Long, S. Lei, F. Wang, S. Yang, H. Ju et al., Superhydrophobic daytime radiative cooling coating incorporated with phase change microcapsules for building thermal regulation. J. Mater. Sci. **59**(15), 6459–6475 (2024). 10.1007/s10853-024-09560-1

[11] S.K. Chamoli, W. Li, Visibly transparent multifunctional camouflage coating with efficient thermal management. Opt. Lett. **48**(16), 4340–4343 (2023). 10.1364/OL.494539 37582027 10.1364/OL.494539

[12] G.M. Hunt, A.B. Peters, J.B. Spicer, M.E. Thomas, High temperature optical performance of MgO: Y_2_O_3_ films for space applications. Int. J. Heat Mass Transf. **222**, 125114 (2024). 10.1016/j.ijheatmasstransfer.2023.125114

[13] S. Shrestha, C. Borrero del Pino, U. Malayoglu, Inorganic white thermal-control coatings for extreme space environments. J. Spacecr. Rockets **53**(6), 1061–1067 (2016). 10.2514/1.a33508

[14] L. Xu, D.-W. Sun, Y. Tian, T. Fan, Z. Zhu, Nanocomposite hydrogel for daytime passive cooling enabled by combined effects of radiative and evaporative cooling. Chem. Eng. J. **457**, 141231 (2023). 10.1016/j.cej.2022.141231

[15] Y. Bai, X. Jia, J. Yang, H. Song, Three birds with one stone strategy: a tri-modal radiator based on the cooling-compensation-heating effect. Nano Energy **127**, 109770 (2024). 10.1016/j.nanoen.2024.109770

[16] M. Lian, W. Ding, S. Liu, Y. Wang, T. Zhu et al., Highly porous yet transparent mechanically flexible aerogels realizing solar-thermal regulatory cooling. Nano-Micro Lett. **16**(1), 131 (2024). 10.1007/s40820-024-01356-x 10.1007/s40820-024-01356-x PMC10897091 38409640

[17] W. Xie, C. Xiao, Y. Sun, Y. Fan, B. Zhao et al., Flexible photonic radiative cooling films: fundamentals, fabrication and applications. Adv. Funct. Mater. **33**(46), 2305734 (2023). 10.1002/adfm.202305734

[18] W. Gao, Z. Lei, K. Wu, Y. Chen, Reconfigurable and renewable nano-micro-structured plastics for radiative cooling. Adv. Funct. Mater. **31**(21), 2100535 (2021). 10.1002/adfm.202100535

[19] H. Zhang, K.C.S. Ly, X. Liu, Z. Chen, M. Yan et al., Biologically inspired flexible photonic films for efficient passive radiative cooling. Proc. Natl. Acad. Sci. U.S.A. **117**(26), 14657–14666 (2020). 10.1073/pnas.2001802117 32541048 10.1073/pnas.2001802117 PMC7334532

[20] A.P. Raman, M.A. Anoma, L. Zhu, E. Rephaeli, S. Fan, Passive radiative cooling below ambient air temperature under direct sunlight. Nature **515**(7528), 540–544 (2014). 10.1038/nature13883 25428501 10.1038/nature13883

[21] Z. Ding, L. Pattelli, H. Xu, W. Sun, X. Li et al., Iridescent daytime radiative cooling with No absorption peaks in the visible range. Small **18**(25), 2202400 (2022). 10.1002/smll.202202400 10.1002/smll.202202400 35587771

[22] R. Shanker, P. Ravi Anusuyadevi, S. Gamage, T. Hallberg, H. Kariis et al., Structurally colored cellulose nanocrystal films as transreflective radiative coolers. ACS Nano **16**(7), 10156–10162 (2022). 10.1021/acsnano.1c10959

[23] R. Ali Yalçın, E. Blandre, K. Joulain, J. Drévillon, Colored radiative cooling coatings with nanoparticles. ACS Photonics **7**(5), 1312–1322 (2020). 10.1021/acsphotonics.0c00513

[24] S. Fan, W. Li, Photonics and thermodynamics concepts in radiative cooling. Nat. Photon. **16**(3), 182–190 (2022). 10.1038/s41566-021-00921-9

[25] D. Li, X. Liu, W. Li, Z. Lin, B. Zhu et al., Scalable and hierarchically designed polymer film as a selective thermal emitter for high-performance all-day radiative cooling. Nat. Nanotechnol. **16**(2), 153–158 (2021). 10.1038/s41565-020-00800-4 33199884 10.1038/s41565-020-00800-4

[26] Y. Zhai, Y. Ma, S.N. David, D. Zhao, R. Lou et al., Scalable-manufactured randomized glass-polymer hybrid metamaterial for daytime radiative cooling. Science **355**(6329), 1062–1066 (2017). 10.1126/science.aai7899 28183998 10.1126/science.aai7899

[27] Y. Xin, C. Li, W. Gao, Y. Chen, Emerging colored and transparent radiative cooling: fundamentals, progress, and challenges. Mater. Today **83**, 355–381 (2025). 10.1016/j.mattod.2024.12.012

[28] X. Wu, J. Li, F. Xie, X.-E. Wu, S. Zhao et al., A dual-selective thermal emitter with enhanced subambient radiative cooling performance. Nat. Commun. **15**(1), 815 (2024). 10.1038/s41467-024-45095-4 38280849 10.1038/s41467-024-45095-4 PMC10821923

[29] B.E. Psiloglou, M. Santamouris, D.N. Asimakopoulos, Predicting the broadband transmittance of the uniformly mixed gases (CO_2_, CO, N_2_O, CH_4_ and O_2_) in the atmosphere, for solar radiation models. Renew. Energy **6**(1), 63–70 (1995). 10.1016/0960-1481(94)00062-B

[30] T. Fang, General discussion on displacement law on radiation. Int. Commun. Heat Mass Transf. **30**(5), 737–743 (2003). 10.1016/S0735-1933(03)00111-8

[31] X. Sun, Y. Sun, Z. Zhou, M.A. Alam, P. Bermel, Radiative sky cooling: fundamental physics, materials, structures, and applications. Nanophotonics **6**(5), 20 (2017). 10.1515/nanoph-2017-0020

[32] Y. Wu, J. Luo, M. Pu, B. Liu, J. Jin et al., Optically transparent infrared selective emitter for visible-infrared compatible camouflage. Opt. Express **30**(10), 17259–17269 (2022). 10.1364/OE.457547 36221552 10.1364/OE.457547

[33] S. Jiao, K. Zhao, J. Jiang, K. Zhao, Q. Guo et al., Metasurface with all-optical tunability for spatially-resolved and multilevel thermal radiation. Nanophotonics **13**(9), 1645–1655 (2024). 10.1515/nanoph-2024-0005 39678182 10.1515/nanoph-2024-0005 PMC11636409

[34] A. Reicks, A. Tsubaki, M. Anderson, J. Wieseler, L.K. Khorashad et al., Near-unity broadband omnidirectional emissivity *via* femtosecond laser surface processing. Commun. Mater. **2**, 36 (2021). 10.1038/s43246-021-00139-w

[35] K.-T. Lin, J. Han, K. Li, C. Guo, H. Lin et al., Radiative cooling: Fundamental physics, atmospheric influences, materials and structural engineering, applications and beyond. Nano Energy **80**, 105517 (2021). 10.1016/j.nanoen.2020.105517

[36] D. Han, C. Wang, C.B. Han, Y. Cui, W.R. Ren et al., Highly optically selective and thermally insulating porous calcium silicate composite SiO_2_ aerogel coating for daytime radiative cooling. ACS Appl. Mater. Interf. **16**(7), 9303–9312 (2024). 10.1021/acsami.3c18101 10.1021/acsami.3c18101 38343044

[37] T.N. Narasimhan, Fourier’s heat conduction equation: History, influence, and connections. Proc. Indian Acad. Sci. (Earth Planet Sci.) **108**(3), 117–148 (1999). 10.1007/bf02842327

[38] R. Hu, Y. Liu, S. Shin, S. Huang, X. Ren et al., Emerging materials and strategies for personal thermal management. Adv. Energy Mater. **10**(17), 1903921 (2020). 10.1002/aenm.201903921

[39] Z. Lu, E. Strobach, N. Chen, N. Ferralis, J.C. Grossman, Passive sub-ambient cooling from a transparent evaporation-insulation bilayer. Joule **4**(12), 2693–2701 (2020). 10.1016/j.joule.2020.10.005

[40] H. Zhu, Q. Li, C. Zheng, Y. Hong, Z. Xu et al., High-temperature infrared camouflage with efficient thermal management. Light. Sci. Appl. **9**, 60 (2020). 10.1038/s41377-020-0300-5 32337024 10.1038/s41377-020-0300-5 PMC7156722

[41] P.-H. Lan, C.-W. Hwang, T.-C. Chen, T.-W. Wang, H.-L. Chen et al., Hierarchical ceramic nanofibrous aerogels for universal passive radiative cooling. Adv. Funct. Mater. **34**(52), 2410285 (2024). 10.1002/adfm.202410285

[42] H. Zhai, C. Liu, D. Fan, Q. Li, Dual-encapsulated nanocomposite for efficient thermal buffering in heat-generating radiative cooling. ACS Appl. Mater. Interfaces **14**(51), 57215–57224 (2022). 10.1021/acsami.2c13991 36484240 10.1021/acsami.2c13991

[43] Y. Du, Y. Chen, J. Liu, Y. Liang, X. Yang et al., Boosting thermoelectric generator (TEG) performance with tandem radiative/evaporative/phase change cooler. Nano Energy **128**, 109909 (2024). 10.1016/j.nanoen.2024.109909

[44] C. Feng, P. Yang, H. Liu, M. Mao, Y. Liu et al., Bilayer porous polymer for efficient passive building cooling. Nano Energy **85**, 105971 (2021). 10.1016/j.nanoen.2021.105971

[45] J. Cao, Y. Huang, Z. Chen, H. Yan, M. Chen, Radiative cooling coupled with latent heat storage for dynamic thermal management. Sol. Energy Mater. Sol. Cells **278**, 113173 (2024). 10.1016/j.solmat.2024.113173

[46] W. Lin, X. Yao, N.M. Kumar, W.K. Lo, S.S. Chopra et al., Camel-fur-inspired graphite-based hygroscopic membrane for passive air cooling with ultrahigh cooling power. Adv. Energy Mater. **14**(16), 2303470 (2024). 10.1002/aenm.202303470

[47] Q. Xin, B. Ma, J. Ru, Y. Zhou, D. Jing, Efficient passive cooling over a novel bifunctional polymer bilayer composite simultaneously possessing radiative and evaporative cooling properties. Adv. Energy Mater. **15**(14), 2404122 (2025). 10.1002/aenm.202404122

[48] J. Li, X. Wang, D. Liang, N. Xu, B. Zhu et al., A tandem radiative/evaporative cooler for weather-insensitive and high-performance daytime passive cooling. Sci. Adv. **8**(32), eabq0411 (2022). 10.1126/sciadv.abq0411 35960798 10.1126/sciadv.abq0411 PMC9374334

[49] Q. Zhang, T. Wang, R. Du, J. Zheng, H. Wei et al., Highly stable polyimide composite nanofiber membranes with spectrally selective for passive daytime radiative cooling. ACS Appl. Mater. Interf. **16**(30), 40069–40076 (2024). 10.1021/acsami.4c09549 10.1021/acsami.4c09549 39037051

[50] F. Xie, W. Jin, J.R. Nolen, H. Pan, N. Yi et al., Subambient daytime radiative cooling of vertical surfaces. Science **386**(6723), 788–794 (2024). 10.1126/science.adn2524 39541474 10.1126/science.adn2524

[51] R. Wu, C. Sui, T.-H. Chen, Z. Zhou, Q. Li et al., Spectrally engineered textile for radiative cooling against urban heat islands. Science **384**(6701), 1203–1212 (2024). 10.1126/science.adl0653 38870306 10.1126/science.adl0653

[52] C. Lin, Y. Li, C. Chi, Y.S. Kwon, J. Huang et al., A solution-processed inorganic emitter with high spectral selectivity for efficient subambient radiative cooling in hot humid climates. Adv. Mater. **34**(12), 2109350 (2022). 10.1002/adma.202109350 10.1002/adma.202109350 35038775

[53] Y. Tian, X. Liu, J. Li, A. Caratenuto, S. Zhou et al., Scalable, fire-retardant, and spectrally robust melamine-formaldehyde photonic bulk for efficient daytime radiative cooling. Appl. Mater. Today **24**, 101103 (2021). 10.1016/j.apmt.2021.101103

[54] X. Wu, J. Li, Q. Jiang, W. Zhang, B. Wang et al., An all-weather radiative human body cooling textile. Nat. Sustain. **6**(11), 1446–1454 (2023). 10.1038/s41893-023-01200-x

[55] X. Liu, M. Zhang, Y. Hou, Y. Pan, C. Liu et al., Hierarchically superhydrophobic stereo-complex poly (lactic acid) aerogel for daytime radiative cooling. Adv. Funct. Mater. **32**(46), 2207414 (2022). 10.1002/adfm.202207414

[56] H. Liu, J. Yu, S. Zhang, B. Ding, Air-conditioned masks using nanofibrous networks for daytime radiative cooling. Nano Lett. **22**(23), 9485–9492 (2022). 10.1021/acs.nanolett.2c03585 36469697 10.1021/acs.nanolett.2c03585

[57] X.-E. Wu, Y. Wang, X. Liang, Y. Zhang, P. Bi et al., Durable radiative cooling multilayer silk textile with excellent comprehensive performance. Adv. Funct. Mater. **34**(11), 2313539 (2024). 10.1002/adfm.202313539

[58] Y. Jung, M. Kim, S. Jeong, S. Hong, S.H. Ko, Strain-insensitive outdoor wearable electronics by thermally robust nanofibrous radiative cooler. ACS Nano **18**(3), 2312–2324 (2024). 10.1021/acsnano.3c10241 38190550 10.1021/acsnano.3c10241

[59] J. Li, Y. Liang, W. Li, N. Xu, B. Zhu et al., Protecting ice from melting under sunlight *via* radiative cooling. Sci. Adv. **8**(6), eabj9756 (2022). 10.1126/sciadv.abj9756 35148187 10.1126/sciadv.abj9756 PMC8836806

[60] H. Fan, K. Wang, Y. Ding, Y. Qiang, Z. Yang et al., Core–shell composite nanofibers with high temperature resistance, hydrophobicity and breathability for efficient daytime passive radiative cooling. Adv. Mater. **36**(40), 2406987 (2024). 10.1002/adma.202406987 10.1002/adma.202406987 39194411

[61] X. Meng, Z. Chen, C. Qian, Q. Li, X. Chen, Durable and mechanically robust superhydrophobic radiative cooling coating. Chem. Eng. J. **478**, 147341 (2023). 10.1016/j.cej.2023.147341

[62] J. Xu, F. Liang, Z. Wang, X. Chao, Y. Gu et al., A durable, breathable, and weather-adaptive coating driven by particle self-assembly for radiative cooling and energy harvesting. Nano Energy **124**, 109489 (2024). 10.1016/j.nanoen.2024.109489

[63] G. Li, J. Huang, J. Zhou, Y. Zhang, C. Zhang et al., A flame-retardant wood-based composite with magnesium–aluminium layered double hydroxides for efficient daytime radiative cooling. J. Mater. Chem. A **12**(3), 1609–1616 (2024). 10.1039/D3TA06065A

[64] Z. Cheng, H. Han, F. Wang, Y. Yan, X. Shi et al., Efficient radiative cooling coating with biomimetic human skin wrinkle structure. Nano Energy **89**, 106377 (2021). 10.1016/j.nanoen.2021.106377

[65] W. Heng, S. Yin, J. Min, C. Wang, H. Han et al., A smart mask for exhaled breath condensate harvesting and analysis. Science **385**(6712), 954–961 (2024). 10.1126/science.adn6471 39208112 10.1126/science.adn6471 PMC12168143

[66] P. Yao, Z. Chen, T. Liu, X. Liao, Z. Yang et al., Spider-silk-inspired nanocomposite polymers for durable daytime radiative cooling. Adv. Mater. **34**(51), e2208236 (2022). 10.1002/adma.202208236 36255146 10.1002/adma.202208236

[67] X. Cai, L. Gao, J. Wang, D. Li, MOF-integrated hierarchical composite fiber for efficient daytime radiative cooling and antibacterial protective textiles. ACS Appl. Mater. Interfaces **15**(6), 8537–8545 (2023). 10.1021/acsami.2c21832 36726324 10.1021/acsami.2c21832

[68] B.-B. Li, G.-L. Zhang, Q.-K. Xue, P. Luo, X. Zhao et al., Rational design and fine fabrication of passive daytime radiative cooling textiles integrate antibacterial, UV-shielding, and self-cleaning characteristics. ACS Appl. Mater. Interfaces **16**(39), 52633–52644 (2024). 10.1021/acsami.4c10161 39300615 10.1021/acsami.4c10161

[69] Y. Xin, W. Gao, C. Zhang, Y. Chen, Scalable and sustainable radiative cooling enabled by renewable poplar catkin-derived films. Energy **290**, 130186 (2024). 10.1016/j.energy.2023.130186

[70] Y. Chen, B. Dang, J. Fu, C. Wang, C. Li et al., Cellulose-based hybrid structural material for radiative cooling. Nano Lett. **21**(1), 397–404 (2021). 10.1021/acs.nanolett.0c03738 33301320 10.1021/acs.nanolett.0c03738

[71] Y. Li, G. Zhang, K. Xu, M. Wu, H. Guo et al., A micro-sandwich-structured membrane with high solar reflectivity for durable radiative cooling. Matter **7**(12), 4297–4308 (2024). 10.1016/j.matt.2024.08.020

[72] Y. Zhang, X. Du, J. Huangfu, K. Chen, X. Han et al., Self-cleaning PTFE nanofiber membrane for long-term passive daytime radiative cooling. Chem. Eng. J. **490**, 151831 (2024). 10.1016/j.cej.2024.151831

[73] L. Li, Q. Zhang, G. Liu, R. Shi, H. Zhao et al., Durable hybrid metamaterial with hierarchically porous structure for efficient passive daytime radiative cooling. Chem. Eng. J. **498**, 155516 (2024). 10.1016/j.cej.2024.155516

[74] C. Cai, F. Chen, Z. Wei, C. Ding, Y. Chen et al., Large scalable, anti-ultraviolet, strong cellulose film with well-defined dual-pores for longtime daytime radiative cooling. Chem. Eng. J. **476**, 146668 (2023). 10.1016/j.cej.2023.146668

[75] Z. Yang, T. Chen, X. Tang, F. Xu, J. Zhang, Hierarchical fabric emitter for highly efficient passive radiative heat release. Adv. Fiber Mater. **5**(4), 1367–1377 (2023). 10.1007/s42765-023-00271-x

[76] X. Li, L. Pattelli, Z. Ding, M. Chen, T. Zhao et al., A novel BST@TPU membrane with superior UV durability for highly efficient daytime radiative cooling. Adv. Funct. Mater. **34**(23), 2315315 (2024). 10.1002/adfm.202315315

[77] J. Zhou, C. Ding, X. Zhang, D. Li, D. Yang et al., High-durable, radiative-cooling, and heat-insulating flexible films enabled by a bioinspired *Dictyophora*-like structure. ACS Appl. Mater. Interfaces (2023). 10.1021/acsami.3c14310 10.1021/acsami.3c14310 38032275

[78] X. Zhou, Y. Xu, D. Zhang, M. Huang, M. Liu, Robust and wear-durable coating based on halloysite nanotubes/polymer composite for passive daytime radiative cooling. Compos. Sci. Technol. **251**, 110566 (2024). 10.1016/j.compscitech.2024.110566

[79] L. Qi, W. Cai, T. Cui, L. Chen, J. Gao et al., Enhanced radiative cooling and flame retardancy through phosphate-linked hollow metal-organic framework spheres. Chem. Eng. J. **507**, 160469 (2025). 10.1016/j.cej.2025.160469

[80] J. Song, W. Zhang, Z. Sun, M. Pan, F. Tian et al., Durable radiative cooling against environmental aging. Nat. Commun. **13**, 4805 (2022). 10.1038/s41467-022-32409-7 35973997 10.1038/s41467-022-32409-7 PMC9381728

[81] J. Xu, X. Wu, Y. Li, S. Zhao, F. Lan et al., High-performance radiative cooling sunscreen. Nano Lett. **24**(47), 15178–15185 (2024). 10.1021/acs.nanolett.4c04969 39546330 10.1021/acs.nanolett.4c04969

[82] M. Li, C. Lin, K. Li, W. Ma, B. Dopphoopha et al., A UV-reflective organic–inorganic tandem structure for efficient and durable daytime radiative cooling in harsh climates. Small **19**(29), 2301159 (2023). 10.1002/smll.202301159 10.1002/smll.202301159 37178354

[83] Y. Sun, H. He, X. Huang, Z. Guo, Superhydrophobic SiO_2_-glass bubbles composite coating for stable and highly efficient daytime radiative cooling. ACS Appl. Mater. Interfaces **15**(3), 4799–4813 (2023). 10.1021/acsami.2c18774 36635243 10.1021/acsami.2c18774

[84] X. Li, J. Peoples, P. Yao, X. Ruan, Ultrawhite BaSO_4_ paints and films for remarkable daytime subambient radiative cooling. ACS Appl. Mater. Interfaces **13**(18), 21733–21739 (2021). 10.1021/acsami.1c02368 33856776 10.1021/acsami.1c02368

[85] S. Li, X. Zhang, Y. Yang, X. Li, H. Xu et al., An inorganic water-based paint for high-durability passive radiative cooling. J. Mater. Chem. C **13**(8), 4137–4144 (2025). 10.1039/d4tc04108a

[86] Y. Liu, X. Bu, T. Yu, X. Wang, M. He et al., Design and scalable fabrication of core-shell nanospheres embedded spectrally selective single-layer coatings for durable daytime radiative cooling. Sol. Energy Mater. Sol. Cells **260**, 112493 (2023). 10.1016/j.solmat.2023.112493

[87] H. Kang, Y. Qiao, Y. Li, W. Qin, X. Wu, Keep cool: polyhedral ZnO@ZIF-8 polymer coatings for daytime radiative cooling. Ind. Eng. Chem. Res. **59**(34), 15226–15232 (2020). 10.1021/acs.iecr.0c01178

[88] J. Zhao, Q. Meng, Y. Li, Z. Yang, J. Li, Structural porous ceramic for efficient daytime subambient radiative cooling. ACS Appl. Mater. Interfaces **15**(40), 47286–47293 (2023). 10.1021/acsami.3c10772 37751606 10.1021/acsami.3c10772

[89] Y. Xin, Q. Wang, C. Fu, S. Du, L. Hou et al., Alumina fiber membrane prepared by electrospinning technology for passive daytime radiative cooling. Adv. Funct. Mater. **35**(3), 2413813 (2025). 10.1002/adfm.202413813

[90] Y. Tian, X. Liu, Z. Wang, J. Li, Y. Mu et al., Subambient daytime cooling enabled by hierarchically architected all-inorganic metapaper with enhanced thermal dissipation. Nano Energy **96**, 107085 (2022). 10.1016/j.nanoen.2022.107085

[91] X. Zhao, T. Li, H. Xie, H. Liu, L. Wang et al., A solution-processed radiative cooling glass. Science **382**(6671), 684–691 (2023). 10.1126/science.adi2224 37943922 10.1126/science.adi2224

[92] K. Lin, S. Chen, Y. Zeng, T.C. Ho, Y. Zhu et al., Hierarchically structured passive radiative cooling ceramic with high solar reflectivity. Science **382**(6671), 691–697 (2023). 10.1126/science.adi4725 37943925 10.1126/science.adi4725

[93] U. Banik, A. Agrawal, H. Meddeb, O. Sergeev, N. Reininghaus et al., Efficient thin polymer coating as a selective thermal emitter for passive daytime radiative cooling. ACS Appl. Mater. Interfaces **13**(20), 24130–24137 (2021). 10.1021/acsami.1c04056 33974398 10.1021/acsami.1c04056

[94] X. Wang, D. Liu, Z. Wan, Z. Wang, J. Yu et al., A gradient nanoporous radiative cooling ceramic with high spectral selectivity. Chem. Eng. J. **500**, 157344 (2024). 10.1016/j.cej.2024.157344

[95] M.-T. Tsai, S.-W. Chang, Y.-J. Chen, H.-L. Chen, P.-H. Lan et al., Scalable, flame-resistant, superhydrophobic ceramic metafibers for sustainable all-day radiative cooling. Nano Today **48**, 101745 (2023). 10.1016/j.nantod.2022.101745

[96] N.N. Shi, C.-C. Tsai, F. Camino, G.D. Bernard, N. Yu et al., Keeping cool: Enhanced optical reflection and radiative heat dissipation in Saharan silver ants. Science **349**(6245), 298–301 (2015). 10.1126/science.aab3564 26089358 10.1126/science.aab3564

[97] C. Ma, Y. Gao, Y. Cao, Y. Yang, W. Wang et al., Hierarchically core-shell nanofiber textiles for personal cooling in hot and humid conditions. Nano Energy **123**, 109400 (2024). 10.1016/j.nanoen.2024.109400

[98] Y. Sun, Y. Ji, M. Javed, X. Li, Z. Fan et al., Preparation of passive daytime cooling fabric with the synergistic effect of radiative cooling and evaporative cooling. Adv. Mater. Technol. **7**(3), 2100803 (2022). 10.1002/admt.202100803

[99] J. Xu, J. Qiu, Effect of global climate change on the sub-ambient radiative cooling performance of ideal coolers in different environments. Int. Commun. Heat Mass Transf. **163**, 108705 (2025). 10.1016/j.icheatmasstransfer.2025.108705

[100] W. Tang, Y. Zhan, J. Yang, X. Meng, X. Zhu et al., Cascaded heteroporous nanocomposites for thermo-adaptive passive radiation cooling. Adv. Mater. **36**(36), e2310923 (2024). 10.1002/adma.202310923 39075820 10.1002/adma.202310923

[101] A. Leroy, B. Bhatia, C.C. Kelsall, A. Castillejo-Cuberos, H. Di Capua et al., High-performance subambient radiative cooling enabled by optically selective and thermally insulating polyethylene aerogel. Sci. Adv. **5**(10), eaat9480 (2019). 10.1126/sciadv.aat9480 31692957 10.1126/sciadv.aat9480 PMC6821464

[102] J. Yuan, H. Yin, D. Yuan, Y. Yang, S. Xu, On daytime radiative cooling using spectrally selective metamaterial based building envelopes. Energy **242**, 122779 (2022). 10.1016/j.energy.2021.122779

[103] Z.F. Mira, S.-Y. Heo, D.H. Kim, G.J. Lee, Y.M. Song, Multilayer selective passive daytime radiative cooler optimization utilizing memetic algorithm. J. Quant. Spectrosc. Radiat. Transf. **272**, 107774 (2021). 10.1016/j.jqsrt.2021.107774

[104] H. Yin, H. Yao, Y. Jia, J. Wang, C. Fan, Realization of efficient radiative cooling in thermal emitter with inorganic metamaterials. J. Phys. D Appl. Phys. **54**(34), 345501 (2021). 10.1088/1361-6463/ac0659

[105] Y. Wang, X. Zhang, S. Liu, Y. Liu, Q. Zhou et al., Thermal-rectified gradient porous polymeric film for solar-thermal regulatory cooling. Adv. Mater. **36**(26), e2400102 (2024). 10.1002/adma.202400102 38606728 10.1002/adma.202400102

[106] Y. Yu, L. Wei, Z. Pang, J. Wu, Y. Dong et al., Multifunctional wood composite aerogel with integrated radiant cooling and fog–water harvesting for all-day building energy conservation. Adv. Funct. Mater. **35**(5), 2414590 (2025). 10.1002/adfm.202414590

[107] Y. Liu, X. Bu, R. Liu, M. Feng, Z. Zhang et al., Robust fluorinated cellulose composite aerogels incorporating radiative cooling and thermal insulation for regionally adaptable building thermal management. Int. J. Biol. Macromol. **292**, 139239 (2025). 10.1016/j.ijbiomac.2024.139239 39733887 10.1016/j.ijbiomac.2024.139239

[108] H. Zhong, Y. Li, P. Zhang, S. Gao, B. Liu et al., Hierarchically hollow microfibers as a scalable and effective thermal insulating cooler for buildings. ACS Nano **15**(6), 10076–10083 (2021). 10.1021/acsnano.1c01814 34014070 10.1021/acsnano.1c01814

[109] M. Qin, H. Han, F. Xiong, Z. Shen, Y. Jin et al., Vapor exchange induced particles-based sponge for scalable and efficient daytime radiative cooling. Adv. Funct. Mater. **33**(44), 2304073 (2023). 10.1002/adfm.202304073

[110] C. Cai, W. Chen, Z. Wei, C. Ding, B. Sun et al., Bioinspired “aerogel grating” with metasurfaces for durable daytime radiative cooling for year-round energy savings. Nano Energy **114**, 108625 (2023). 10.1016/j.nanoen.2023.108625

[111] L. Zhou, J. Rada, H. Zhang, H. Song, S. Mirniaharikandi et al., Sustainable and inexpensive polydimethylsiloxane sponges for daytime radiative cooling. Adv. Sci. **8**(23), e2102502 (2021). 10.1002/advs.202102502 10.1002/advs.202102502 PMC8655219 34672111

[112] M. Yang, W. Zou, J. Guo, Z. Qian, H. Luo et al., Bioinspired “skin” with cooperative thermo-optical effect for daytime radiative cooling. ACS Appl. Mater. Interfaces **12**(22), 25286–25293 (2020). 10.1021/acsami.0c03897 32378874 10.1021/acsami.0c03897

[113] J. Fei, D. Han, X. Zhang, K. Li, N. Lavielle et al., Ultrahigh passive cooling power in hydrogel with rationally designed optofluidic properties. Nano Lett. **24**(2), 623–631 (2024). 10.1021/acs.nanolett.3c03694 38048272 10.1021/acs.nanolett.3c03694

[114] X. Hu, P. Hu, L. Liu, L. Zhao, S. Dou et al., Lightweight and hierarchically porous hydrogels for wearable passive cooling under extreme heat stress. Matter **7**(12), 4398–4409 (2024). 10.1016/j.matt.2024.09.008

[115] Z. Hu, Y. Qiu, J. Zhou, Q. Li, Smart flexible porous bilayer for all-day dynamic passive cooling. Small Sci. **4**(3), 2300237 (2024). 10.1002/smsc.202300237 40212687 10.1002/smsc.202300237 PMC11935011

[116] X. Liu, P. Li, Y. Liu, C. Zhang, M. He et al., Hybrid passive cooling for power equipment enabled by metal-organic framework. Adv. Mater. **36**(45), e2409473 (2024). 10.1002/adma.202409473 39240041 10.1002/adma.202409473

[117] C. Fan, Y. Zhang, Z. Long, A. Mensah, Q. Wang et al., Dynamically tunable subambient daytime radiative cooling metafabric with Janus wettability. Adv. Funct. Mater. **33**(29), 2300794 (2023). 10.1002/adfm.202300794

[118] B. Gu, F. Fan, Q. Xu, D. Shou, D. Zhao, A nano-structured bilayer asymmetric wettability textile for efficient personal thermal and moisture management in high-temperature environments. Chem. Eng. J. **461**, 141919 (2023). 10.1016/j.cej.2023.141919

[119] Y. Wang, Z. Wang, H. Huang, Y. Li, W. Zhai, A camel-fur-inspired micro-extrusion foaming porous elastic fiber for all-weather dual-mode human thermal regulation. Adv. Sci. **11**(43), 2407260 (2024). 10.1002/advs.202407260 10.1002/advs.202407260 PMC11578328 39340821

[120] L. Xu, D.-W. Sun, Y. Tian, L. Sun, Z. Zhu, *Cactus*-inspired bilayer cooler for high-performance and long-term daytime passive cooling. Chem. Eng. J. **489**, 151258 (2024). 10.1016/j.cej.2024.151258

[121] M. Qin, K. Jia, A. Usman, S. Han, F. Xiong et al., High-efficiency thermal-shock resistance enabled by radiative cooling and latent heat storage. Adv. Mater. **36**(25), 2314130 (2024). 10.1002/adma.202314130 10.1002/adma.202314130 38428436

[122] P. Li, Y. Liu, X. Liu, A. Wang, W. Liu et al., Reversed yolk–shell dielectric scatterers for advanced radiative cooling. Adv. Funct. Mater. **34**(23), 2315658 (2024). 10.1002/adfm.202315658

[123] S. Wang, M. Wu, H. Han, R. Du, Z. Zhao et al., Regulating cold energy from the universe by bifunctional phase change materials for sustainable cooling. Adv. Energy Mater. **14**(45), 2402667 (2024). 10.1002/aenm.202402667

[124] Z. Yan, H. Zhai, D. Fan, Q. Li, A trimode textile designed with hierarchical core-shell nanofiber structure for all-weather radiative personal thermal management. Nano Today **51**, 101897 (2023). 10.1016/j.nantod.2023.101897

[125] B. Gu, Z. Dai, H. Pan, D. Zhao, Integration of prolonged phase-change thermal storage material and radiative cooling textile for personal thermal management. Chem. Eng. J. **493**, 152637 (2024). 10.1016/j.cej.2024.152637

[126] W. Jiang, T. Zhu, J. Chen, Q. Liu, Y. Liu et al., Phase change foam with temperature-adaptive radiative cooling feature for all-day building energy saving. Chem. Eng. J. **502**, 157862 (2024). 10.1016/j.cej.2024.157862

[127] X. Zhang, T. Zuo, M. Ai, D. Yu, W. Wang, All-in-one cast-molded hydrophobic silicon dioxide-phase change microcapsule/gelatin-hydroxyethyl cellulose composite aerogel for building cooling. ACS Sustain. Chem. Eng. **12**(28), 10423–10435 (2024). 10.1021/acssuschemeng.4c02060

[128] Z. Zhu, A. Bashir, X. Wu, C. Liu, Y. Zhang et al., Highly integrated phase change and radiative cooling fiber membrane for adaptive personal thermal regulation. Adv. Funct. Mater. **35**(9), 2416111 (2025). 10.1002/adfm.202416111

[129] B. Khalichi, A. Ghobadi, A. Kalantari Osgouei, Z. Rahimian Omam, H. Kocer et al., Phase-change Fano resonator for active modulation of thermal emission. Nanoscale **15**(25), 10783–10793 (2023). 10.1039/d3nr00673e 37326249 10.1039/d3nr00673e

[130] B. Qin, Y. Zhu, Y. Zhou, M. Qiu, Q. Li, Whole-infrared-band camouflage with dual-band radiative heat dissipation. Light Sci. Appl. **12**(1), 246 (2023). 10.1038/s41377-023-01287-z 37794015 10.1038/s41377-023-01287-z PMC10550919

[131] X. Wang, Y. Tang, Y. Wang, L. Ke, X. Ye et al., Leather enabled multifunctional thermal camouflage armor. Chem. Eng. Sci. **196**, 64–71 (2019). 10.1016/j.ces.2018.12.005

[132] L. Wang, Y. Yang, X. Tang, B. Li, Y. Hu et al., Combined multi-band infrared camouflage and thermal management *via* a simple multilayer structure design. Opt. Lett. **46**(20), 5224–5227 (2021). 10.1364/OL.441605 34653158 10.1364/OL.441605

[133] H. Zhu, Q. Li, C. Tao, Y. Hong, Z. Xu et al., Multispectral camouflage for infrared, visible, lasers and microwave with radiative cooling. Nat. Commun. **12**(1), 1805 (2021). 10.1038/s41467-021-22051-0 33753740 10.1038/s41467-021-22051-0 PMC7985314

[134] L. Peng, D. Liu, H. Cheng, S. Zhou, M. Zu, A multilayer film based selective thermal emitter for infrared stealth technology. Adv. Opt. Mater. **6**(23), 1801006 (2018). 10.1002/adom.201801006

[135] W. Zhang, G. Xu, J. Zhang, H. Wang, H. Hou, Infrared spectrally selective low emissivity from Ge/ZnS one-dimensional heterostructure photonic crystal. Opt. Mater. **37**, 343–346 (2014). 10.1016/j.optmat.2014.06.023

[136] X. Jiang, H. Yuan, X. He, T. Du, H. Ma et al., Implementing of infrared camouflage with thermal management based on inverse design and hierarchical metamaterial. Nanophotonics **12**(10), 1891–1902 (2023). 10.1515/nanoph-2023-0067 39635145 10.1515/nanoph-2023-0067 PMC11501626

[137] K.C.S. Ly, X. Liu, X. Song, C. Xiao, P. Wang et al., A dual-mode infrared asymmetric photonic structure for all-season passive radiative cooling and heating. Adv. Funct. Mater. **32**(31), 2203789 (2022). 10.1002/adfm.202203789

[138] P. Wang, H. Wang, Y. Sun, M. Zhang, S. Chen et al., Transparent grating-based metamaterials for dynamic infrared radiative regulation smart windows. Phys. Chem. Chem. Phys. **26**(22), 16253–16260 (2024). 10.1039/d4cp01245c 38804578 10.1039/d4cp01245c

[139] D. Liu, Y. Xu, Y. Xuan, Fabry-Perot-resonator-coupled metal pattern metamaterial for infrared suppression and radiative cooling. Appl. Opt. **59**(23), 6861–6867 (2020). 10.1364/AO.392310 32788776 10.1364/AO.392310

[140] K. Yu, W. Zhang, M. Qian, P. Shen, Y. Liu, Multiband metamaterial emitters for infrared and laser compatible stealth with thermal management based on dissipative dielectrics. Photon. Res. **11**(2), 290 (2023). 10.1364/prj.476109

[141] X. Liu, P. Wang, C. Xiao, L. Fu, H. Zhou et al., A bioinspired bilevel metamaterial for multispectral manipulation toward visible, multi-wavelength detection lasers and mid-infrared selective radiation. Adv. Mater. **35**(41), 2302844 (2023). 10.1002/adma.202302844 10.1002/adma.202302844 37402134

[142] Z. Qin, C. Zhang, Z. Liang, D. Meng, X. Shi et al., Thin multispectral camouflage absorber based on metasurfaces with wide infrared radiative cooling window. Adv. Photonics Res. **3**(5), 2100215 (2022). 10.1002/adpr.202100215

[143] X. Liu, P. Wang, C. Xiao, L. Fu, J. Xu et al., Compatible stealth metasurface for laser and infrared with radiative thermal engineering enabled by machine learning. Adv. Funct. Mater. **33**(11), 2212068 (2023). 10.1002/adfm.202212068

[144] S. Dang, H. Ye, A visible-infrared-compatible camouflage photonic crystal with heat dissipation by radiation in 5–8 μm. Cell Rep. Phys. Sci. **2**(11), 100617 (2021). 10.1016/j.xcrp.2021.100617

[145] J. Zhou, Z. Zhan, F. Zhu, Y. Han, Preparation of flexible wavelength-selective metasurface for infrared radiation regulation. ACS Appl. Mater. Interf. **15**(17), 21629–21639 (2023). 10.1021/acsami.3c01452 10.1021/acsami.3c01452 37094293

[146] Z. Deng, W. Hu, P. Zhou, L. Huang, T. Wang et al., Broadband tunable laser and infrared camouflage by wavelength-selective scattering metamaterial with radiative thermal management. Opt. Lett. **49**(4), 935–938 (2024). 10.1364/OL.512245 38359220 10.1364/OL.512245

[147] T. Inamori, N. Ozaki, P. Saisutjarit, H. Ohsaki, Passive radiative cooling of a HTS coil for attitude orbit control in micro-spacecraft. Adv. Space Res. **55**(4), 1211–1221 (2015). 10.1016/j.asr.2014.10.035

[148] K.A.J. Doherty, B. Twomey, S. McGlynn, N. MacAuliffe, A. Norman et al., High-temperature solar reflector coating for the solar orbiter. J. Spacecr. Rockets **53**(6), 1077–1084 (2016). 10.2514/1.a33561

[149] X.-F. Pan, B. Wu, H.-L. Gao, S.-M. Chen, Y. Zhu et al., Double-layer nacre-inspired polyimide-*Mica nanocomposite* films with excellent mechanical stability for LEO environmental conditions. Adv. Mater. **34**(2), 2105299 (2022). 10.1002/adma.202105299 10.1002/adma.202105299 34802169

[150] A.K. Sharma, N. Sridhara, Degradation of thermal control materials under a simulated radiative space environment. Adv. Space Res. **50**(10), 1411–1424 (2012). 10.1016/j.asr.2012.07.010

[151] K.A. Watson, F.L. Palmieri, J.W. Connell, Space environmentally stable polyimides and copolyimides derived from [2, 4-bis(3-aminophenoxy)phenyl] diphenylphosphine oxide. Macromolecules **35**(13), 4968–4974 (2002). 10.1021/ma0201779

[152] Y. Zhang, J. Yu, *In situ* formation of SiO_2_ nanospheres on common fabrics for broadband radiative cooling. ACS Appl. Nano Mater. **4**(10), 11260–11268 (2021). 10.1021/acsanm.1c02841

[153] M. Liu, X. Li, L. Li, L. Li, S. Zhao et al., Continuous photothermal and radiative cooling energy harvesting by VO_2_ smart coatings with switchable broadband infrared emission. ACS Nano **17**(10), 9501–9509 (2023). 10.1021/acsnano.3c01755 37166276 10.1021/acsnano.3c01755

[154] G. Chen, Y. Wang, Y. Zou, H. Wang, J. Qiu et al., Hexagonal boron nitride and alumina dual-layer coating for space solar thermal shielding. Chem. Eng. J. **421**, 127802 (2021). 10.1016/j.cej.2020.127802

[155] C. Ibekwe, X. Wang, B.N. Bolzani, C. O’Brien, C.J. Waataja et al., Synthesis, optical performance characterization, and durability of electrospun PTFE-PEO materials for space applications. ACS Appl. Mater. Interfaces **16**(25), 32587–32598 (2024). 10.1021/acsami.4c02463 38771585 10.1021/acsami.4c02463

[156] G. Chen, Y. Wang, J. Qiu, J. Cao, Y. Zou et al., Robust inorganic daytime radiative cooling coating based on a phosphate geopolymer. ACS Appl. Mater. Interf. **12**(49), 54963–54971 (2020). 10.1021/acsami.0c15799 10.1021/acsami.0c15799 33226211

[157] W. Xiao, P. Dai, H.J. Singh, I.A. Ajia, X. Yan et al., Flexible thin film optical solar reflectors with Ta_2_O_5_-based multimaterial coatings for space radiative cooling. APL Photonics **8**(9), 090802 (2023). 10.1063/5.0156526

[158] Z. Ding, X. Li, H. Zhang, D. Yan, J. Werlé et al., Robust radiative cooling *via* surface phonon coupling-enhanced emissivity from SiO_2_ micropillar arrays. Int. J. Heat Mass Transf. **220**, 125004 (2024). 10.1016/j.ijheatmasstransfer.2023.125004

[159] C. Fu, M. Zhu, D. Liu, D. Zhao, X. Zhang, Multilayer nanoparticle-polymer metamaterial for radiative cooling of the stratospheric airship. Adv. Space Res. **72**(2), 541–551 (2023). 10.1016/j.asr.2023.03.004

[160] Y. Peng, J. Dong, Y. Gu, Y. Zhang, J. Long et al., Smart temperature-adaptive thermal regulation textiles integrating passive radiative cooling and reversible heat storage. Nano Energy **131**, 110311 (2024). 10.1016/j.nanoen.2024.110311

[161] R. Liu, K. Xia, T. Yu, F. Gao, Q. Zhang et al., Multifunctional smart fabrics with integration of self-cleaning, energy harvesting, and thermal management properties. ACS Nano **18**(45), 31085–31097 (2024). 10.1021/acsnano.4c08324 39480157 10.1021/acsnano.4c08324

[162] K. Zhu, H. Yao, J. Song, Q. Liao, S. He et al., Temperature-adaptive dual-modal photonic textiles for thermal management. Sci. Adv. **10**(41), eadr2062 (2024). 10.1126/sciadv.adr2062 39383222 10.1126/sciadv.adr2062 PMC11463281

[163] X.A. Zhang, S. Yu, B. Xu, M. Li, Z. Peng et al., Dynamic gating of infrared radiation in a textile. Science **363**(6427), 619–623 (2019). 10.1126/science.aau1217 30733415 10.1126/science.aau1217

[164] Y. Ding, Z. Mei, X. Wu, W. Zhang, Y. Zhang et al., Integrated multispectral modulator with efficient radiative cooling for innovative thermal camouflage. Adv. Funct. Mater. (2025). 10.1002/adfm.202500122

[165] S. Yang, Q. Li, B. Du, Y. Ying, Y. Zeng et al., Photothermal superhydrophobic copper nanowire assemblies: fabrication and deicing/defrosting applications. Int. J. Extrem. Manuf. **5**(4), 045501 (2023). 10.1088/2631-7990/acef78

[166] J. Yong, Q. Yang, J. Huo, X. Hou, F. Chen, Underwater gas self-transportation along femtosecond laser-written open superhydrophobic surface microchannels (<100 µm) for bubble/gas manipulation. Int. J. Extrem. Manuf. **4**(1), 015002 (2022). 10.1088/2631-7990/ac466f

[167] J. Mandal, Y. Fu, A.C. Overvig, M. Jia, K. Sun et al., Hierarchically porous polymer coatings for highly efficient passive daytime radiative cooling. Science **362**(6412), 315–319 (2018). 10.1126/science.aat9513 30262632 10.1126/science.aat9513

[168] N. Guo, C. Shi, N. Warren, E.A. Sprague-Klein, B.W. Sheldon et al., Challenges and opportunities for passive thermoregulation. Adv. Energy Mater. **14**(34), 2401776 (2024). 10.1002/aenm.202401776

[169] B.-X. Li, Z. Luo, W.-G. Yang, H. Sun, Y. Ding et al., Adaptive and adjustable MXene/reduced graphene oxide hybrid aerogel composites integrated with phase-change material and thermochromic coating for synchronous visible/infrared camouflages. ACS Nano **17**(7), 6875–6885 (2023). 10.1021/acsnano.3c00573 36996266 10.1021/acsnano.3c00573

[170] B.-X. Li, Z. Luo, H. Sun, Q. Quan, S. Zhou et al., Spectral-selective and adjustable patterned polydimethylsiloxane/MXene/nanoporous polytetrafluoroethylene metafabric for dynamic infrared camouflage and thermal regulation. Adv. Funct. Mater. **34**(45), 2407644 (2024). 10.1002/adfm.202407644

[171] Z. Lei, B. Wu, P. Wu, Hierarchical network-augmented hydroglasses for broadband light management. Research **2021**, 4515164 (2021). 10.34133/2021/4515164 33623918 10.34133/2021/4515164 PMC7877396

[172] Y. Hu, H. Liu, B. Yang, K. Shi, M. Antezza et al., Enhanced near-field radiative heat transfer between core-shell nanoparticles through surface modes hybridization. Fundam. Res. **4**(5), 1092–1099 (2024). 10.1016/j.fmre.2023.03.021 39431123 10.1016/j.fmre.2023.03.021 PMC11489492

[173] H. Yin, X. Zhou, Z. Zhou, R. Liu, X. Mo et al., Switchable kirigami structures as window envelopes for energy-efficient buildings. Research **6**, 0103 (2023). 10.34133/research.0103 37223463 10.34133/research.0103 PMC10202178

[174] M. Li, D. Liu, H. Cheng, L. Peng, M. Zu, Manipulating metals for adaptive thermal camouflage. Sci. Adv. **6**(22), eaba3494 (2020). 10.1126/sciadv.aba3494 32518826 10.1126/sciadv.aba3494 PMC7253164

[175] J. Mandal, M. Jia, A. Overvig, Y. Fu, E. Che et al., Porous polymers with switchable optical transmittance for optical and thermal regulation. Joule **3**(12), 3088–3099 (2019). 10.1016/j.joule.2019.09.016

[176] S. Wang, T. Jiang, Y. Meng, R. Yang, G. Tan et al., Scalable thermochromic smart windows with passive radiative cooling regulation. Science **374**(6574), 1501–1504 (2021). 10.1126/science.abg0291 34914526 10.1126/science.abg0291

[177] M. Shi, Z. Song, J. Ni, X. Du, Y. Cao et al., Dual-mode porous polymeric films with coral-like hierarchical structure for all-day radiative cooling and heating. ACS Nano **17**(3), 2029–2038 (2023). 10.1021/acsnano.2c07293 36638216 10.1021/acsnano.2c07293

[178] S.K. Saju, A.B. Puthirath, S. Wang, T. Tsafack, L.K. Beagle et al., Thermochromic polymer blends. Joule **8**(9), 2696–2714 (2024). 10.1016/j.joule.2024.07.020

[179] K. Sun, W. Xiao, C. Wheeler, M. Simeoni, A. Urbani et al., VO_2_ metasurface smart thermal emitter with high visual transparency for passive radiative cooling regulation in space and terrestrial applications. Nanophotonics **11**(17), 4101–4114 (2022). 10.1515/nanoph-2022-0020 39635180 10.1515/nanoph-2022-0020 PMC11502091

[180] J.-W. Ma, F.-R. Zeng, X.-C. Lin, Y.-Q. Wang, Y.-H. Ma et al., A photoluminescent hydrogen-bonded biomass aerogel for sustainable radiative cooling. Science **385**(6704), 68–74 (2024). 10.1126/science.adn5694 38963855 10.1126/science.adn5694

[181] Y. Zhou, C. Lu, R. Xiong, Hierarchical nanocellulose photonic design for synergistic colored radiative cooling. ACS Nano **19**(4), 5029–5039 (2025). 10.1021/acsnano.5c00330 39862205 10.1021/acsnano.5c00330

[182] X. Xue, M. Qiu, Y. Li, Q.M. Zhang, S. Li et al., Creating an eco-friendly building coating with smart subambient radiative cooling. Adv. Mater. **32**(42), e1906751 (2020). 10.1002/adma.201906751 32924184 10.1002/adma.201906751

[183] S. Kim, J.H. Park, J.W. Lee, Y. Kim, Y.T. Kang, Self-recovering passive cooling utilizing endothermic reaction of NH_4_NO_3_/H_2_O driven by water sorption for photovoltaic cell. Nat. Commun. **14**(1), 2374 (2023). 10.1038/s41467-023-38081-9 37185269 10.1038/s41467-023-38081-9 PMC10130129

[184] S. Kim, S. Lee, J. Lee, H.W. Choi, W. Choi et al., Passive isothermal film with self-switchable radiative cooling-driven water sorption layer for arid climate applications. Nat. Commun. **15**(1), 8000 (2024). 10.1038/s41467-024-52328-z 39266577 10.1038/s41467-024-52328-z PMC11392948

